# Efficacy and Safety of Traditional Chinese Medicine Injections for Heart Failure With Reduced Ejection Fraction: A Bayesian Network Meta-Analysis of Randomized Controlled Trials

**DOI:** 10.3389/fphar.2021.659707

**Published:** 2021-11-30

**Authors:** Shanshan Lin, Qingyang Shi, Zhao Ge, Yangxi Liu, Yawen Cao, Ying Yang, Zhiqiang Zhao, Yingfei Bi, Yazhu Hou, Shuai Wang, Xianliang Wang, Jingyuan Mao

**Affiliations:** ^1^ National Clinical Research Center for Chinese Medicine Acupuncture and Moxibustion, First Teaching Hospital of Tianjin University of Traditional Chinese Medicine, Tianjin, China; ^2^ Tianjin University of Traditional Chinese Medicine, Tianjin, China

**Keywords:** traditional chinese medicine, traditional chinese medicine injection, heart failure, randomized controlled trial, network meta-analysis, bayesian model

## Abstract

**Background:** Heart failure as an important issue in global public health, has brought a heavy economic burden. Traditional Chinese medicine injections (TCMIs) have significant effects on heart failure with reduced ejection fraction (HFrEF). However, it is difficult for clinicians to identify the differences in clinical efficacy and safety of various TCMIs. The purpose of this study is to compare the efficacy and safety of various TCMIs for treating HFrEF by conducting a Bayesian network meta-analysis (NMA) and to further provide references for clinical decision-making.

**Methods:** The clinical randomized controlled trials of TCMIs for treating HFrEF were searched in seven database from inception to August 3rd, 2021. The Cochrane collaboration’s tool was used to assess the risk of bias. NMA was performed in a Bayesian hierarchical framework. The surface under the cumulative ranking curve (SUCRA), the multi-dimensional efficacy analysis, the comparison-adjusted funnel plot, and the node-splitting analysis were conducted using R software.

**Results:** A total of 107 eligible RCTs involving 9,073 HFrEF patients and 6 TCMIs were included. TCMIs include Huangqi injection (HQ) also called *Astragalus* injection, Shenfu injection (SF), Shengmai injection (SGM), Shenmai injection (SM), Xinmailong injection (XML), and Yiqifumai lyophilized injection (YQFM). The results of NMA and SUCRA showed that with conventional treatment (CT) as a common control, in terms of clinical efficacy, CT + XML was most effective in New York Heart Association cardiac functional classification efficiency, brain natriuretic peptide, and N-terminal pro-brain natriuretic peptide; the CT + SM was most effective in 6-min walking test, left ventricular end-diastolic diameter, left ventricular end-systolic diameter and cardiac output; the CT + YQFM was most effective in left ventricular ejection fraction; the CT + HQ was most effective in stroke volume; the CT + SF was most effective in Minnesota Living with Heart Failure Questionnaire. In terms of safety, there was no significant difference between CT + TCMIs and CT.

**Conclusion:** This Bayesian network meta-analysis results show that the combination of qualified TCMIs and CT is more effective for HFrEF patients than CT alone, and CT + XML and CT + SM may be one of the potential optimal treatments. Also, the safety of these TCMIs needs to be further observed. However, due to some limitations, the conclusions need to be verified by more large-sample, double-blind, multi-center RCTs.

## Introduction

Heart failure (HF) is defined as a clinical syndrome, characterized by dyspnea, fatigue, fluid retention, etc., caused by a reduced cardiac output and/or elevated intracardiac pressures due to a structural and/or functional cardiac abnormality (Ponikowski et al., 2016). It generally occurs in the terminal stage of various cardiovascular diseases, with high morbidity and mortality (Virani et al., 2020). A China Heart Failure Registry Study (China-HF) conducted between 2012 and 2014 indicated that the in-hospital mortality of HF patients was 5.3% (Zhang and Zhang, 2015). As standard treatment strategies for HF are constantly being updated and improved, the morbidity and mortality of HF have decreased slightly (Merlo et al., 2014). However, with the intensified aging of the social demographic structure, the total proportion of HF is expected to rise from 2.42% in 2012 to 2.97% in 2030. The overall cost of HF is continuing to rise, which will increase the global medical and health burden (Heidenreich et al., 2013; Cook et al., 2014; Dokainish et al., 2015; Virani et al., 2020). Therefore, how to further improve the quality of life and long-term prognosis of HF patients based on modern medicine has attracted increasing attention.

Traditional Chinese medicine (TCM) is an empirical medicine with a history of more than 2,000 years and has considerable research value. Especially in the prevention and treatment of coronavirus disease in 2019 (COVID-19), TCM has played an irreplaceable role. Therefore, the clinical value of TCM is worthy of continuous exploration, and it needs more attention from all over the world. Compared with TCM decoctions, which are generally recognized as the most traditional medicine dosage form, the effective ingredients of traditional Chinese medicine injections (TCMIs) can be directly injected into the blood through intravenous injection. On the one hand, it takes effect faster, and on the other hand, it reduces the possibility of the drug being metabolized by the digestive system, thereby ensuring sufficient active ingredients to work. Besides, as modern preparations of TCM extracts, TCMIs have a more standard quality evaluation system than TCM decoctions, so it is easier to obtain high-quality clinical evidence. At present, it has been proved that a large number of TCMIs can significantly improve the clinical efficacy of heart failure with reduced ejection fraction (HFrEF) based on conventional treatment (CT) (Xu T. et al., 2018; Zhu et al., 2018; Lin W. J. et al., 2019; Xie and Dai, 2019; Lin et al., 2021). However, due to the lack of direct comparative evidence, it is difficult for clinicians to identify the differences in clinical efficacy and safety of various TCMIs and prescribe optimal drugs.

Network meta-analysis (NMA) can compare multiple interventions simultaneously in a single analysis by synthesizing direct and indirect evidence, and ranking them according to efficacy or safety (Rouse et al., 2017). Therefore, this NMA compared the efficacy and safety of various TCMIs in the treatment of HFrEF performed in a Bayesian hierarchical framework (Jansen et al., 2008; Jonas et al., 2013), and explored the advantages of various TCMIs to provide references for clinical decision-making.

## Information and Methods

The protocol of this study has been registered in PROSPERO (registration number: CRD42020166900) and has been published in an open-access journal (Lin et al., 2020). The NMA was performed in accordance with the Preferred Reporting Items for Systematic Review and Meta-Analysis (PRISMA) extension statement for reporting of systematic reviews incorporating network meta-analyses of healthcare interventions (Hutton et al., 2015). See Supplementary File S1 for a completed PRISMA checklist.

### Inclusion and Exclusion Criteria

#### Participants

The population was HF patients in the stable phase or acute exacerbation phase. The patient met the recognized diagnostic criteria, such as the “*CCS/CHFS Heart Failure Guidelines Update: Defining a New Pharmacologic Standard of Care for Heart Failure With Reduced Ejection Fraction*” (McDonald et al., 2021), the “*Guidelines for diagnosis and treatment of heart failure in China 2018*” (Heart Failure Group of Chinses Society of Cardiology of Chinses Medical Association et al., 2018), the “*2016 ESC Guidelines for the diagnosis and treatment of acute and chronic heart failure*” (Ponikowski et al., 2016) and so on, while meeting the condition of LVEF <50%. Primary diseases included coronary heart disease, hypertension, dilated cardiomyopathy, and rheumatic heart disease. Sex, age, race, and source of the case were not limited.

#### Interventions and Comparisons

① Conventional treatment for HF (CT_HF_) + TCMI vs. CT_HF_ (with or without placebo); ② CT_HF_ + TCMI_a_ vs. CT_HF_ + TCMI_b_. In the control group and the test group, the CT_HF_ is the same, and both follow national or international guidelines. TCMIs have been approved by the China Food and Drug Administration, and the drug instructions specifically defined its indications including HF or other acute and chronic diseases caused by cardiac insufficiency.

#### Outcomes

• Primary outcomes: ① New York Heart Association (NYHA) cardiac functional classification efficiency: according to the NYHA cardiac function classification standard, the degree of improvement of cardiac function was rated as “markedly effective” (NYHA grade increased by ≥2), “effective” (NYHA grade increased by 1), or “ineffective” (NYHA grade remained unchanged or even deteriorated). Total effective rate = (“markedly effective” patients + “effective” patients)/all patients × 100%. ② 6-min walking test (6MWT).
• Secondary outcomes: ① left ventricular ejection fraction (LVEF); ② left ventricular end-diastolic diameter (LVEDD); ③ left ventricular end-systolic diameter (LVESD); ④ cardiac output (CO); ⑤ stroke volume (SV); ⑥ brain natriuretic peptide (BNP); ⑦ N-terminal pro-brain natriuretic peptide (NT-proBNP); ⑧ Minnesota Living with Heart Failure Questionnaire (MLHFQ); ⑨ adverse drug event: any untoward medical occurrence during treatment.

#### Type of Study

Clinical randomized controlled trials (RCTs).

#### Exclusion Criteria

• Participants were any of the following: HF with malignant arrhythmias, malignant tumors, hypothyroidism, severe liver and kidney dysfunction, or severe infections.
• Study with imbalanced or incomparable baseline data between the two groups.
• The study cannot be integrated because of incorrect data or incomplete information.
• For republished studies, choose the one with the most complete data.
• The full text cannot be obtained after seeking help online or contacting the corresponding author via email.
• Unfinished protocol.

### Search Strategy

The clinical RCTs of TCMIs for treating HFrEF were searched in the relevant database, including PubMed, Embase, Cochrane Library, Chinese BioMedical Literature Database (CBM), China National Knowledge Infrastructure (CNKI), Wanfang Database, and VIP Chinese Science and Technology Journal Database (VIP). The retrieval time was from inception to August 3rd, 2021. According to the instructions, TCMIs that meet the inclusion criteria included Huangqi injection (HQ) also called *Astragalus* injection, Shenfu injection (SF), Shengmai injection (SGM), Shenmai injection (SM), Xinmailong injection (XML), and Yiqifumai lyophilized injection (YQFM). The search strategy was developed according to the Cochrane Handbook for Systematic Reviews (Higgins and Thomas, 2020) by two researchers (SSL with clinical work experience and QYS with evidence-based work experience). Search terms included heart failure, names of TCMIs that have been included, randomized controlled trial, and their synonyms. Besides, the reference lists of related documents were tracked to avoid omission. Take PubMed as an example. The detailed search strategy is shown in Supplementary File S2.

### Literature Screening, Data Extraction, and Risk of Bias Assessment

Records from databases were managed and screened using NoteExpress software (V3.2.0). The data extraction items included publication information (title, first author, journal name, publication year), participants’ characteristics (diagnostic criteria, sample size, sex, age, ethnicity, case source, and baseline status), interventions (drug name, medication route, drug dose, course of treatment, and patient compliance), outcomes, risk of bias information, and others. The Cochrane Collaboration’s tool (Higgins et al., 2011) was used to assess the risk of bias of included RCTs from the following seven items: ① random sequence generation; ② allocation concealment; ③ blinding participants and personnel; ④ blinding outcome assessment; ⑤ incomplete outcome data; ⑥ selective reporting; and ⑦ other bias. The results of the risk of bias assessment included the low risk of bias, the high risk of bias, and the unclear risk of bias. Data extraction and risk of bias assessment were independently completed and cross-checked by multiple researchers. Disagreements were resolved with the assistance of other researchers. The risk of bias graph was generated by Microsoft Excel.

### Statistical Analysis

A Bayesian model was used to conduct NMAs according to the Markov chain Monte Carlo method (Jansen et al., 2008). Dichotomous variables were presented as the relative risk (RR) or odds ratio (OR) with a 95% credible interval (CrI). Continuous variables were presented as the weight mean difference (WMD) with a 95% CrI. The *χ* ^2^ test and *Ι* ^2^ test were conducted to assess the potential heterogeneity. *p* < 0.05 was considered statistically significant. The random-effects model was selected to synthesize the data. The network diagram of each outcome was performed to visualize the connections between different interventions. The surface under the cumulative ranking curve (SUCRA) was performed to rank different interventions (Rücker and Schwarzer, 2015). The multi-dimensional efficacy analysis was performed to integrate the results of multiple outcomes to obtain the optimal intervention. The comparison-adjusted funnel plot was plotted to detect small sample size study effects and publication bias. The node-splitting analysis was used to split mixed evidence into direct evidence and indirect evidence to evaluate the consistency of the model. All analyses were conducted using R software (V3.6.1).

## Results

### Literature Screening Result

**FIGURE 1:**
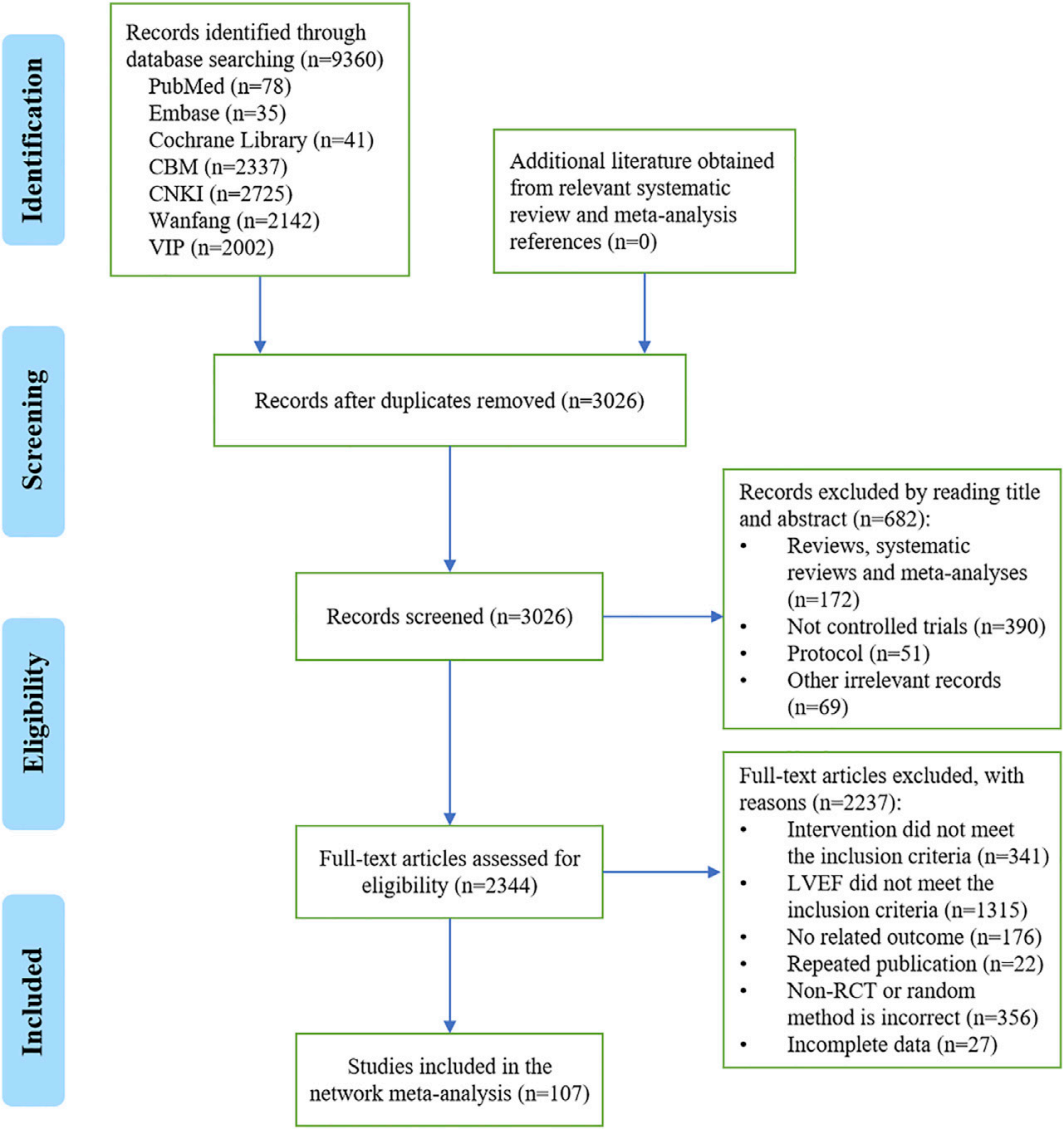
Flowchart of the literature screening process. CBM, Chinese BioMedical Literature Database; CNKI, China National Knowledge Infrastructure; VIP, VIP Chinese Science and Technology Journal Database; LVEF, left ventricular ejection fraction; RCT, randomized controlled trial; *n*, the number of articles.

A total of 9,360 records were initially obtained, and 2,344 possible related records were identified by reading the title and abstract. After reading the full text, 107 eligible studies were included in this NMA. The details of the literature screening process are shown in Figure 1.

### Basic Characteristics of Included Studies

**FIGURE 2:**
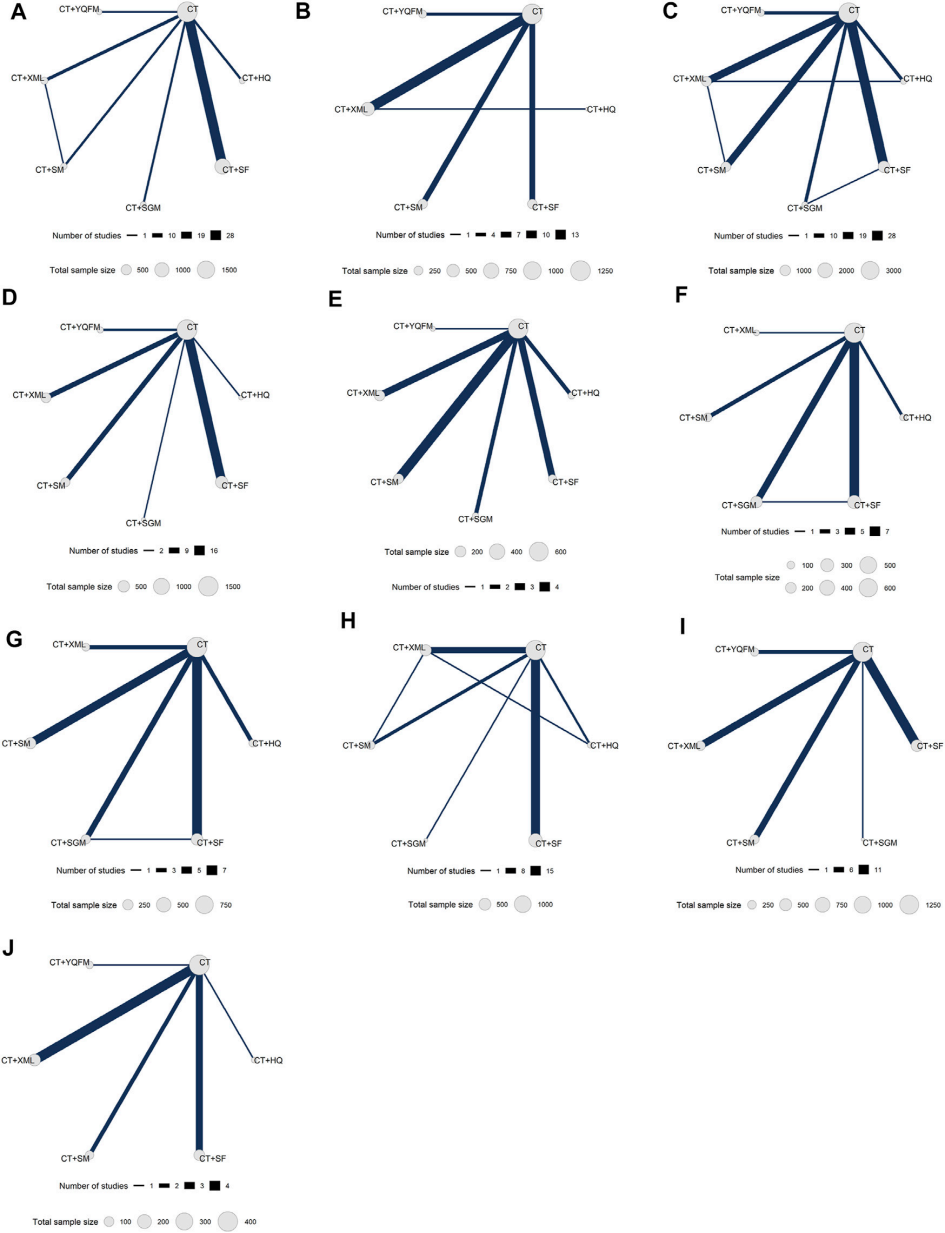
Network diagrams for different outcomes. **(A)** New York Heart Association cardiac functional classification efficiency; **(B)** 6-min walking test; **(C)** left ventricular ejection fraction; **(D)** left ventricular end-diastolic diameter; **(E)** left ventricular end-systolic diameter; **(F)** cardiac output; **(G)** stroke volume; **(H)** brain natriuretic peptide; **(I)** N-terminal pro-brain natriuretic peptide; **(J)** Minnesota Living with Heart Failure Questionnaire. CT, conventional treatment; HQ, Huangqi injection; SF, Shenfu injection; SGM, Shengmai injection; SM, Shenmai injection; XML, Xinmailong injection; YQFM, Yiqifumai lyophilized injection.

One hundred and seven RCTs involving 9,073 HFrEF patients were included, conducted in China from 2003 to 2021. Most RCTs focused on SF (40 RCTs, 37.38%) (Que and Guo, 2003; Song et al., 2006; Chen et al., 2009; Hu et al., 2009; Huang, 2009; Wu and Duan, 2009; Yang and Wu, 2009; Zhu, 2009; Lv, 2010; Wu and Wang, 2010; Yao and Lu, 2010; Bao and Yu, 2011; Wang et al., 2011; Wu et al., 2011; Dong, 2012; Ding, 2013; Wang et al., 2014; Xu et al., 2014; Yu et al., 2014; Zhang et al., 2014; Pan, 2015; Qi, 2015; Qi and Qi, 2015; Xu et al., 2015; Mao, 2016; Wang et al., 2016; Wang and Pan, 2016; Wu, 2016; Yao et al., 2016; Zhang and Wen, 2016; Deng and Bai, 2017; He, 2017; Su, 2017; Yan et al., 2017; Zhang, 2017; Han, 2018; Li L. J., 2018; Liu et al., 2018; Zhang et al., 2018; Li J. P et al., 2020), followed by XML (23 RCTs, 21.50%) (Wu et al., 2012; Tang et al., 2013; Peng et al., 2014; Su et al., 2014; Yao et al., 2014; Zhao et al., 2014; Feng et al., 2015; Liu, 2015; Liu et al., 2015; Zhong, 2016; Wang, 2017; Li X. L., 2018; Su et al., 2018; Xu S.L. et al., 2018; Zhang, 2018; Hao, 2019; Hao et al., 2019; Mao, 2019; Wang, 2019; Xue et al., 2019; Li, 2020; Xi, 2020; Wang et al., 2021), SM (22 RCTs, 20.56%) (Chen and Zhou, 2010; Huang et al., 2011; Lan and Wei, 2011; Wang and Wu, 2012; Li, 2013; Lin and Bao, 2013; Zhai, 2013; Wang, 2014; Yu et al., 2015; Li et al., 2016; Yang, 2016; Yang et al., 2016; He et al., 2018; Li L, 2018; Xu E. W. et al., 2018; An and Xiao, 2019; Cui et al., 2019; Wang, 2019; He et al., 2020; Chen 2021; Jiang 2021; Zhang et al., 2021), SGM (10 RCTs, 9.35%) (Chen et al., 2008; Liu, 2008; Lu et al., 2008; Zhang, 2008; Kong, 2010; Xu et al., 2011; Ding et al., 2012; Pan, 2013; Qi, 2015; Li J. F., 2018), HQ (8 RCTs, 7.48%) (Liu and Fu, 2003; Wang, 2003; Wu and Chen, 2005; Guo and Chen, 2006; Xing and Nie, 2006; Su et al., 2014; Sun, 2016; Gan, 2019), and YQFM (7 RCTs, 6.54%) (Yu and Wang, 2012; Yuan and Du, 2012; Li, 2014; Guo, 2015; Zhao, 2015; Tao et al., 2020; Kou 2021). As recommended by the international HF guidelines (Ponikowski et al., 2016; Heart Failure Group of Chinses Society of Cardiology of Chinses Medical Association et al., 2018; McDonald et al., 2021), CT_HF_ included angiotensin-converting enzyme inhibitor (ACEI), angiotensin receptor blocker (ARB), β-blocker, mineralocorticoid receptor antagonists, diuretics, vasodilators, cardiotonic drugs, antiplatelet drugs, anticoagulant drugs, and so on. There was slight variability in the medication depending on the patient’s condition. However, the medication principles of all RCTs followed the recommendations of the guidelines. The course of treatment ranged from 5 days to 12 weeks. Details are shown in Table 1. The network diagrams of the above 6 TCMIs with different outcomes are shown in Figure 2.

**TABLE 1:** Characteristics of the studies included in this network meta-analysis.

Study ID	LVEF	Sample size		Sex (M/F)		Age (year)^a^		Interventions		Course of treatment	Outcomes
		T	C	T	C	T	C	T	C		
Xing and Nie, (2006)	<40%	22	21	30/13		60.0 ± 15.6		CT_HF_^#^ + HQ(50 ml,qd)	CT_HF_	14 d	③④⑤
Guo and Chen, (2006)	<40%	42	42	-		52.3(40∼74)		CT_HF_ + HQ(30 ml,qd)	CT_HF_	10 d	①③⑦
Wang, (2003)	≤45%	35	31	26/9	24/7	60.9 ± 11.7	60.6 ± 10.6	CT_HF_ + HQ(40 ml,qd)	CT_HF_ (e.g., metoprolol tablets 6.25 mg, bid)	14 d	③
Liu and Fu, (2003)	<45%	62	50	38/24	31/19	58 ± 18	59 ± 19	CT_HF_ + HQ(30 ml,qd)	CT_HF_ (e.g., captopril 12.5 mg, bid; digoxin 0.125 mg, qd; hydrochlorothiazide 25 mg, qd; isosorbide dinitrate 10 mg, qd)	10 d	①③⑥⑦
Wu and Chen, (2005)	<45%	26	26	15/11	13/13	39.10 ± 17.92	41.20 ± 16.81	CT_HF_ + HQ(30 ml,qd)	CT_HF_	15 d	①③
Sun, (2016)	<45%	54	54	25/29	27/27	68.0 ± 8.9	67.0 ± 8.8	CT_HF_ + HQ(40 ml,qd)	CT_HF_	14 d	③④⑤⑧
Gan, (2019)	≤40%	30	30	18/12	16/14	59.3 ± 5.7	59.8 ± 5.6	CT_HF_ + HQ(20 ml,qd)	CT_HF_ (e.g., metoprolol tartrate sustained-release tablets 25 mg, qd)	14 d	①③⑥⑦⑩
Hu et al. (2009)	≤40%	31	32	12/19	12/20	72.94 ± 7.58	76.43 ± 6.80	CT_HF_ + SF(40 ml,qd)	CT_HF_	7 d	③⑥⑦
Wang et al. (2016)	<50%	26	30	13/13	16/14	71.56 ± 2.47	70.23 ± 1.56	CT_HF_ + SF(60 ml,qd)	CT_HF_ (e.g., digoxin 0.125 mg; spironolaCTone tablets 20 mg; aspirin enteric-coated tablets 100 mg; metoprolol tartrate tablets 12.5 mg; isosorbide dinitrate tablets 5 mg; fosinopril sodium tablets 10 mg orally; furosemide tablets 20 mg, qd)	10 ± 2 d	①⑨⑪
Zhang et al. (2014)	<45%	40	40	21/19	23/17	49.9 ± 13.8	52.1 ± 12.6	CT_HF_ + SF(50 ml,qd)	CT_HF_	14 d	①②⑧
Wang and Pan, (2016)	≤40%	32	29	12/20	11/18	73.16 ± 12.25	32.35 ± 11.09	CT_HF_ + SF(20 ml,qd)	CT_HF_	14 d	③④⑦⑨
Wu, (2016)	≤40%	43	43	52/34		63.2 ± 7.3		CT_HF_ + SF(10 ml,qd)	CT_HF_	14 d	③④⑤⑨
Xu et al. (2015)	≤40%	25	25	15/10	17/8	64.00 ± 11.00	63.00 ± 10.00	CT_HF_ + SF(100 ml,qd)	CT_HF_	7 d	①③④⑨⑩
Wu et al. (2011)	≤40%	31	32	12/19	12/20	72.94 ± 7.58	76.43 ± 6.80	CT_HF_ + SF(40 ml,qd)	CT_HF_	7 d	③④⑤⑨
Zhang and Wen, (2016)	<50%	30	30	17/13	18/12	65.4 ± 7.8	64.8 ± 8.7	CT_HF_ + SF(50 ml,qd)	CT_HF_	14 d	③④
Yao et al. (2016)	≤40%	51	51	23/28	21/30	71.73 ± 8.205	71.22 ± 8.218	CT_HF_ + SF(40 ml,qd)	CT_HF_	14 d	⑩
Dong, (2012)	<40%	30	30	16/14	15/15	59.8 ± 10.2	61.7 ± 10.6	CT_HF_ + SF(50 ml,qd)	CT_HF_	14 d	①⑪
Mao, (2016)	≤40%	100	100	116/84		56.2(46∼77)		CT_HF_ + SF(40 ml,qd)	CT_HF_	5∼10 d	①⑨
Zhang et al. (2018)	≤40%	110	110	67/43	62/48	49.52 ± 15.25	48.77 ± 13.62	CT_HF_ + SF(50 ml,qd)	CT_HF_	14 d	③④⑧⑪
Wu and Duan, (2009)	≤40%	33	29	17/16	14/15	71.48 ± 5.78	73.59 ± 6.96	CT_HF_ + SF(50 ml,qd)	CT_HF_	14 d	①③④⑦⑧⑪
Lv, (2010)	<45%	31	30	22/9	22/8	41.5 ± 10.2	40.3 ± 12.5	CT_HF_ + SF(50 ml,qd)	CT_HF_	14 d	①②④⑧
Qi and Qi, (2015)	<45%	60	60	40/20	42/18	63.00(45∼85)	62.70(44∼87)	CT_HF_ + SF(60 ml,qd)	CT_HF_	14 d	①③⑥⑦⑨
Yang and Wu, (2009)	<45%	30	30	19/11	23/7	42.50 ± 16.81	40.52 ± 15.98	CT_HF_ + SF(60 ml,qd)	CT_HF_	14 d	①③⑪
Chen et al. (2009)	<45%	30	27	22/8	20/7	21∼75	20∼76	CT_HF_ + SF(50 ml,qd)	CT_HF_	14 d	①③④⑧
Wang et al. (2014)	≤45%	42	38	26/16	23/15	61.8 ± 10.3	60.8 ± 9.9	CT_HF_ + SF(50 ml,qd)	CT_HF_	10 d	①②③⑧⑩
Wu and Wang, (2010)	<45%	44	44	24/20	25/19	70.4 ± 7.2	72.7 ± 7.2	CT_HF_ + SF(100 ml,qd)	CT_HF_	14 d	①③④⑧
Bao and Yu, (2011)	≤45%	30	30	32/28		53.6 ± 9.8		CT_HF_ + SF(50 ml,qd)	CT_HF_	14 d	①⑪
Yao and Lu, (2010)	≤45%	30	30	33/27		53.6 ± 9.8		CT_HF_ + SF(50 ml,qd)	CT_HF_	14 d	①③④⑥⑦⑪
Ding, (2013)	<50%	40	40	19/21	22/18	72.94 ± 7.58	76.43 ± 6.80	CT_HF_ + SF(60 ml,qd)	CT_HF_	14 d	①③⑧
Song et al. (2006)	≤40%	45	42	26/19	24/18	62.13 ± 12.37	61.90 ± 12.52	CT_HF_ + SF(60 ml,qd)	CT_HF_	15 d	①③⑥⑦⑪
Zhu, (2009)	≤40%	24	22	20/26		61.7 ± 13.6		CT_HF_ + SF(50 ml,qd)	CT_HF_	14 d	②③④⑥⑧
Zhang, (2017)	<50%	30	32	22/8	20/12	56.3 ± 8.2	57.9 ± 10.1	CT_HF_ + SF(50 ml,qd)	CT_HF_	14 d	②③④⑪
Xu et al. (2014)	<40%	45	40	20/25	23/17	70.00 ± 11.30	68.00 ± 14.60	CT_HF_ + SF(80 ml,qd)	CT_HF_	10 d	①③⑥⑨⑪
Su, (2017)	≤40%	30	30	27/33		55.26 ± 9.73		CT_HF_ + SF(50 ml,qd)	CT_HF_	14 d	①②③⑧⑪
Li J. P. et al. (2020)	<45%	61	61	33/28	32/29	56.02 ± 4.14	55.98 ± 3.89	CT_HF_ + SF(20 ml,qd)	CT_HF_	14 d	③⑧
Que and Guo, (2003)	<40%	32	28	20/12	17/11	33∼75	35∼76	CT_HF_ + SF(60 ml,qd)	CT_HF_ (e.g., digoxin 0.125 mg, qd; hydrochlorothiazide 25 mg, bid; triamterene 50 mg, bid; captopril as appropriate)	14 d	①③⑥
Wang et al. (2011)	<50%	50	50	34/16	32/18	32 ± 9	31 ± 9	CT_HF_ + SF(1 ml/kg/d)	CT_HF_	14 d	③⑧
Yu et al. (2014)	<40%	75	72	83/64		35∼80		CT_HF_ + SF(50 ml,qd)	CT_HF_	14 d	①
Deng and Bai, (2017)	<50%	30	30	15/15	17/13	62.5 ± 12.6	60.3 ± 11.2	CT_HF_ + SF(50 ml,qd)	CT_HF_	14 d	①⑨
Pan, (2015)	<40%	74	74	48/26	47/27	67.3 ± 6.2	67.4 ± 5.9	CT_HF_ + SF(60 ml,qd)	CT_HF_	14 d	①②③⑧
Huang, (2009)	<40%	38	38	24/14	23/15	68.22 ± 5.53		CT_HF_ + SF(40 ml,qd)	CT_HF_	14 d	①②③④⑤⑦
Han, (2018)	≤40%	30	30	18/12	19/11	57.26 ± 6.34	57.21 ± 6.25	CT_HF_ + SF(40 ml,qd)	CT_HF_	7 d	①⑨
Li L.J., (2018)	<50%	45	45	25/20	26/19	57.2 ± 4.0	56.8 ± 3.7	CT_HF_ + SF(20 ml,qd)	CT_HF_	14 d	①③⑧
Liu et al. (2018)	<50%	51	51	-		-		CT_HF_ + SF(50 ml,qd)	CT_HF_	14 d	①③④⑨
He, (2017)	≤40%	40	40	25/15	27/13	56.6 ± 10.2	56.4 ± 10.3	CT_HF_ + SF(50 ml,qd)	CT_HF_	14 d	①
Yan et al. (2017)	≤40%	50	50	29/21	27/23	52.2 ± 11.4	51.0 ± 11.2	CT_HF_ + SF(50 ml,qd)	CT_HF_	14 d	①
Li X. L., (2018)	<45%	30	30	17/13	18/12	60.3 ± 3.5	60.2 ± 3.4	CT_HF_ + SGM(50 ml,qd)	CT_HF_ (e.g., digoxin 0.25 mg, qd; furosemide 20–40 mg, qd; dopamine, 3∼5 μg/(kg·min) as appropriate)	14 d	③④⑤⑨
Pan, (2013)	<50%	34	34	38/30		63.5 ± 7.8		CT_HF_ + SGM(60 ml,qd)	CT_HF_ (e.g., phentolamine 20 mg, qd)	14 d	③⑥⑦
Ding et al. (2012)	<45%	40	40	18/22	15/25	70.74 ± 6.43	72.56 ± 5.64	CT_HF_ + SGM(40 ml,qd)	CT_HF_	7 d	③⑥⑦⑪
Xu et al. (2011)	<40%	32	30	20/12	19/11	63.9 ± 1.3	63.5 ± 4.8	CT_HF_ + SGM(40 ml,qd)	CT_HF_	21 d	③④⑤⑥⑪
Kong, (2010)	≤40%	50	50	28/22	30/20	68.1 ± 9.1	66.7 ± 8.9	CT_HF_ + SGM(40 ml,qd)	CT_HF_	7 d	①③⑧⑪
Lu et al. (2008)	<45%	36	34	40/30		64.28(45∼79)		CT_HF_ + SGM(40 ml,qd)	CT_HF_	14 d	①⑪
Zhang, (2008)	<45%	40	40	24/16	27/13	66.45 ± 4.52	64.36 ± 3.98	CT_HF_ + SGM(60 ml,qd)	CT_HF_ (e.g., captopril 25–50 mg, tid; digoxin 0.25–0.5 mg, qd; betaloc 6.25–25 mg, tid; isosorbide mononitrate tablets 20 mg, bid.)	14 d	③⑥⑦
Liu, (2008)	<45%	60	60	84/36		64(40∼79)		CT_HF_ + SGM(1 ml/kg,qd)	CT_HF_	14 d	①
Chen et al. (2008)	<45%	36	34	-		64.28(45∼79)		CT_HF_ + SGM(1 ml/kg,qd)	CT_HF_	14 d	③⑥⑦
Zhai, (2013)	<50%	60	60	37/23	35/25	62.5 ± 6.8	61.8 ± 6.3	CT_HF_ + SM(40–60 ml,qd)	CT_HF_	14 d	③④⑨
Li et al. (2016)	<50%	60	60	37/23	35/25	61.32 ± 8.61	59.32 ± 8.35	CT_HF_ + SM(100 ml,qd)	CT_HF_	14 d	③⑦⑧
Huang et al. (2011)	<45%	60	60	32/28	33/27	67.36 ± 4.07	69.27 ± 3.96	CT_HF_ + SM(50 ml,qd)	CT_HF_	14 d	③⑧
Yang et al. (2016)	≤40%	40	40	23/17	22/18	65(58∼72)	64(56∼70)	CT_HF_ + SM(40–60 ml,qd)	CT_HF_	10 d	③⑨
Li, (2013)	<50%	68	52	37/31	29/23	63.2 ± 11.3	62.1 ± 10.2	CT_HF_ + SM(50 ml,qd)	CT_HF_	14 d	③⑥⑦⑨
Yu et al. (2015)	≤40%	40	40	18/22	20/20	35∼75	35∼75	CT_HF_ + SM(100 ml,qd)	CT_HF_	12 w	③④⑤⑨⑪
An and Xiao, (2019)	≤40%	45	45	28/17	26/19	60.12 ± 2.5	59.97 ± 2.23	CT_HF_ + SM(100 ml,qd)	CT_HF_ (e.g., irbesartan tablets 75 mg, bid)	28 d	①②③④⑤⑧
Cui et al. (2019)	<40%	49	49	23/26	29/20	59.98 ± 9.11	61.02 ± 7.69	CT_HF_ + SM(60 ml,qd)	CT_HF_	14 d	③⑨⑪
He et al. (2020)	<40%	46	46	-		61.13 ± 12.98	60.87 ± 11.94	CT_HF_ + SM(50 ml,qd)	CT_HF_	7 d	③⑦⑪
Li L., (2018)	<40%	29	29	17/12	18/11	52.0 ± 1.4	53.0 ± 1.6	CT_HF_ + SM(60 ml,qd)	CT_HF_	14 d	①③④⑪
He et al. (2018)	<40%	50	50	59/41		60.3 ± 12.4		CT_HF_ + SM(40 ml,qd)	CT_HF_ (e.g., after diluting 5 mg milrinone with 20 ml of normal saline, first pump at a rate of 40 μg/(kg·min) for 10 min, and continue to pump at a rate of 0.5 μg/(kg·min) for about 120 min, qd)	7 d	③⑦⑪
Yang, (2016)	<40%	29	29	18/11	19/10	63.22 ± 5.41	64.25 ± 5.44	CT_HF_ + SM(50 ml,qd)	CT_HF_	14 d	③⑥⑪
Wang and Wu, (2012)	<40%	40	40	24/16	27/13	54.3 ± 11.2	57.6 ± 12.1	CT_HF_ + SM(60 ml,qd)	CT_HF_	14 d	②③④⑪
Wang, (2014)	<45%	30	30	-		-		CT_HF_ + SM(100 ml,qd)	CT_HF_	28 d	②③⑨⑩⑪
Lin and Bao, (2013)	<45%	60	60	34/26	36/24	70.49 ± 8.42	76.06 ± 7.96	CT_HF_ + SM(50 ml,qd)	CT_HF_	14 d	①②⑧⑪
Chen and Zhou, (2010)	<45%	30	30	-		53 ± 10		CT_HF_ + SM(50 ml,qd)	CT_HF_	14 d	①③④⑪
Xu S. L. et al. (2018)	<50%	30	30	17/13	16/14	56.8 ± 4.52	55.8 ± 5.32	CT_HF_ + SM(40 ml,qd)	CT_HF_ (e.g., furosemide and metoprolol tartrate as appropriate; captopril 12.5 mg, bid)	14 d	③⑥⑦⑨
Lan and Wei, (2011)	<40%	30	30	17/13	16/14	57.5 ± 9.2		CT_HF_ + SM(50 ml,qd)	CT_HF_	-	②③⑦⑪
Zhang et al. (2021)	<50%	30	30	19/11	21/9	76.78 ± 6.8	65.90 ± 6.7	CT_HF_ + SM(60 ml,qd)	CT_HF_ (e.g., spironolaCTone and metoprolol tartrate as appropriate; sacubitril valsartan 50 mg/time, bid)	12 w	②③④⑤
Chen (2021)	<50%	43	43	26/17	27/16	66.73 ± 3.41	67.20 ± 3.52	CT_HF_ + SM(40 ml,qd)	CT_HF_ (e.g., bisoprolol as appropriate)	8 w	②③④⑤⑩
Jiang (2021)	<40%	32	32	23/9	18/14	62.08 ± 5.03	61.25 ± 5.19	CT_HF_ + SM(50 ml,qd)	CT_HF_ (e.g., furosemide tablets 20–40 mg/time, 2–3 times/d)	14 d	⑪
Hao et al. (2019)	<50%	23	23	13/10	14/9	56 ± 12	57 ± 13	CT_HF_ + XML(5 mg/kg,bid)	CT_HF_	7 d	②③⑧⑩
Wu et al. (2012)	≤45%	50	50	-		55.30 ± 2.2		CT_HF_ + XML(5 mg/kg,bid)	CT_HF_	5 d	③⑧
Tang et al. (2013)	≤45%	40	40	21/19	22/18	68 ± 4	67 ± 4	CT_HF_ + XML(5 mg/kg,bid)	CT_HF_	5 d	③⑨⑪
Feng et al. (2015)	≤45%	51	51	24/27	26/25	56.1 ± 8.2	54.7 ± 9.3	CT_HF_ + XML(8 mg/kg,bid)	CT_HF_	5 d	⑧
Liu, (2015)	<45%	28	32	18/10	20/12	72.07 ± 13.41	73.53 ± 10.65	CT_HF_ + XML(5 mg/kg,bid)	CT_HF_	5 d	①②③⑨
Wang, (2017)	≤45%	21	21	12/9	13/8	71.38 ± 9.23	71.81 ± 9.92	CT_HF_ + XML(5 mg/kg,bid)	CT_HF_	5 d	①②③⑨⑩
Hao, (2019)	<40%	39	41	17/22	20/21	73.6 ± 12.1	69.4 ± 10.5	CT_HF_ + XML(5 mg/kg,bid)	CT_HF_	5 d	①②③⑧⑪
Xue et al. (2019)	≤45%	50	50	29/21	27/23	63.8 ± 9.46	64.3 ± 7.78	CT_HF_ + XML(5 mg/kg,bid)	CT_HF_ (furosemide 20 mg, perindopril 4 mg, metoprolol 23.75 mg, isosorbide mononitrate 50 mg, 0.125 mg digoxin, qd)+placebo	5 d	①②③⑧
Yao et al. (2014)	<40%	45	46	51/40		M: 63.26 ± 5.64; F: 64.71 ± 6.78		CT_HF_ + XML(200 mg,qd)	CT_HF_	7 d	③④⑦⑧⑪
Xu E. W. et al. (2018)	<50%	44	44	24/20	19/25	66.1 ± 12.3	65.3 ± 11.6	CT_HF_ + XML(5 mg/kg,bid)	CT_HF_ (e.g., milrinone as appropriate)	7 d	②③⑧⑪⑪
Mao, (2019)	<45%	26	26	38/14		46.87 ± 5.41		CT_HF_ + XML(5 mg/kg,bid)	CT_HF_	10 d	①②③④⑨
Zhao et al. (2014)	≤45%	30	30	31/29		66(40`∼78)		CT_HF_ + XML(5 mg/kg∼10 mg/kg,bid)	CT_HF_	14 d	③
Peng et al. (2014)	<45%	56	56	32/24	30/26	71.1 ± 2.8	70.2 ± 2.6	CT_HF_ + XML(200 mg,bid)	CT_HF_	14 d	②③④⑤⑥⑦⑩
Liu et al. (2015)	<45%	60	76	43/17	48/28	44.2 ± 3.7	45.2 ± 3.5	CT_HF_ + XML(200 mg,bid)	CT_HF_	14 d	③④⑤⑨
Zhong, (2016)	<50%	50	50	33/17	29/21	64.99 ± 9.80	63.68 ± 9.48	CT_HF_ + XML(5 mg/kg∼10 mg/kg,bid)	CT_HF_ (e.g., enalapril tablets 5 mg, bid or valsartan capsules 80 mg, qd; hydrochlorothiazide tablets 25 mg, qd; spironolaCTone 20 mg, qd; isosorbide mononitrate tablets 40 mg, qd; metoprolol succinate sustained-release tablets 47.5 mg, qd; digoxin tablets 0.25 mg, qd)	14 d	①②③
Li J. F., (2018)	≤40%	36	36	21/15	23/13	77.92 ± 9.43	77.42 ± 9.55	CT_HF_ + XML(5 mg/kg,bid)	CT_HF_ (e.g., spironolaCTone tablets 20 mg, bid; digoxin tablets 0.125–0.25 mg, qd; benazepril hydrochloride tablets 5–10 mg, qd.)	14 d	②③⑦⑧⑪
Zhang, (2018)	≤40%	48	48	17/31	21/27	77.1 ± 7.96	75.98 ± 6.73	CT_HF_ + XML(5 mg/kg,bid)	CT_HF_ (e.g., digoxin 0.125–0.250 mg/time, qd; spironolaCTone tablets 20 mg/time, bid; benazepril 5–10 mg/time, qd; metoprolol as appropriate)	14 d	③④⑧⑪
Su et al. (2018)	<40%	42	41	17/25	18/23	61.1 ± 10.9	60.2 ± 11.8	CT_HF_ + XML(200 mg,qd)	CT_HF_ (e.g., nicorandil tablets 5 mg, tid)	15 d	②③④⑨
Li, (2020)	≤40%	62	62	34/28	37/25	69.27 ± 8.54	68.71 ± 8.29	CT_HF_ + XML(5 mg/kg,bid)	CT_HF_	14 d	②③④⑤⑪
Xi, (2020)	<45%	48	48	27/21	30/18	72.01 ± 10.41	71.65 ± 10.38	CT_HF_ + XML(5 mg/kg,bid)	CT_HF_	5 d	②③⑨
Wang et al. (2021)	<50%	30	30	17/13	18/12	65.43 ± 2.51	65.51 ± 2.13	CT_HF_ + XML(5 mg/kg,bid)	CT_HF_	15 d	③④⑨
Yuan and Du, (2012)	<50%	82	80	120/42		45∼98		CT_HF_ + YQFM(5.2 g,qd)	CT_HF_	10 d	②③⑪
Yu and Wang, (2012)	≤40%	30	30	15/15	16/14	66 ± 7	65 ± 6	CT_HF_ + YQFM(3.9 g,qd)	CT_HF_ (e.g., digoxin 0.125–0.25 mg, qd; furosemide 20 mg, qd; spironolaCTone 20 mg, qd; metoprolol tartrate, 12.5 mg, bid)	14 d	③⑧⑪
Zhao, (2015)	<50%	76	72	49/27	42/30	65.88 ± 11.23	64.35 ± 10.16	CT_HF_ + YQFM(3.9–5.2 g,qd)	CT_HF_	14 d	①③⑨⑪
Li, (2014)	<45%	41	39	23/18	21/18	67 ± 8	66 ± 7	CT_HF_ + YQFM(2.6 g,qd)	CT_HF_	14 d	②③④⑨
Guo, (2015)	<40%	60	60	72/48		69(60∼86)		CT_HF_ + YQFM(2.6 g,qd)	CT_HF_	14 d	①②③④⑨
Tao et al. (2020)	<50%	55	53	23/32	17/36	71.45 ± 12.66	74.45 ± 11.97	CT_HF_ + YQFM(2.6–5.2 g,qd)	CT_HF_	7∼14 d	③⑨⑩⑪
Kou (2021)	<50%	42	42	28/14	26/16	54.36 ± 4.71	54.28 ± 4.60	CT_HF_ + YQFM(3.9 g,qd)	CT_HF_ (e.g., digoxin tablets 0.25mg, qd)	14 d	③④⑤⑪
Qi, (2015)	<50%	48	48	61/35		65.61 ± 3.23		CT_HF_ + SF(20–100 ml,qd)	CT_HF_ + SGM(20–60 ml,bid)	12 w	③⑥⑦
Su et al. (2014)	<50%	30	30	19/11	21/9	15∼58		CT_HF_ + XML(5 mg/kg,bid)	CT_HF_ + HQ(30 ml,bid)	5 d	②③⑧⑪
Wang, (2019)	<50%	20	20	16/4	15/5	45∼74	40∼72	CT_HF_ + XML(5 mg/kg,bid)	CT_HF_ + SM(30 ml,bid)	5 d	①③⑧⑪

a Age is described in the form of mean ± standard deviation or mean (minimum ∼ maximum).^#^ “CT_HF_” refers to the conventional treatment for heart failure in the international heart failure guidelines, including angiotensin-converting enzyme inhibitor (such as captopril, enalapril), angiotensin receptor blocker (such as valsartan, irbesartan), β-blocker (such as metoprolol), mineralocorticoid receptor antagonists (spironolactone), cardiotonic drugs (such as digoxin), anticoagulant and antiplatelet drugs(such as aspirin), and so on. These drugs were the conventional doses recommended in the guidelines and needed to be adjusted according to the patient’s condition, especially β-blockers and diuretics. However, the treatment plan and principles of the “CT_HF_” of the test group and the control group were the same. LVEF, left ventricular ejection fraction; F, female; M, male; T, test group; C, control group; HQ, Huangqi injection; SF, Shenfu injection; SGM, Shengmai injection; SM, Shenmai injection; XML, Xinmailong injection; YQFM, Yiqifumai lyophilized injection; d, day(s); w, week(s). ① New York Heart Association cardiac functional classification efficiency; ② 6-min walking test; ③ left ventricular ejection fraction; ④ left ventricular end-diastolic diameter; ⑤ left ventricular end-systolic diameter; ⑥ cardiac output; ⑦ stroke volume; ⑧ brain natriuretic peptide; ⑨ N-terminal pro-brain natriuretic peptide; ⑩ Minnesota Living with Heart Failure Questionnaire; ⑪ adverse drug event.

### Risk of Bias Assessment

**FIGURE 3:**
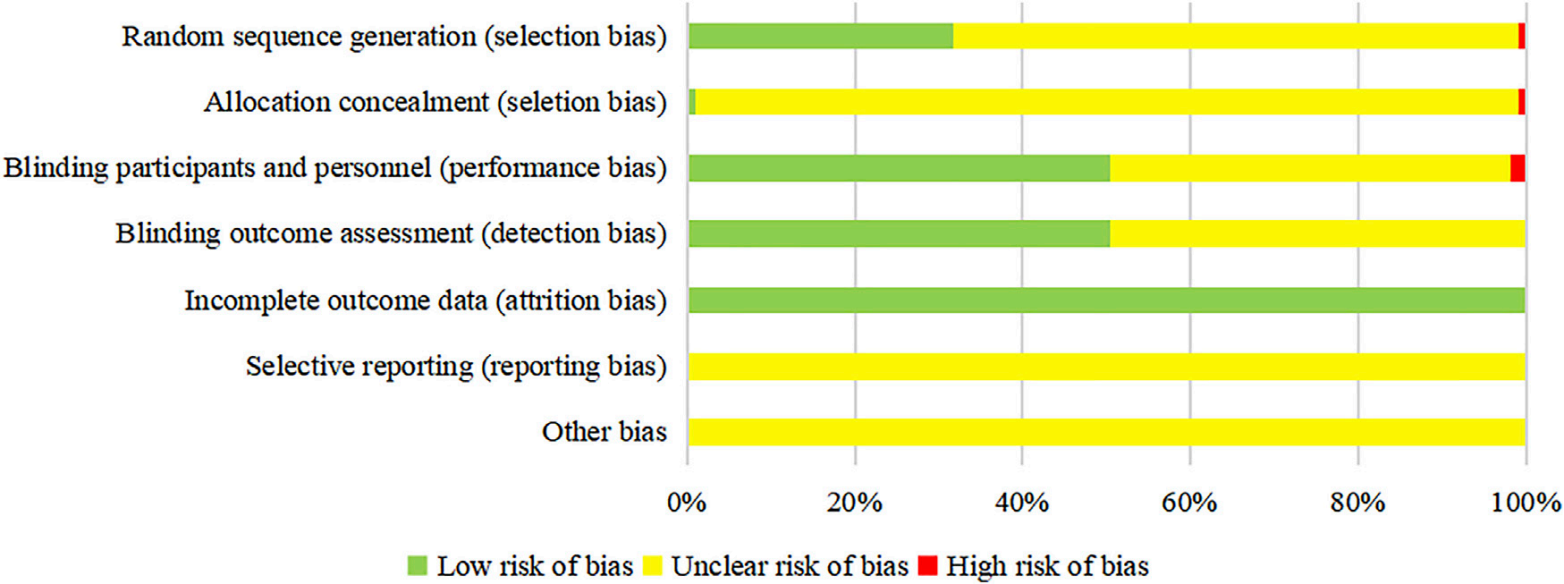
Risk of bias graph of the included RCTs. The vertical axis represents the risk of bias items, and the horizontal axis represents the percentage of the number of RCTs. For the outcome whose evaluation process is objective, the “unclear risk of bias” should be regarded as “low risk of bias”.

The results of the risk of bias assessment are as follows. ① Random sequence generation: 34 studies (Wang, 2003; Hu, et al., 2009; Zhang et al., 2014; Wu, 2016; Yao et al., 2016; Zhang et al., 2018; Qi and Qi, 2015; Wang et al., 2014; Song et al., 2006; Xu et al., 2014; Su, 2017; Wang et al., 2011; Pan, 2015; Yan et al., 2017; Li X. L., 2018; Pan, 2013; Kong, 2010; Zhai, 2013; Li et al., 2016; An and Xiao, 2019; He et al., 2020; He et al., 2018; Xu S. L. et al., 2018; Wang, 2017; Xue et al., 2019; Xu E. W. et al., 2018; Li J. F., 2018; Zhang, 2018; Li, 2020; Tao et al., 2020; Wang et al., 2021; Chen 2021; Jiang 2021; Kou 2021) were evaluated as low risk due to the use of a random number table, one study that was randomly grouped according to the order of visits (Ding et al., 2012) was assessed as high risk, and other studies did not describe specific randomization methods. ② Allocation concealment: one study (Xue et al., 2019) was evaluated as low risk due to the use of center allocation, and one study (Ding et al., 2012) was evaluated as high risk due to the failure of allocation concealment. ③ Blinding participants and personnel: two double-blind studies (Qi, 2015; Xue et al., 2019) were evaluated as low risk, two single-blind study (Wu and Duan, 2009; Mao, 2019) was high-risk, and other studies did not mention blinding. ④ Blinding outcome assessment: one study using center allocation (Xue et al., 2019) was evaluated as low-risk, and other studies did not mention whether the evaluators were blinded. ⑤ Incomplete outcome data: there was no missing data in all studies. ⑥ Selective report: all studies did not provide a protocol, so the risk of selective reporting was unclear. ⑦ Other bias: there was not enough information to assess whether there were other biases. The summary of the risk of bias of the included RCTs is shown in Figure 3.

### Results of the Network Meta-Analysis

#### NYHA Cardiac Functional Classification Efficiency

A total of 48 RCTs reported the NYHA cardiac functional classification efficiency, including six types of TCMIs and seven types of interventions (CT vs. CT + HQ (*n* = 4), CT vs. CT + SF (*n* = 28), CT vs. CT + SGM (*n* = 3), CT vs. CT + SM (*n* = 4), CT vs. CT + XML (*n* = 6), CT vs. CT + YQFM (*n* = 2), and CT + SM vs. CT + XML (*n* = 1)) (Table 1). The network diagram is shown in Figure 2. All TCMIs combined with CT were superior to CT alone, and the difference was statistically significant. The details are shown in Table 2: CT vs. CT + HQ (OR = 0.18, 95% CrI: 0.07–0.38), CT + SF vs. CT (OR = 0.25, 95% CrI: 0.19–0.33), CT vs. CT + SGM (OR = 0.36, 95% CrI: 0.16–0.72), CT vs. CT + SM (OR = 0.24, 95% CrI: 0.13–0.43), CT vs. CT + XML (OR = 0.18, 95% CrI: 0.11–0.31), and CT vs. CT + YQFM (OR = 0.38, 95% CrI: 0.19–0.71).

**TABLE 2:** Odds ratio/weight mean difference (95% credible interval) results of the network meta-analysis.

NYHA (%)^a^							
Outcomes	CT	CT + SF	CT + XML	CT + SM	CT + SGM	CT + HQ	CT + YQFM
CT	1						
CT + HQ	**0.18 (0.07, 0.38)**	1					
CT + SF	**0.25 (0.19, 0.33)**	1.42 (0.62, 3.47)	1				
CT + SGM	**0.36 (0.16, 0.72)**	2.10 (0.68, 6.15)	1.44 (0.60, 2.94)	1			
CT + SM	**0.24 (0.13, 0.43)**	1.35 (0.51, 3.83)	0.95 (0.49, 1.83)	0.67 (0.25, 1.97)	1		
CT + XML	**0.18 (0.11, 0.31)**	1.05 (0.41, 2.98)	0.74 (0.41, 1.30)	0.52 (0.22, 1.32)	0.79 (0.36, 1.66)	1	
CT + YQFM	**0.38 (0.19, 0.71)**	2.10 (0.76, 6.44)	1.48 (0.69, 2.95)	1.02 (0.41, 2.73)	1.57 (0.62, 3.85)	1.97 (0.86, 4.84)	1
**6WMT (m)**							
**Outcomes**	**CT**	**CT + SF**	**CT + XML**	**CT + SM**	**CT + SGM**	**CT + HQ**	**CT + YQFM**
CT	0						
CT + HQ	26.70 (−30.52, 82.86)	0					
CT + SF	−**42.29 (**−**62.99,** −**22.02)**	−**68.87 (**−**128.52,** −**8.05)**	0				
CT + SGM	-	-	-	0			
CT + SM	−**54.92 (**−**76.68,** −**32.32)**	−**81.61 (**−**141.43,** −**19.86)**	−12.74 (−42.26, 17.76)	-	0		
CT + XML	−**51.90 (**−**68.50,** −**35.82)**	−**78.73 (**−**133.10,** −**23.97)**	−9.57 (−35.99, 16.20)	-	3.08 (−25.68, 30.24)	0	
CT + YQFM	−**43.56 (**−**74.43,** −**13.17)**	−**70.41 (**−**134.81,** −**5.47)**	−1.31 (−38.00, 35.53)	-	11.38 (−27.27, 48.77)	8.24 (−26.55, 43.37)	0
**LVEF (%)**							
**Outcomes**	**CT**	**CT + SF**	**CT + XML**	**CT + SM**	**CT + SGM**	**CT + HQ**	**CT + YQFM**
CT	0						
CT + HQ	−**5.40 (**−**8.02,** −**2.82)**	0					
CT + SF	−**5.49 (**−**6.87,** −**4.11)**	−0.09 (−3.05, 2.87)	0				
CT + SGM	−**4.66 (**−**7.31,** −**1.98)**	0.74 (−2.95, 4.49)	0.83 (−2.09, 3.72)	0			
CT + SM	−**6.34 (**−**7.91,** −**4.72)**	−0.94 (−3.98, 2.09)	−0.86 (−2.94, 1.28)	−1.66 (−4.78, 1.41)	0		
CT + XML	−**5.35 (**−**6.92,** −**3.79)**	0.05 (−2.88, 3.00)	0.14 (−1.96, 2.21)	−0.69 (−3.77, 2.38)	0.99 (−1.22, 3.16)	0	
CT + YQFM	−**7.19 (**−**9.98,** −**4.45)**	−1.78 (−5.60, 2.02)	−1.69 (−4.82, 1.39)	−2.52 (−6.4, 1.32)	−0.85 (−4.1, 2.35)	−1.84 (−5.04, 1.35)	0
**LVEDD (mm)**							
**Outcomes**	**CT**	**CT + SF**	**CT + XML**	**CT + SM**	**CT + SGM**	**CT + HQ**	**CT + YQFM**
CT	0						
CT + HQ	2.05 (−2.46, 6.63)	0					
CT + SF	**3.17 (1.58, 4.76)**	1.14 (−3.70, 5.89)	0				
CT + SGM	**4.98 (0.54, 9.38)**	2.92 (−3.39, 9.28)	1.81 (−2.93, 6.46)	0			
CT + SM	**5.16 (3.17, 7.16)**	3.12 (−1.92, 8.06)	1.98 (−0.56, 4.54)	0.16 (−4.62, 5.06)	0		
CT + XML	**4.73 (2.70, 6.74)**	2.68 (−2.40, 7.63)	1.55 (−1.01, 4.10)	−0.25 (−5.12, 4.55)	−0.43 (−3.26, 2.38)	0	
CT + YQFM	1.96 (−1.39, 5.31)	−0.10 (−5.78, 5.58)	−1.22 (−4.92, 2.50)	−3.02 (−8.51, 2.52)	−3.18 (−7.05, 0.69)	−2.76 (−6.64, 1.18)	0
**LVESD (mm)**							
**Outcomes**	**CT**	**CT + SF**	**CT + XML**	**CT + SM**	**CT + SGM**	**CT + HQ**	**CT + YQFM**
CT	0						
CT + HQ	3.00 (−1.43, 7.51)	0					
CT + SF	2.93 (−0.53, 6.44)	−0.07 (−5.74, 5.57)	0				
CT + SGM	3.10 (−1.24, 7.47)	0.12 (−6.20, 6.30)	0.18 (−5.44, 5.75)	0			
CT + SM	**5.62 (2.71, 8.51)**	2.64 (−2.79, 7.87)	2.70 (−1.87, 7.18)	2.51 (−2.71, 7.75)	0		
CT + XML	2.80 (−0.34, 6.05)	−0.18 (−5.75, 5.24)	−0.13 (−4.91, 4.66)	−0.30 (−5.66, 5.12)	−2.81 (−7.11, 1.56)	0	
CT + YQFM	5.01 (−0.73, 10.68)	2.00 (−5.39, 9.17)	2.08 (−4.72, 8.75)	1.90 (−5.29, 9.04)	−0.62 (−7.03, 5.87)	2.21 (−4.38, 8.67)	0

**CO (L/min)**							
**Outcomes**	**CT**	**CT + SF**	**CT + XML**	**CT + SM**	**CT + SGM**	**CT + HQ**	**CT + YQFM**
CT	0						
CT + HQ	−**0.86 (**−**1.44,** −**0.28)**	0					
CT + SF	−**0.66 (**−**0.98,** −**0.35)**	0.20 (−0.47, 0.86)	0				
CT + SGM	−**0.77 (**−**1.13,** −**0.42)**	0.09 (−0.60, 0.76)	−0.12 (−0.55, 0.33)	0			
CT + SM	−**0.86 (**−**1.37,** −**0.37)**	0.00 (−0.78, 0.76)	−0.20 (−0.79, 0.39)	−0.09 (−0.71, 0.53)	0		
CT + XML	−0.30 (−1.22, 0.61)	0.56 (−0.54, 1.64)	0.35 (−0.61, 1.34)	0.47 (−0.52, 1.46)	0.56 (−0.47, 1.61)	0	
CT + YQFM	-	-	-	-	-	-	0
							
**SV (ml) Outcomes**	**CT**	**CT + SF**	**CT + XML**	**CT + SM**	**CT + SGM**	**CT + HQ**	**CT + YQFM**
CT	0						
CT + HQ	−**10.88 (**−**18.61,** −**3.11)**	0					
CT + SF	−**9.07 (**−**14.18,** −**3.78)**	1.86 (−7.56, 11.16)	0				
CT + SGM	−4.32 (−10.47, 1.79)	6.56 (−3.36, 16.49)	4.73 (−2.63, 12.02)	0			
CT + SM	−5.68 (−11.47, 0.19)	5.21 (−4.54, 14.92)	3.38 (−4.36, 11.13)	−1.34 (−9.75, 7.17)	0		
CT + XML	−**8.23 (**−**16.18,** −**0.29)**	2.64 (−8.49, 13.69)	0.83 (−8.75, 10.17)	−3.93 (−14.03, 6.09)	−2.58 (−12.45, 7.23)	0	
CT + YQFM	-	-	-	-	-	-	0
**BNP (pg/ml)**							
**Outcomes**	**CT**	**CT + SF**	**CT + XML**	**CT + SM**	**CT + SGM**	**CT + HQ**	**CT + YQFM**
CT	0						
CT + HQ	50.08 (−126.18, 227.22)	0					
CT + SF	**149.34 (61.01, 242.79)**	99.36 (−98.09, 303.50)	0				
CT + SGM	21.55 (−280.52, 321.54)	−29.18 (−378.93, 325.07)	−127.22 (−446.17, 181.95)	0			
CT + SM	128.46 (−8.04, 274.35)	77.38 (−140.16, 307.91)	−20.82 (−185.85, 147.04)	105.90 (−220.15, 449.65)	0		
CT + XML	**194.42 (95.37, 293.16)**	144.43 (−40.64, 332.77)	44.90 (−92.47, 175.44)	173.12 (−143.88, 492.27)	65.88 (−96.79, 220.38)	0	
CT + YQFM	-	-	-	-	-	-	0
**NT-proBNP (pg/ml)**							
**Outcomes**	**CT**	**CT + SF**	**CT + XML**	**CT + SM**	**CT + SGM**	**CT + HQ**	**CT + YQFM**
CT	0						
CT + HQ	-	0					
CT + SF	**285.71 (88.95, 499.14)**	-	0				
CT + SGM	328.58 (−1,004.61, 1678.03)	-	41.02 (−1,315.93, 1401.68)	0			
CT + SM	**443.31 (181.32, 708.19)**	-	158.26 (−184.72, 486.14)	115.96 (−1,257.24, 1,477.16)	0		
CT + XML	**587.91 (329.09, 860.28)**	-	301.63 (−34.74, 637.27)	258.49 (−1,115.62, 1,626.83)	144.83 (−221.44, 522.33)	0	
CT + YQFM	229.94 (−146.66, 601.07)	-	−55.22 (−494.14, 357.65)	−101.38 (−1,505.40, 1,286.82)	−213.87 (−675.22, 240.66)	−358.39 (−833.47, 95.35)	0
**MLHFQ (point)**							
**Outcomes**	**CT**	**CT + SF**	**CT + XML**	**CT + SM**	**CT + SGM**	**CT + HQ**	**CT + YQFM**
CT	0						
CT + HQ	6.47 (−5.29, 18.35)	0					
CT + SF	**7.32 (1.13, 15.22)**	0.81 (−12.06, 15.43)	0				
CT + SGM	-	-	-	0			
CT + SM	4.57 (−3.47, 12.47)	−1.92 (−16.43, 12.24)	−2.74 (−14.35, 7.06)	-	0		
CT + XML	**7.00 (0.82, 13.37)**	0.52 (−12.93, 14.00)	−0.34 (−10.37, 8.38)	-	2.45 (−7.52, 12.77)	0	
CT + YQFM	2.84 (−8.54, 14.14)	−3.65 (−20.11, 12.67)	−4.47 (−18.52, 8.14)	-	−1.72 (−15.67, 12.23)	−4.19 (−17.24, 8.76)	0

The bolded values indicate statistical significance. NYHA, New York Heart Association cardiac functional classification efficiency; 6WMT, 6-min walking test; LVEF, left ventricular ejection fraction; LVEDD, left ventricular end-diastolic diameter; LVESD, left ventricular end-systolic diameter; CO, cardiac output; SV, stroke volume; BNP, brain natriuretic peptide; NT-proBNP, N-terminal pro-brain natriuretic peptide; MLHFQ, Minnesota Living with Heart Failure Questionnaire; CT, conventional treatment; HQ, Huangqi injection; SF, Shenfu injection; SGM, Shengmai injection; SM, Shenmai injection; XML, Xinmailong injection; YQFM, Yiqifumai lyophilized injection.

a Indicates that the value is odds ratio.

**FIGURE 4:**
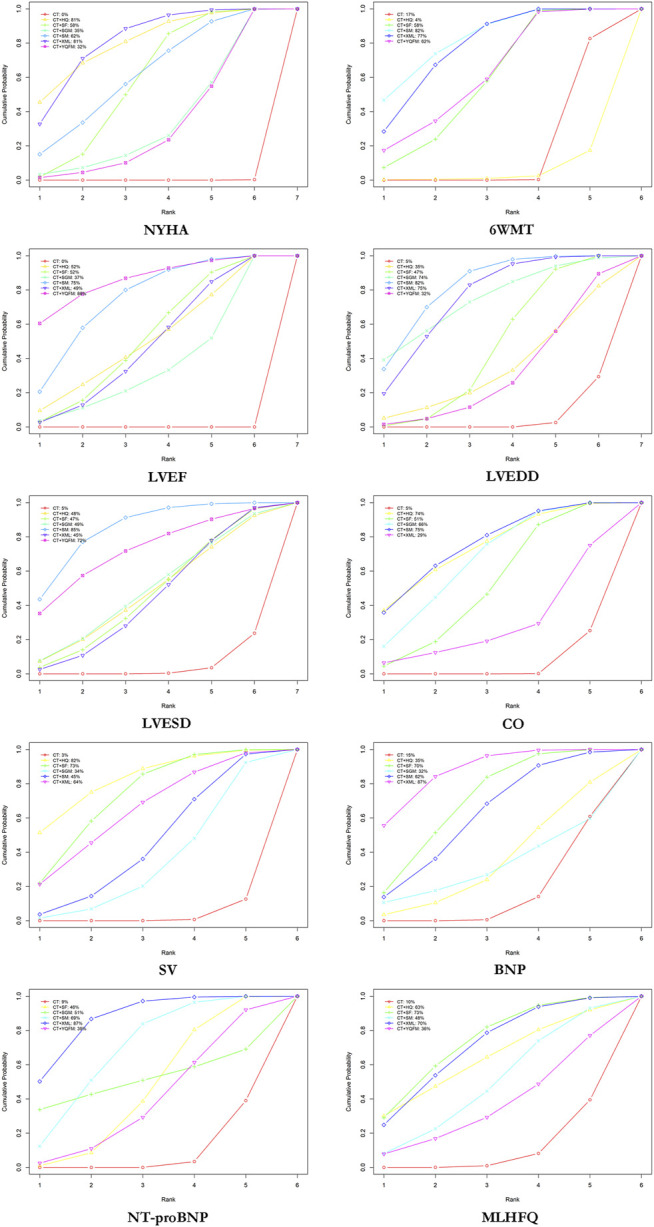
Surface under the cumulative ranking curve (SUCRA) plots for different outcomes. The vertical axis represents cumulative probabilities and the horizontal axis represents rank. NYHA, New York Heart Association cardiac functional classification efficiency; 6WMT, 6-min walking test; LVEF, left ventricular ejection fraction; LVEDD, left ventricular end-diastolic diameter; LVESD, left ventricular end-systolic diameter; CO, cardiac output; SV, stroke volume; BNP, brain natriuretic peptide; NT-proBNP, N-terminal pro-brain natriuretic peptide; MLHFQ, Minnesota Living with Heart Failure Questionnaire; CT, conventional treatment; HQ, Huangqi injection; SF, Shenfu injection; SGM, Shengmai injection; SM, Shenmai injection; XML, Xinmailong injection; YQFM, Yiqifumai lyophilized injection.

According to SUCRA probability results (Figure 4; Table 3), CT + XML was the most likely to be the best intervention for improving the NYHA cardiac functional classification efficiency. The ranking of seven interventions is as follows: CT + XML (81.36%) > CT + HQ (80.87%) > CT + SM (62.19%) > CT + SF (58.46%) > CT + SGM (34.68%) > CT + YQFM (32.39%) > CT (0.05%).

**TABLE 3:** Results of the surface under the cumulative ranking curve (SUCRA) (%).

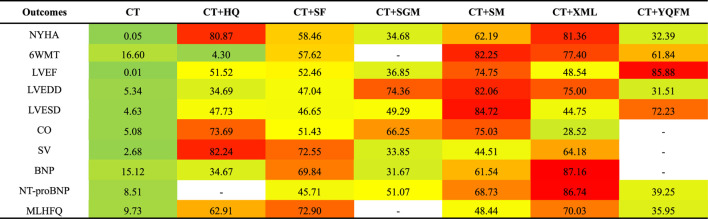

Outcomes	CT	CT + HQ	CT + SF	CT + SGM	CT + SM	CT + XML	CT + YQFM
NYHA	0.05	80.87	58.46	34.68	62.19	81.36	32.39
6WMT	16.60	4.30	57.62	-	82.25	77.40	61.84
LVEF	0.01	51.52	52.46	36.85	74.75	48.54	85.88
LVEDD	5.34	34.69	47.04	74.36	82.06	75.00	31.51
LVESD	4.63	47.73	46.65	49.29	84.72	44.75	72.23
CO	5.08	73.69	51.43	66.25	75.03	28.52	-
SV	2.68	82.24	72.55	33.85	44.51	64.18	-
BNP	15.12	34.67	69.84	31.67	61.54	87.16	-
NT-proBNP	8.51	-	45.71	51.07	68.73	86.74	39.25
MLHFQ	9.73	62.91	72.90	-	48.44	70.03	35.95

The redder the color, the greater the SUCRA, and the greater the probability of becoming the best intervention. The greener the color, the smaller the SUCRA, and the smaller the probability of becoming the best intervention. NYHA, New York Heart Association cardiac functional classification efficiency; 6WMT, 6-min walking test; LVEF, left ventricular ejection fraction; LVEDD, left ventricular end-diastolic diameter; LVESD, left ventricular end-systolic diameter; CO, cardiac output; SV, stroke volume; BNP, brain natriuretic peptide; NT-proBNP, N-terminal pro-brain natriuretic peptide; MLHFQ, Minnesota Living with Heart failure questionnaire; CT, conventional treatment; HQ, Huangqi injection; SF, Shenfu injection; SGM, Shengmai injection; SM, Shenmai injection; XML, Xinmailong injection; YQFM, Yiqifumai lyophilized injection.

#### 6MWT

A total of 31 RCTs reported 6MWT, including five types of TCMIs and six types of interventions (CT vs. CT + SF (*n* = 7), CT vs. CT + SM (*n* = 7), CT vs. CT + XML (*n* = 13), CT vs. CT + YQFM (*n* = 3), and CT + SM vs. CT + XML (*n* = 1)) (Table 1). The network diagram is shown in Figure 2. Apart from HQ, other TCMIs combined with CT were superior to CT alone, and the difference was statistically significant. The details are shown in Table 2: CT vs. CT + SF (WMD = −42.29 m, 95% CrI: −62.99∼−22.02 m), CT vs. CT + SM (WMD = −54.92 m, 95% CrI: −76.68∼−32.32 m), CT vs. CT + XML (WMD = −51.9 m 0, 95% CrI: −68.50∼−35.82 m), and CT vs. CT + YQFM (WMD = −43.56 m, 95% CrI: −74.43∼−13.17 m). In addition, there were statistically significant differences between CT + HQ and CT combined with other injections. As shown in Table 2: CT + HQ vs. CT + SF (WMD = −68.87 m, 95% CrI: −128.52∼−8.05 m), CT + HQ vs. CT + SM (WMD = −81.61 m, 95% CrI: −141.43∼−19.86 m), CT + HQ vs. CT + XML (WMD = −78.73 m, 95% CrI: −133.10∼−23.97 m), and CT + HQ vs. CT + YQFM (WMD = −70.41 m, 95% CrI: −134.81∼−5.47 m) had more advantages in improving 6MWT.

According to SUCRA probability results (Figure 4; Table 3), CT + SM was the most likely to become the best intervention for improving 6MWT. The ranking of six interventions is as follows: CT + SM (82.25%) > CT + XML (77.40%) > CT + YQFM (61.84%) > CT + SF (57.62%) > CT (16.60%) > CT + HQ (4.30%).

#### LVEF

A total of 91 RCTs reported LVEF, including six types of TCMIs and seven types of interventions (CT vs. CT + HQ (*n* = 7), CT vs. CT + SF (*n* = 28), CT vs. CT + SGM (*n* = 7), CT vs. CT + SM (*n* = 19), CT vs. CT + XML (*n* = 20), CT vs. CT + YQFM (*n* = 7), CT + HQ vs. CT + XML (*n* = 1), CT + SM vs. CT + XML (*n* = 1), and CT + SGM vs. CT + SF (*n* = 1)) (Table 1). The network diagram is shown in Figure 2. All TCMIs combined with CT were superior to CT alone, and the difference was statistically significant. The details are shown in Table 2.

According to SUCRA probability results (Figure 4; Table 3), CT + YQFM was the most likely to become the best intervention for improving LVEF. The ranking of seven interventions is as follows: CT + YQFM (85.88%) > CT + SM (74.75%) > CT + SF (52.46%) > CT + HQ (51.52%) > CT + XML (48.54%) > CT + SGM (36.85%) > CT (0.01%).

#### LVEDD

A total of 38 RCTs reported LVEDD, including six types of TCMIs and seven types of interventions (CT vs. CT + HQ (*n* = 2), CT vs. CT + SF (*n* = 15), CT vs. CT + SGM (*n* = 2), CT vs. CT + SM (*n* = 8), CT vs. CT + XML (*n* = 8), and CT vs. CT + YQFM (*n* = 3)) (Table 1). The network diagram is shown in Figure 2. Apart from HQ and YQFM, other TCMIs combined with CT were superior to CT alone, and the difference was statistically significant. In addition, there were also statistically significant differences between different injections, such as CT + SF vs. CT + SGM, CT + SF vs. CT + SM, CT + SF vs. CT + XML, CT + SF vs. CT + YQFM, and CT + XML vs. CT + YQFM. The details are shown in Table 2.

According to SUCRA probability results (Figure 4; Table 3), CT + SM was the most likely to become the best intervention for improving LVEDD. The ranking of seven interventions is as follows: CT + SM (82.06%) > CT + XML (75.00%) > CT + SGM (74.36%) > CT + SF (47.04%) > CT + HQ (34.69%) > CT + YQFM (31.51%) > CT (5.34%).

#### LVESD

A total of 15 RCTs reported LVESD, including six types of TCMIs and seven types of interventions (CT vs. CT + HQ (*n* = 2), CT vs. CT + SF (*n* = 3), CT vs. CT + SGM (*n* = 2), CT vs. CT + SM (*n* = 4), CT vs. CT + XML (*n* = 3), and CT vs. CT + YQFM (*n* = 1)) (Table 1). The network diagram is shown in Figure 2. Table 2 shows that only XML combined with CT were superior to CT alone, and the difference was statistically significant.

According to SUCRA probability results (Figure 4; Table 3), CT + SM was the most likely to become the best intervention for improving LVESD. The ranking of six interventions is as follows: CT + SM (84.72%) > CT + YQFM (72.23%) > CT + SGM (49.29%) > CT + HQ (47.73%) > CT + SF (46.65%) > CT + XML (44.75%) > CT (4.63%).

#### CO

A total of 19 RCTs reported CO, including five types of TCMIs and six types of interventions (CT vs. CT + HQ (*n* = 2), CT vs. CT + SF (*n* = 7), CT vs. CT + SGM (*n* = 5), CT vs. CT + SM (*n* = 3), CT vs. CT + XML (*n* = 1), and CT + SF vs. CT + SGM (*n* = 1)) (Table 1). The network diagram is shown in Figure 2. Apart from XML, other TCMIs combined with CT were superior to CT alone, and the difference was statistically significant. The details are shown in Table 2.

According to SUCRA probability results (Figure 4; Table 3), CT + SM was the most likely to become the best intervention for improving CO. The ranking of six interventions is as follows: CT + SM (75.03%) > CT + HQ (73.69%) > CT + SGM (66.25%) > CT + SF (51.43%) > CT + XML (28.52%) > CT (5.08%).

#### SV

A total of 24 RCTs reported SV, including five types of TCMIs and six types of interventions (CT vs. CT + HQ (*n* = 3), CT vs. CT + SF (*n* = 7), CT vs. CT + SGM (*n* = 4), CT vs. CT + SM (*n* = 6), CT vs. CT + XML (*n* = 3), and CT + SF vs. CT + SGM (*n* = 1)) (Table 1). The network diagram is shown in Figure 2. Table 2 shows that only HQ, SF, and YQFM combined with CT were superior to CT alone, and the difference was statistically significant.

According to SUCRA probability results (Figure 4; Table 3), CT + HQ was the most likely to become the best intervention for improving SV. The ranking of six interventions is as follows: CT + HQ (82.24%) > CT + SF (72.55%) > CT + XML (64.18%) > CT + SM (44.51%) > CT + SGM (33.85%) > CT (2.68%).

#### BNP

A total of 32 RCTs reported BNP, including five types of TCMIs and six types of interventions (CT vs. CT + HQ (*n* = 2), CT vs. CT + SF (*n* = 14), CT vs. CT + SGM (*n* = 1), CT vs. CT + SM (*n* = 4), CT vs. CT + XML (*n* = 9), CT + HQ vs. CT + XML (*n* = 1), and CT + SM vs. CT + XML (*n* = 1)) (Table 1). The network diagram is shown in Figure 2. Table 2 shows that only SF and XML combined with CT were superior to CT alone, and the difference was statistically significant.

According to SUCRA probability results (Figure 4; Table 3), CT + XML (87.11%) was the most likely to become the best intervention for improving BNP. The ranking of six interventions is as follows: CT + XML (87.16%) > CT + SF (69.84%) > CT + SM (61.54%) > CT + HQ (34.67%) > CT + SGM (31.67%) > CT (15.12%).

#### NT-proBNP

A total of 31 RCTs reported NT-proBNP, including five types of TCMIs and six types of interventions (CT vs. CT + SF (*n* = 11), CT vs. CT + SGM (*n* = 1), CT vs. CT + SM (*n* = 7), CT vs. CT + XML (*n* = 8), and CT vs. CT + YQFM (*n* = 4)) (Table 1). The network diagram is shown in Figure 2. Table 2 shows that only SF, SM, and XML combined with CT were superior to CT alone, and the difference was statistically significant.

According to SUCRA probability results (Figure 4; Table 3), CT + XML was the most likely to become the best intervention for improving NT-proBNP. The ranking of six interventions is as follows: CT + XML (86.74%) > CT + SM (68.73%) > CT + SGM (51.07%) > CT + SF (45.71%) > CT + YQFM (39.25%) > CT (8.51%).

#### MLHFQ

A total of 11 RCTs reported MLHFQ, including five types of TCMIs and six types of interventions (CT vs. CT + HQ (*n* = 1), CT vs. CT + SF (*n* = 3), CT vs. CT + SM (*n* = 2), CT vs. CT + XML (*n* = 4), and CT vs. CT + YQFM (*n* = 1)) (Table 1). The network diagram is shown in Figure 2. Table 2 shows that only SF and XML combined with CT were superior to CT alone, and the difference was statistically significant.

According to SUCRA probability results (Figure 4; Table 3), CT + SF was the most likely to become the best intervention for improving MLHFQ. The ranking of six interventions is as follows: CT + SF (72.90%) > CT + XML (70.03%) > CT + HQ (62.91%) > CT + SM (48.88%) > CT + YQFM (35.95%) > CT (9.73%).

### Safety

Forty RCTs focused on adverse drug reactions (ADRs), mainly including dizziness and headache, gastrointestinal discomfort, skin allergic reactions, lowered blood pressure, and abnormal heart rhythm. Considering that the criteria for ADR in each RCT were not completely consistent, a descriptive analysis was carried out. Given the small sample size, it is not yet possible to prove that the difference between the two groups is statistically significant. Details are shown in Tables 1, 4.

**TABLE 4:** Summary of adverse drug events.

Type of interventions	Number of RCTs	Groups	Total sample size	Incidence	Detailed ADR events (number of cases)
CT + HQ vs. CT	0	CT + HQ	0	-	-
		CT	0	-	-
CT + SF vs. CT	11	CT + SF	439	1.82%	Dry mouth (4), headache (2), swelling at the injection site (1), and pain at the injection site (1)
		CT	433	0%	None
CT + SGM vs. CT	4	CT + SGM	165	1.81%	Dizziness and nausea (1), and unknown condition (2)
		CT	163	0%	None
CT + SM vs. CT	11	CT + SM	465	9.46%	Dizziness and headache (14), nausea and vomiting (13), fatigue (7), hypotension (8), ventricular premature beats (2)
		CT	465	8.82%	Dizziness and headache (9), nausea and vomiting (11), fatigue (9), hypotension (10), ventricular premature beats (2)
CT + XML vs. CT	7	CT + XML	314	6.62%	Gastrointestinal discomfort (3), dry mouth and nausea (3), headache (4), hypotension (1), skin pruritus (4), tachycardia (1), and palpitations (3)
		CT	317	3.79%	Headache (4), dizziness (1), skin pruritus (3), itching at the infusion site (1), mild skin redness (1), tachycardia (1), and palpitations (1)
CT + YQFM vs. CT	5	CT + YQFM	285	1.05%	Nausea and vomiting (2), elevated transaminases (1)
		CT	277	2.53%	Nausea and vomiting (3), abdominal pain (1), palpitations (1), elevated transaminases (2)
CT + XML vs. CT + HQ	1	CT + XML	30	6.67%	Skin pruritus (2)
		CT + HQ	30	0%	None
CT + XML vs. CT + SM	1	CT + XML	20	5.00%	Skin pruritus (1)
		CT + SM	20	0%	None

RCTs, randomized controlled trials; CT, conventional treatment; HQ, Huangqi injection; SF, Shenfu injection; SGM, Shengmai injection; SM, Shenmai injection; XML, Xinmailong injection; YQFM, Yiqifumai lyophilized injection.

### Multi-Dimensional Efficacy Analysis

**FIGURE 5:**
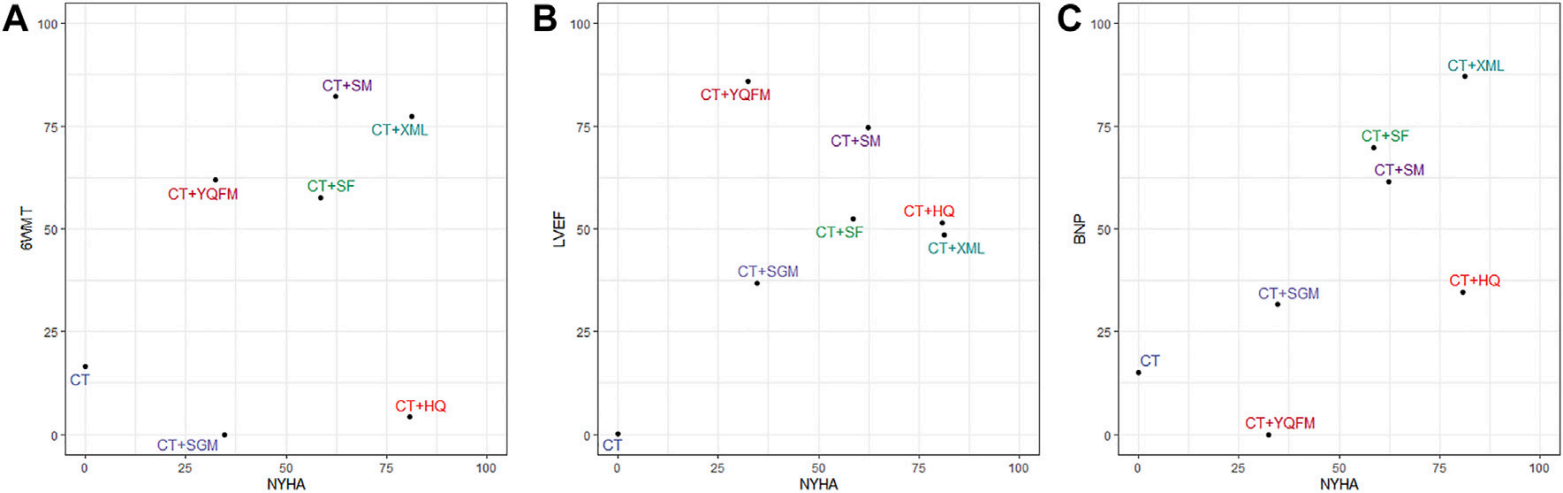
Multi-dimensional efficacy analysis results. **(A)** NYHA and 6WMT; **(B)** NYHA and LVEF; **(C)** NYHA and BNP. Interventions located in the upper right corner indicate optimal therapy for two different outcomes. NYHA, New York Heart Association cardiac functional classification efficiency; 6WMT, 6-min walking test; LVEF, left ventricular ejection fraction; BNP, brain natriuretic peptide; CT, conventional treatment; HQ, Huangqi injection; SF, Shenfu injection; SGM, Shengmai injection; SM, Shenmai injection; XML, Xinmailong injection; YQFM, Yiqifumai lyophilized injection.

This multi-dimensional efficacy analysis method integrated the results of multiple outcomes to obtain the optimal intervention. The results based on currently available clinical data indicate that, in terms of the primary outcomes (NYHA cardiac functional classification efficiency and 6WMT), CT + XML and CT + SM may be the best two treatments, followed by CT + SF, CT + YQFM, CT + HQ, and CT + SGM (Figure 5A). In terms of NYHA cardiac functional classification efficiency and the most common cardiac ultrasound index LVEF, CT + SM, CT + HQ, CT + XML, and CT + YQFM may all be potentially optimal choices (Figure 5B). Also, in terms of NYHA cardiac functional classification efficiency and the most common laboratory test index BNP, CT + XML may be the optimal choice (Figure 5C).

### Publication Bias

**FIGURE 6:**
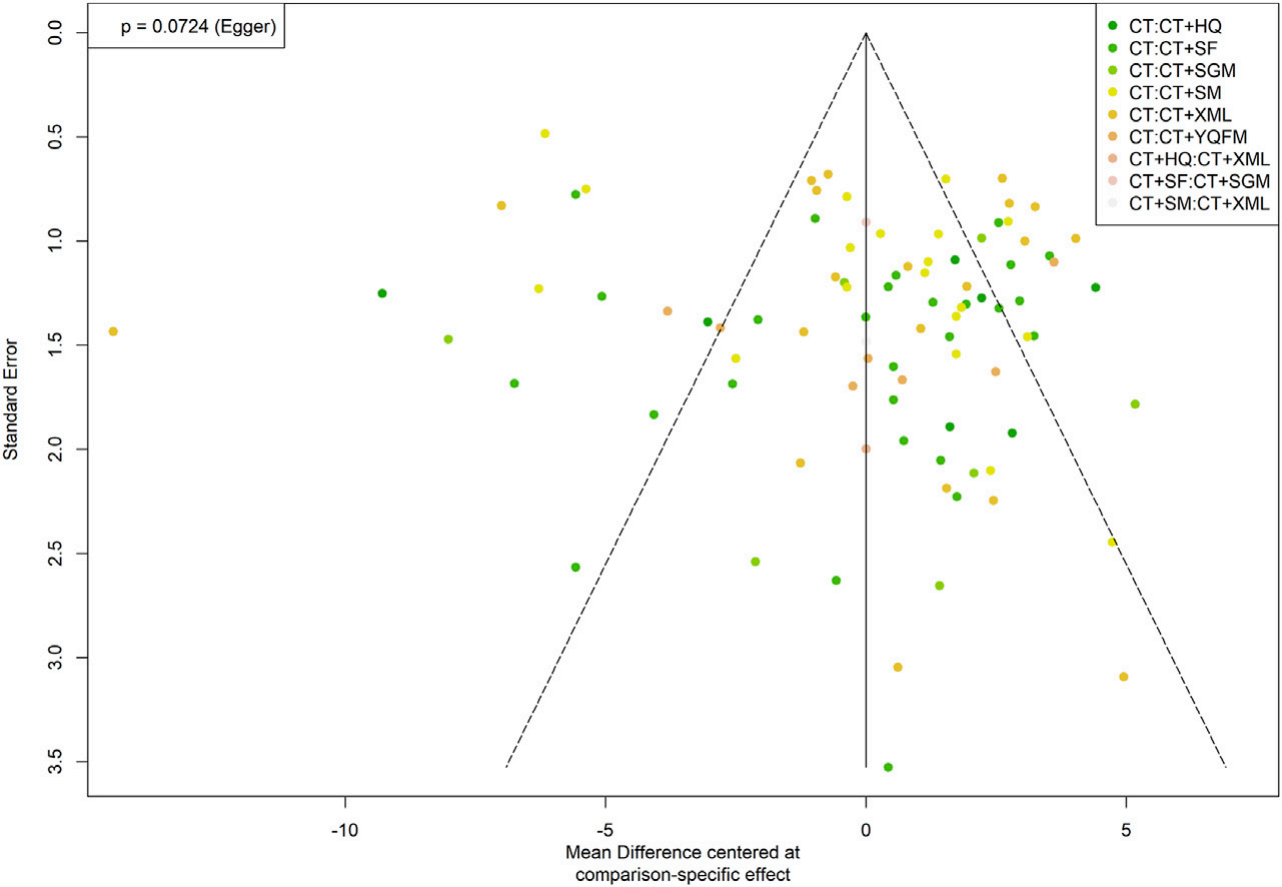
Funnel plot of left ventricular ejection fraction. CT, conventional treatment; HQ, Huangqi injection; SF, Shenfu injection; SGM, Shengmai injection; SM, Shenmai injection; XML, Xinmailong injection; YQFM, Yiqifumai lyophilized injection.

The comparison-adjusted funnel plots for each outcome were plotted, the result of LVEF with the biggest sample size is shown in Figure 6. The points in the funnel chart were asymmetric based on the position of the centerline, and the scatter was found to be symmetrical along the null line to the left and right, indicating that there was no small sample effect and publication bias. Funnel plots of all outcomes were shown in Supplementary File S3.

### Consistency Tests

**FIGURE 7:**
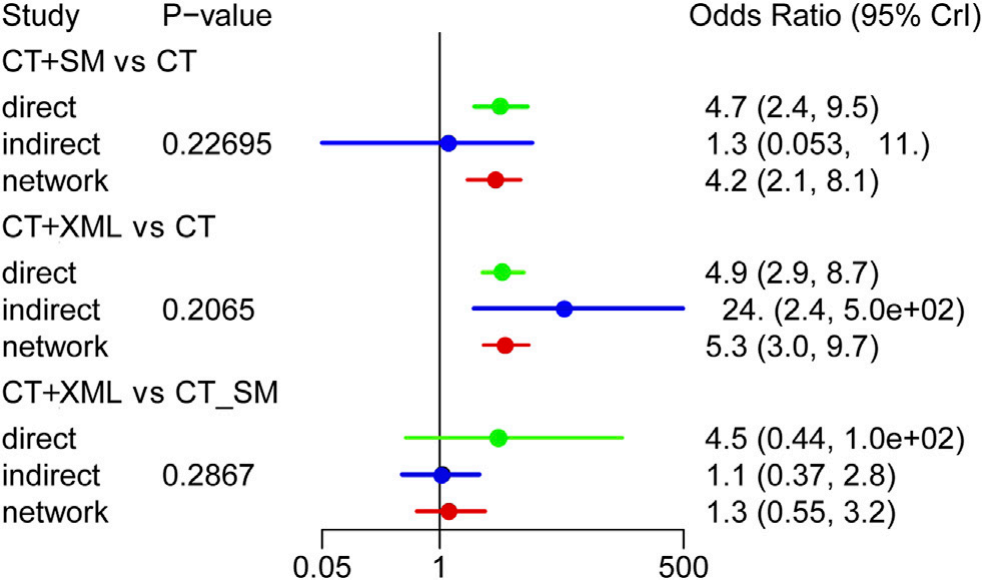
Consistency test for the New York Heart Association cardiac functional classification efficiency. CT, conventional treatment; SM, Shenmai injection; XML, Xinmailong injection.

As shown in Figure 2, there are some closed loops in the NYHA cardiac function classification efficiency, 6WMT, LVEF, CO, SV, and BNP network diagram. Therefore, the node-splitting analysis was used to evaluate the inconsistency of the model. Figure 7 shows that there was no statistically significant inconsistency in the closed-loop of NYHA cardiac functional classification efficiency (*p* > 0.05), indicating that the results on NYHA cardiac functional classification efficiency were reliable. The detailed results of the consistency test for all outcomes are shown in Supplementary File S4.

## Discussion

### Scientific Significance

At present, the highly contagious novel coronavirus (severe acute respiratory syndrome coronavirus 2, SARS-CoV2) has ravaged more than 200 countries around the world, triggering a global pandemic of coronavirus disease 2019 (COVID-19). Due to the unclear pathogenesis of COVID-19, there is no specific medicine in western medicine, which has caused a large number of deaths worldwide. Globally, as of 10:37am CEST, August 9, 2021, there have been 202,296,216 confirmed cases of COVID-19, including 4,288,134 deaths, reported to World Health Organization ( *https://covid19.who.int/* ). During the pandemic, the Chinese government proposes that TCM combined with western medicine can be used in treating COVID-19. TCM has been playing an irreplaceable role, significantly improving the quality of life of the affected population and reducing the mortality rate (Zhao et al., 2021). It is worth mentioning that TCM is not only widely used in China, but also transported to and served in many epidemic areas around the world. The unique advantages and clinical value of TCM have received increasing attention from all over the world.

TCM has accumulated thousands of years of clinical experience in the treatment of many diseases. Among them, HF is one of the dominant diseases of TCM. In 2018, the “*Guidelines for diagnosis and treatment of heart failure in China 2018*” (Heart Failure Group of Chinses Society of Cardiology of Chinses Medical Association et al., 2018) officially listed TCM as a recommendation for the treatment of HF. At present, the combination of traditional Chinese and Western medicine has become the basic model for the treatment of HF in China. Epidemiological investigations show that the common pathogenesis includes hypertension, cardiomyopathy, rheumatic heart disease, etc., of which ischemic heart disease is a major contributor to HF in Asia (Sakata and Shimokawa, 2013). According to the theory of TCM, the main pathogenesis of HF is “Qi deficiency (the lack of energy in the body in modern medicine, usually manifested as fatigue, spontaneous sweating, etc.) and blood stasis (an abnormal hemodynamic state in which blood tends to clot in modern medicine)”. The raw materials for the preparation of TCMIs are derived from total extracts, active parts or monomeric active ingredients extracted from traditional herbal medicines or traditional animal medicines. Through modern technology, further liquid preparation, purification, distillation, sterilization, etc. are accurately implemented to complete the entire preparation process.

The TCMIs included in this study all have the effect of nourishing Qi and blood. And a large number of articles and reviews have explored the protective effects of the active ingredients of these TCMIs on cardiovascular diseases in terms of molecular mechanisms, such as Huang-Qi (*Radix Astragali*) (Su et al., 2021) in HQ, the combination of Ren-Shen (*Radix Ginseng*) and Fu-Zi (processed) (*Radix Aconiti Lateralis Preparata*) (Tan et al., 2018; Li L. et al., 2020) in SF, the combination of Hong-Shen (*Radix Ginseng Rubra*) and Mai-Dong (*Radix Ophiopogonis*) (Wang et al., 2019) in SM and YQFM, Wu-Wei-Zi (*Fructus Schisandrae*) (Nasser et al., 2020) in SGM, and American cockroach (*Periplaneta Americana Linnaeus*) (Qi et al., 2017; Lin S. S. et al., 2019) in XML. Due to the available active ingredients, rich and diverse targets, and complex regulatory mechanisms, TCMIs have considerable development potential. This study clarified the clinical advantages of different TCMIs by performing an NMA. On the one hand, it will provide a reference for the rational use of TCMIs. On the other hand, molecular studies based on the clinical characteristics of TCMIs can provide ideas and clues for the development of new drugs.

### Summary of Main Findings

This NMA incorporated 107 RCTs on the efficacy and safety of TCM combined with CT in the treatment of HFrEF, including 9,073 patients and 6 TCMs. Among them, TCMIs include HQ, SF, SGM, SM, XML, and YQFM. In terms of clinical efficacy, the combination of CT + XML may be the most effective in improving NYHA cardiac functional classification efficiency, BNP, and NT-proBNP; the combination of CT + SM may be the most effective in improving 6MWT, LVEDD, LVESD, and CO; the combination of CT + YQFM may be the most effective in improving LVEF; the combination of CT + HQ may be the most effective in improving SV; the combination of CT + SF may be the most effective in improving MLHFQ. The results based on currently available clinical data show that the ranking results of TCMIs in various outcomes are inconsistent. Each species has its own advantages. Among them, the overall evaluation of CT + XML and CT + SM is relatively good. In terms of safety, CT + TCMIs may cause additional ADR events. However, there was no significant difference compared with the CT. The recommendation of clinical drugs requires a multi-dimensional evaluation system. In this study, NYHA and 6WMT were used as the primary outcome indicators for the comprehensive evaluation, and the results showed that CT + XML and CT + SM may be the best two treatments, followed by CT + SF, CT + YQFM, CT + HQ, and CT + SGM. Depending on the research focus and the primary outcome indicators, the conclusions may change accordingly.

In this study, there is no proof of publication bias. In addition, there are both direct comparison and indirect comparison evidence in the evaluation of six outcome indicators, including NYHA cardiac function classification efficiency, 6WMT, LVEF, CO, SV, and BNP. The consistency test results showed that, except for BNP, the consistency between the direct and indirect research evidence of other outcome indicators was good, and the evidence-based conclusions were stable and reliable. The increase in BNP is related to many diseases. In addition to HF, senile diseases such as atrial fibrillation (Silvet et al., 2003), chronic obstructive pulmonary disease (Inoue et al., 2009), and renal insufficiency (McCullough et al., 2003) can also cause non-specific increases in BNP. Although this study excluded patients with severe comorbidities as much as possible, the included population was mostly elderly patients, and individual differences had a greater impact on BNP levels, which may be the reason for the unstable evidence of BNP.

### Innovations and Limitations of This Study

At present, some researchers have performed NMAs on TCMIs for the treatment of HF (Wang et al., 2018; Yang et al., 2018). However, these studies did not consider the clinical heterogeneity between HF patients with different types of ejection fraction. A study showed that compared with heart failure with preserved ejection fraction (HFpEF), HFrEF is more related to high levels of BNP, troponin T, creatinine, uric acid, glycosylated hemoglobin levels, and the proportion of coronary heart disease (Liu et al., 2016). Considering that there may be significant differences in the pathophysiological process between HFrEF and HFpEF (Putko et al., 2021), we believe that research on different types of HF is more realistic for exploring personalized treatment plans. Therefore, we selected HFrEF with more severe clinical symptoms and more sufficient clinical data as the research object to reduce clinical heterogeneity and obtain more reliable evidence.

In addition, this study has some limitations: ① The overall quality of the included RCTs is low. Most RCTs did not report in detail on random methods, blinding, allocation concealment, and program registration. These limitations may lead to serious risks of bias. Therefore, it is recommended that clinical research should be implemented strictly in accordance with the CONSORT checklist (Moher et al., 2012). ② Due to the lack of clinical data, this study failed to report outcomes that directly reflect the clinical benefit and prognosis of patients, such as mortality and rehospitalization rates. ③ Since all the studies were conducted in China, the external adaptability of the conclusions will be limited to a certain extent.

## Conclusion

In summary, the NMA results show that compared with CT alone, the combination of qualified TCMIs and CT is more effective for HFrEF patients. Among them, CT + XML and CT + SM may be the potential optimal treatment. Besides, the safety of these TCMIs needs to be further observed through more studies using unified observation indicators that reflect safety. However, due to some limitations, the conclusions need to be verified by more large-sample, double-blind, multi-center RCTs in the future.
