# Supplementary figures and images for: Efficacy and Safety of Traditional Chinese Medicine Injections for Heart Failure With Reduced Ejection Fraction: A Bayesian Network Meta-Analysis of Randomized Controlled Trials
Source: Front Pharmacol. 2021 Nov 30;12:659707. doi: 10.3389/fphar.2021.659707 (PMC8669995; doi:10.3389/fphar.2021.659707)

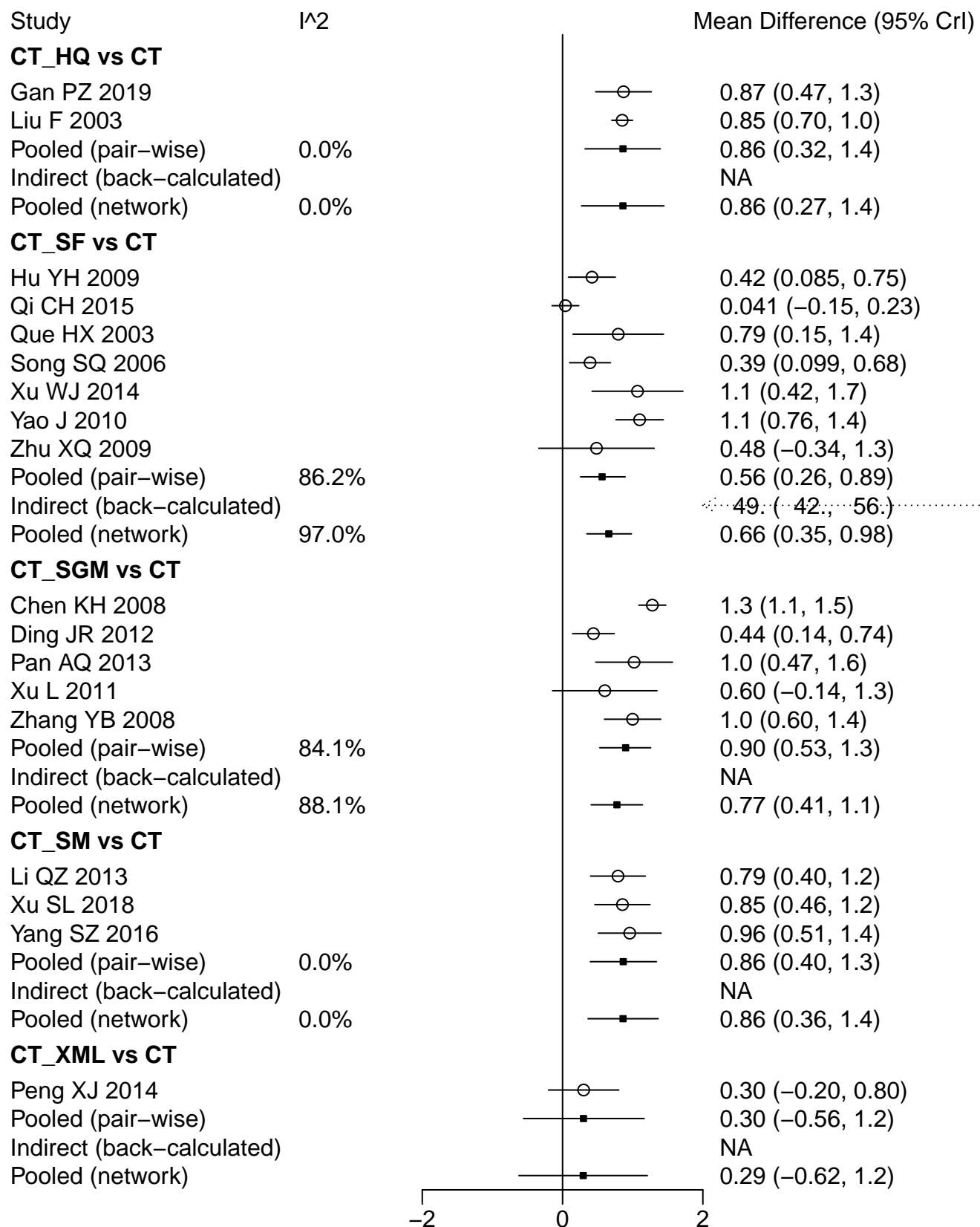

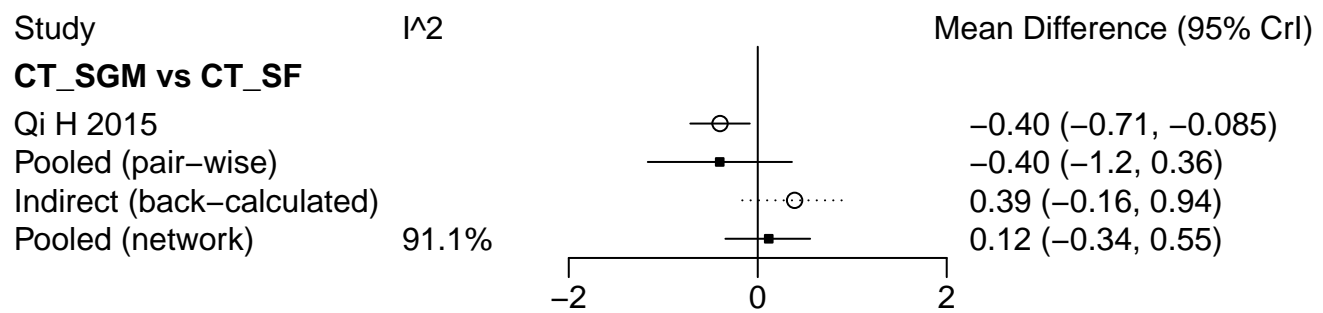

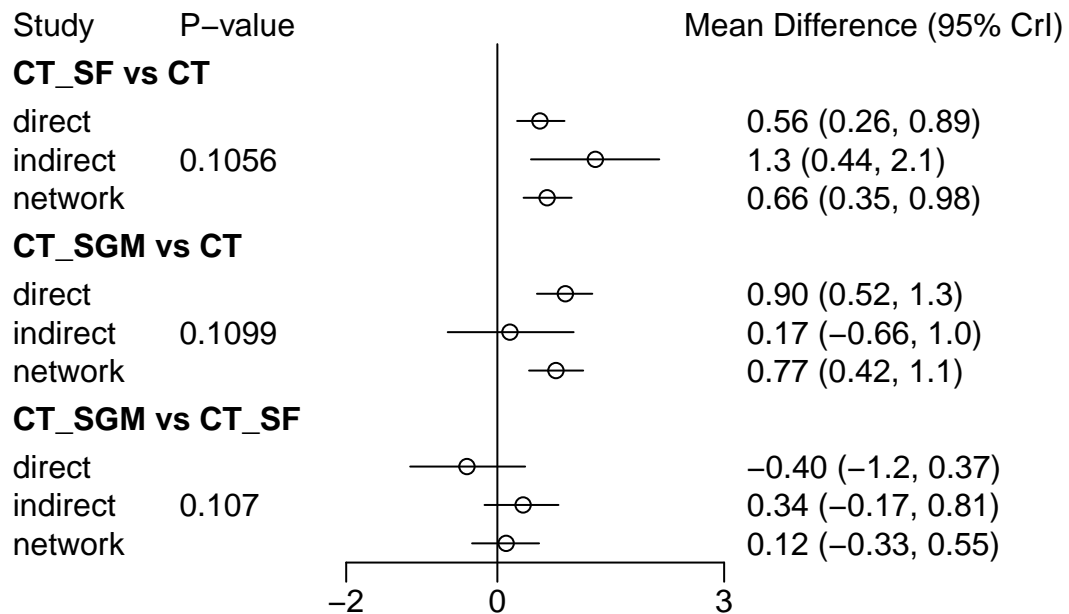

Supplement: Supplementary file 1 [file DataSheet7.PDF]

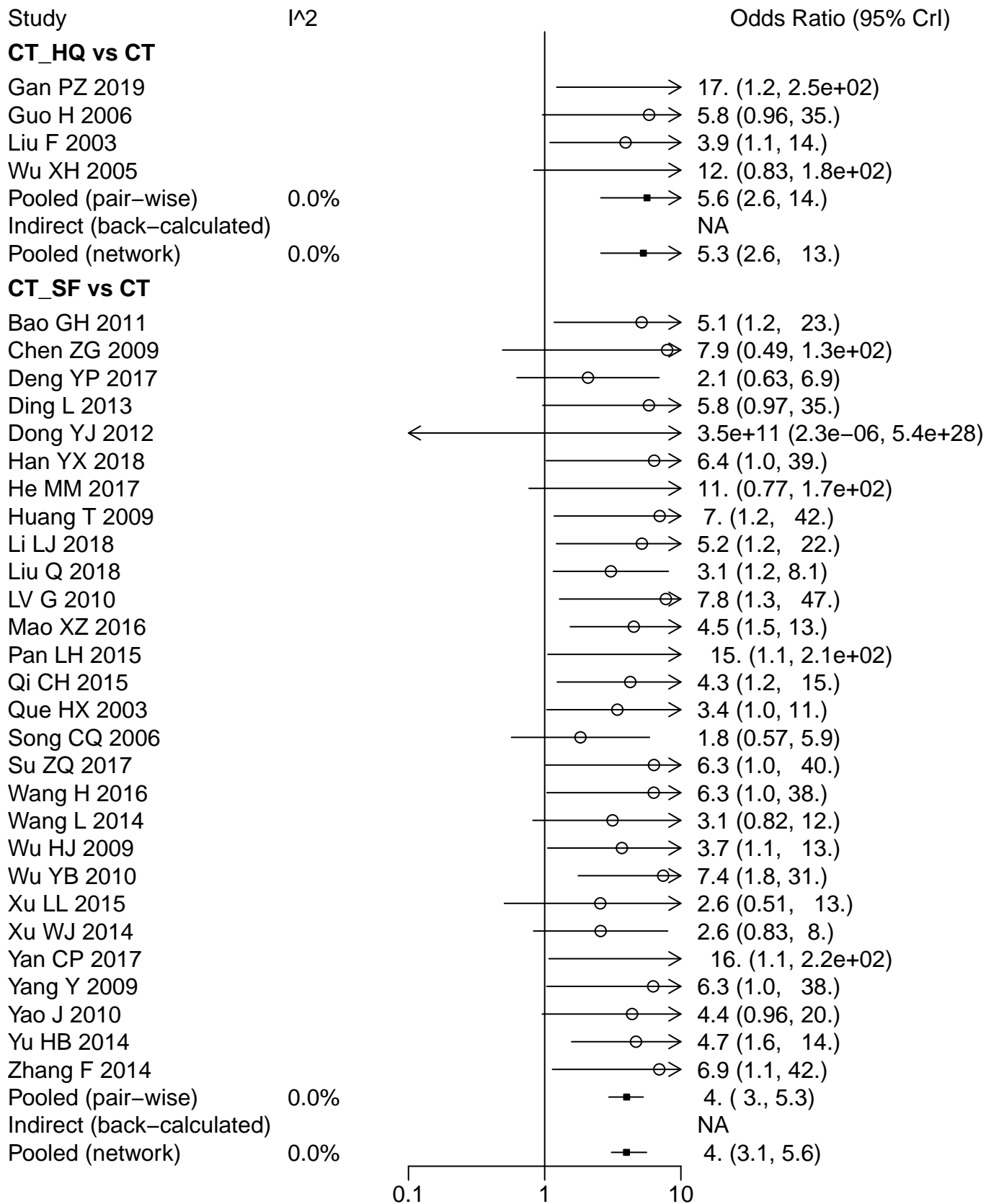

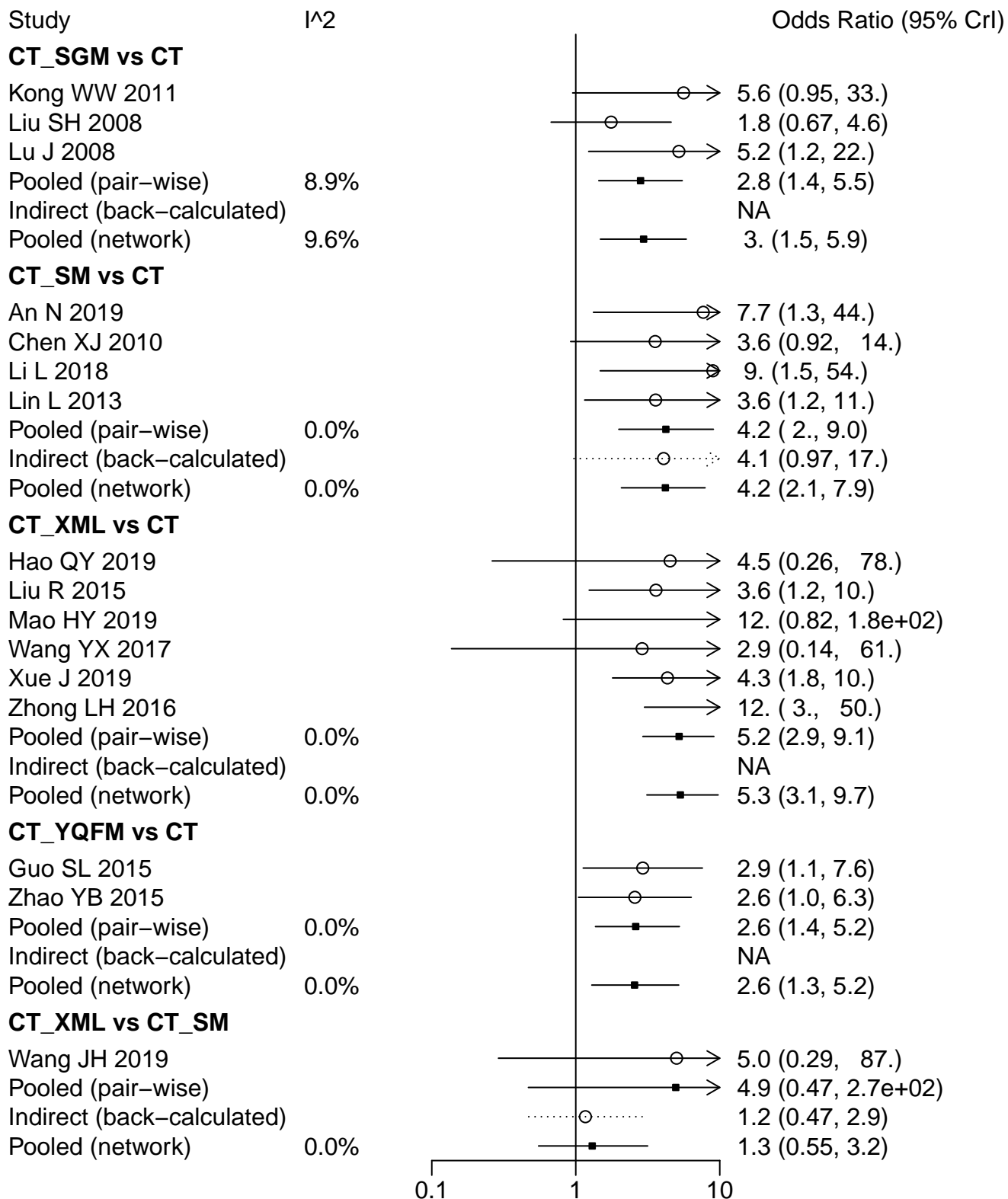

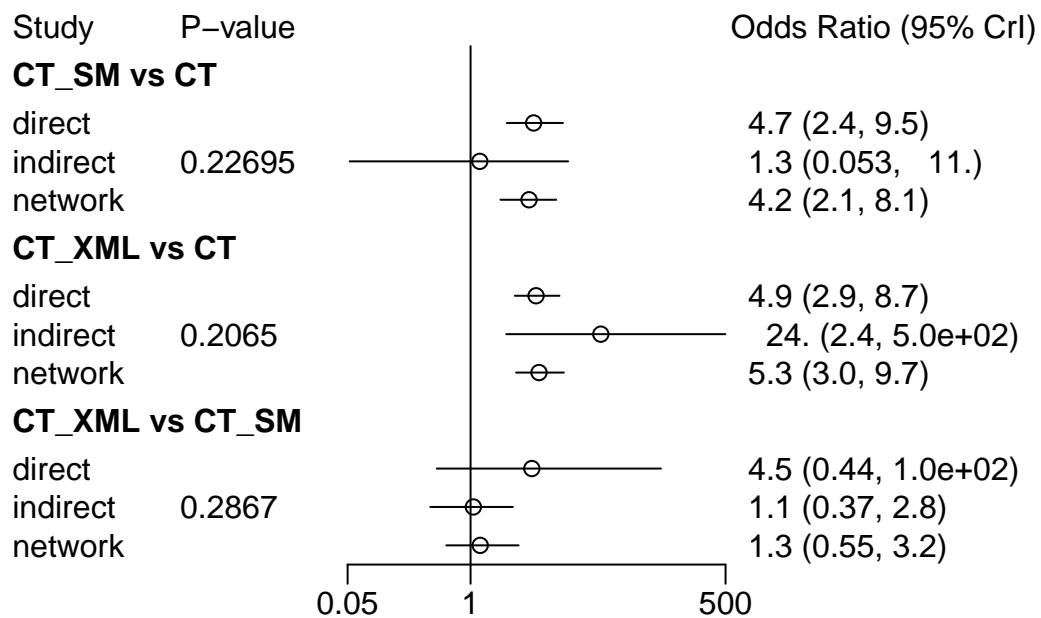

Supplement: Supplementary file 3 [file DataSheet4.PDF]

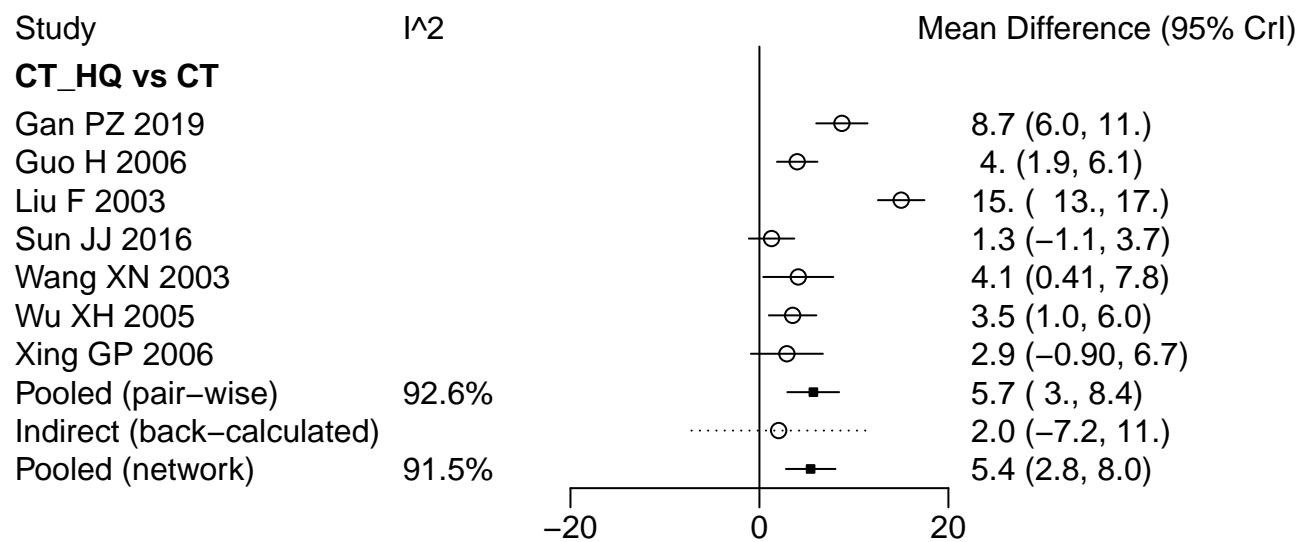

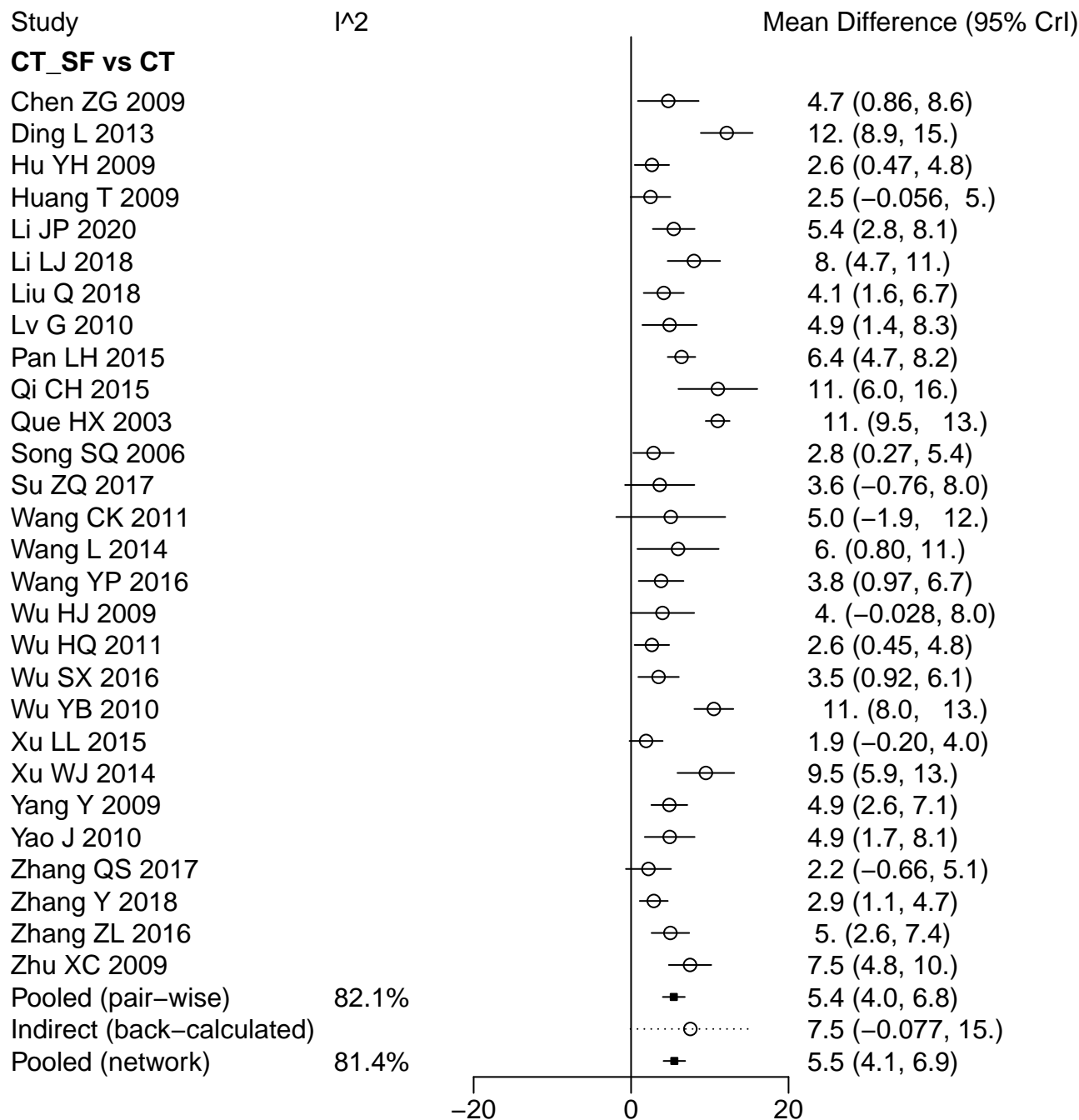

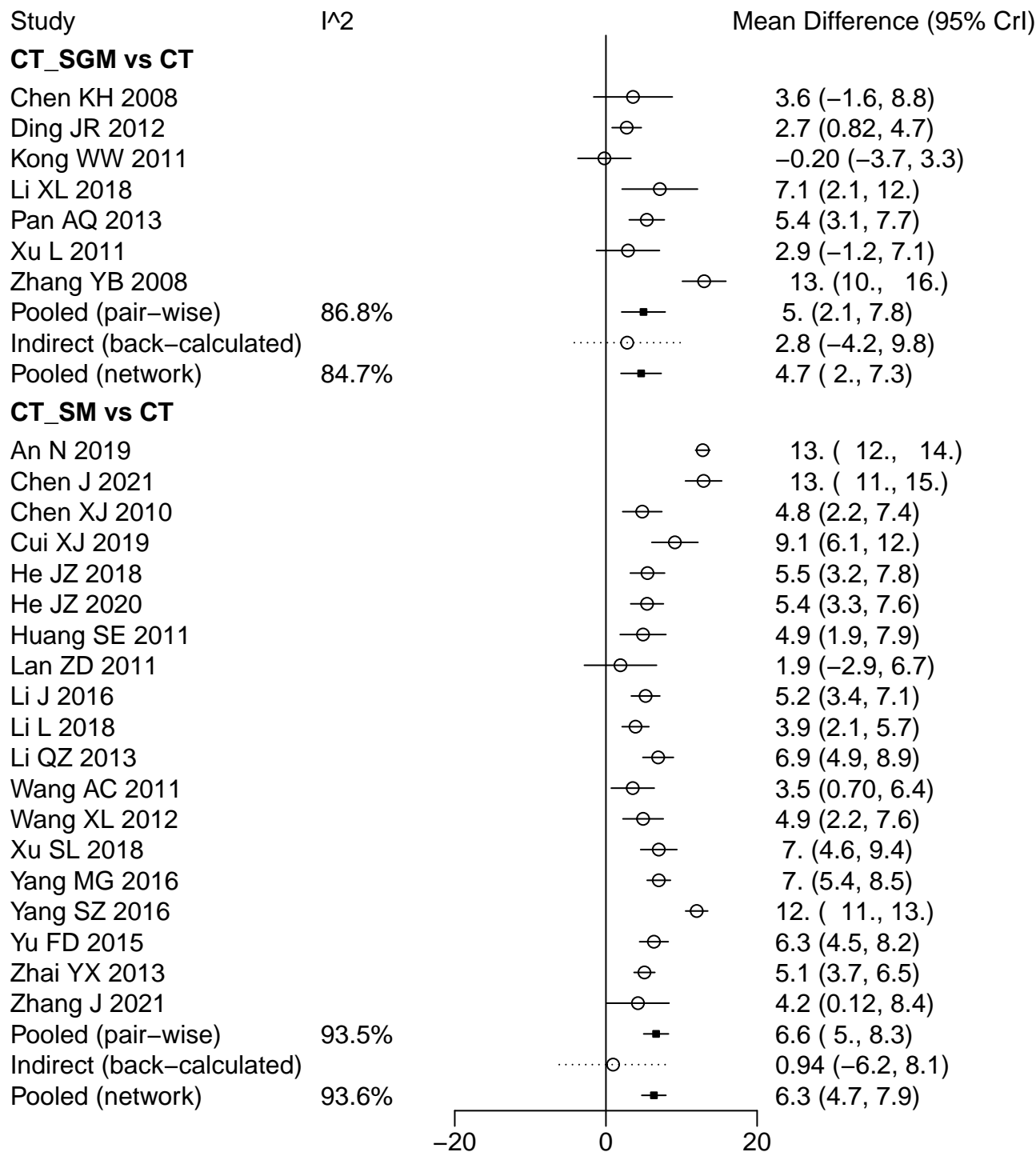

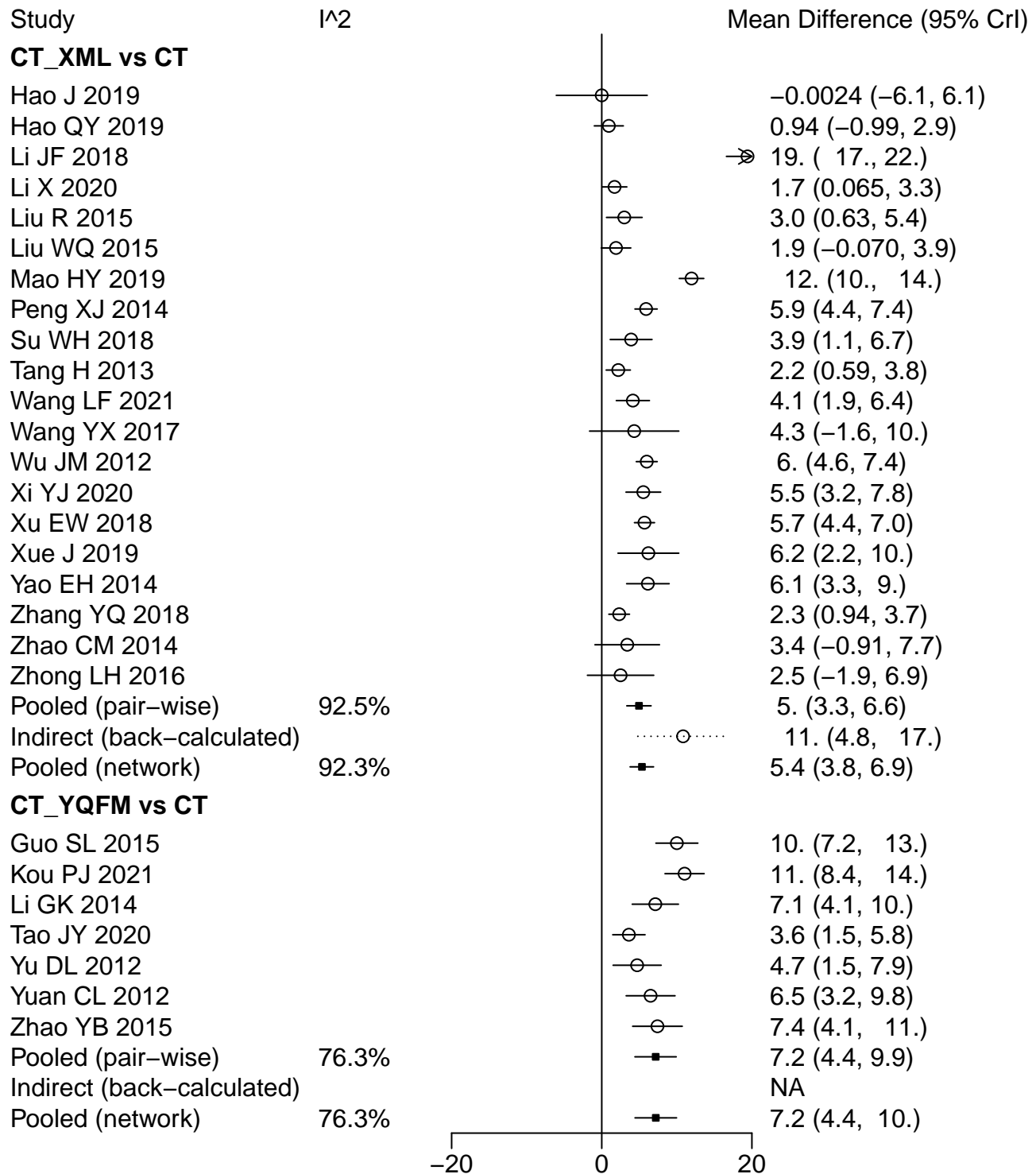

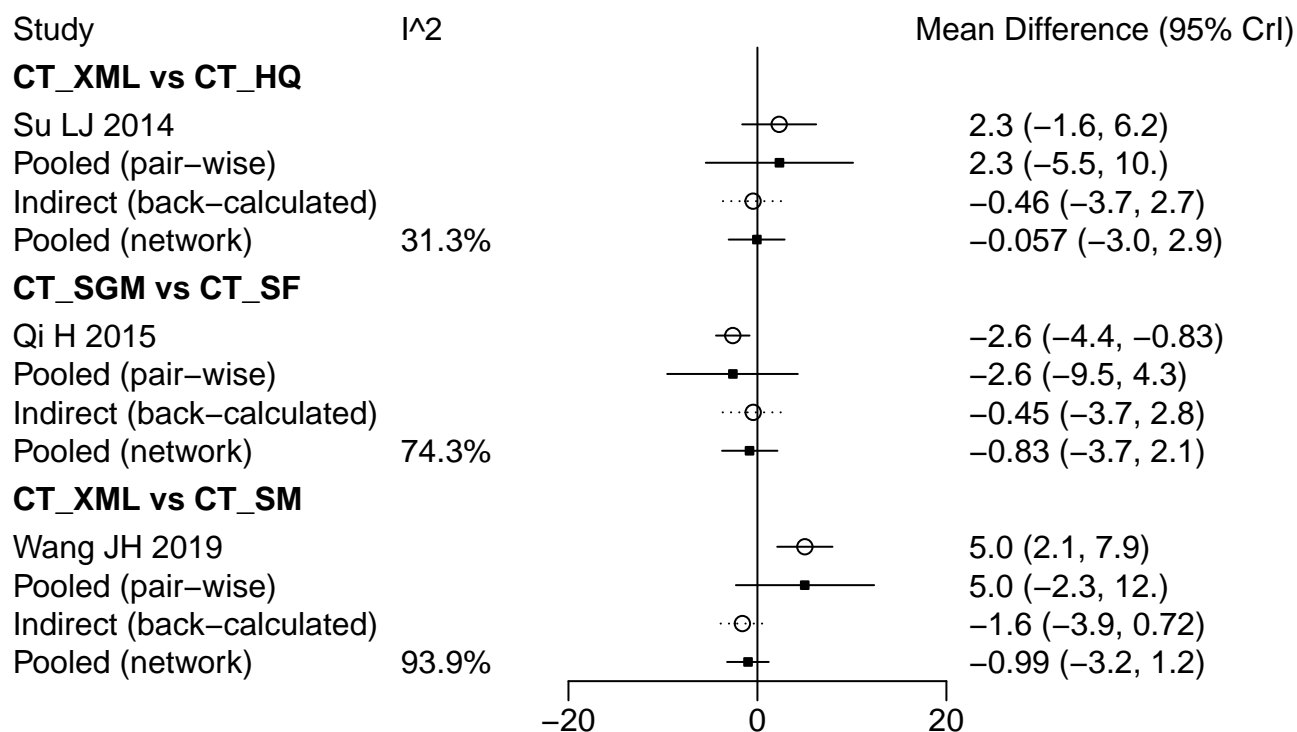

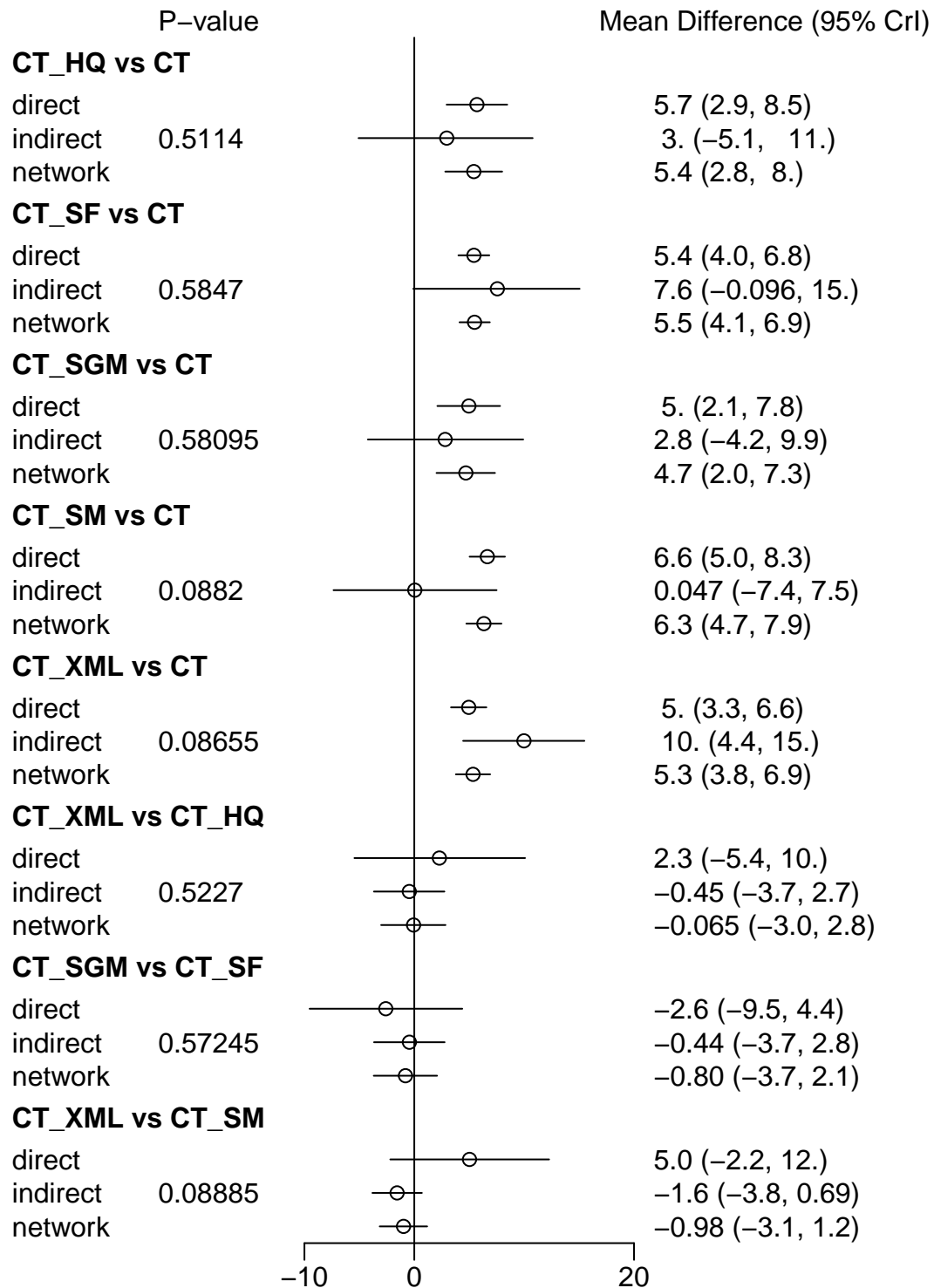

Supplement: Supplementary file 4 [file DataSheet6.PDF]

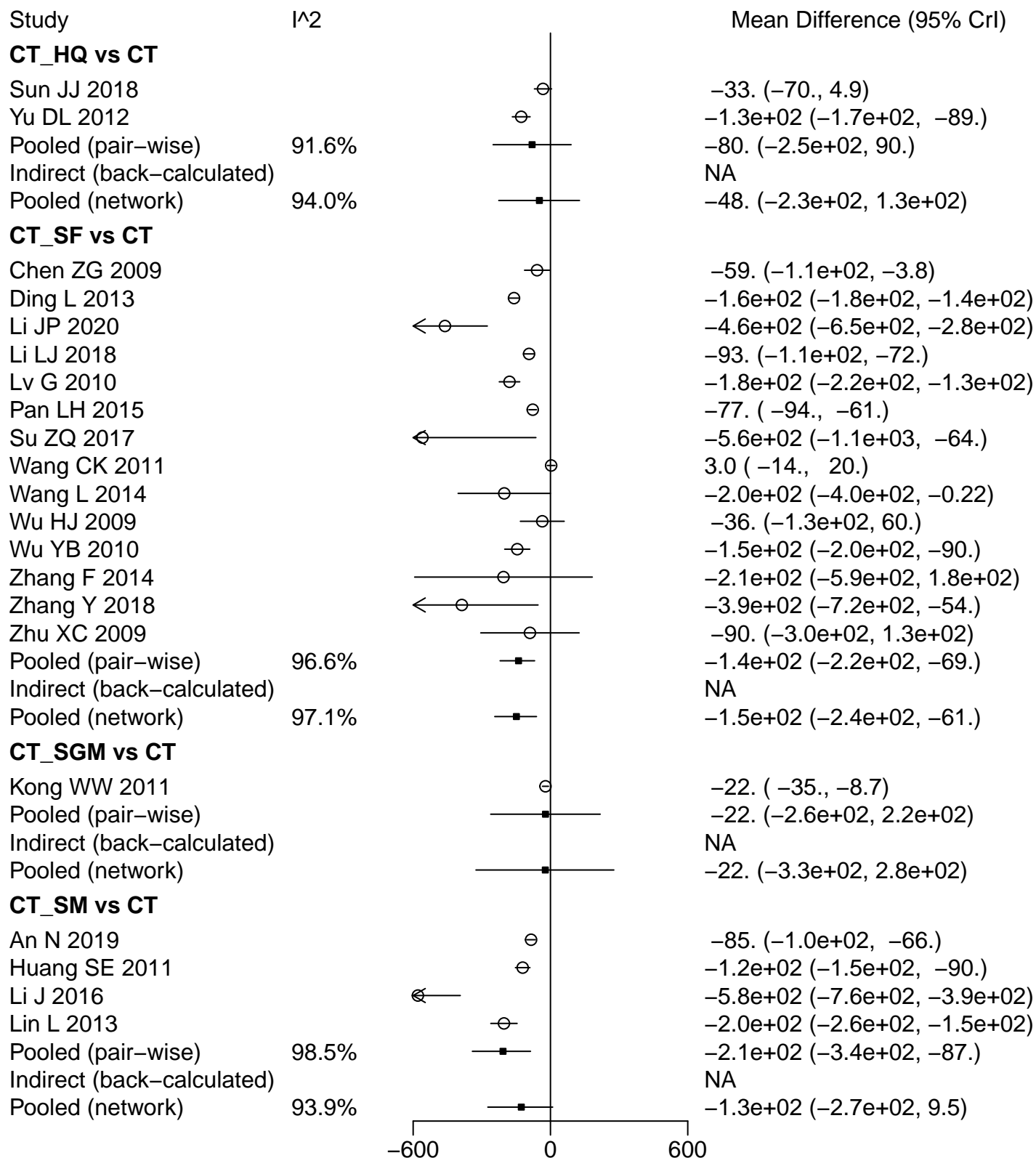

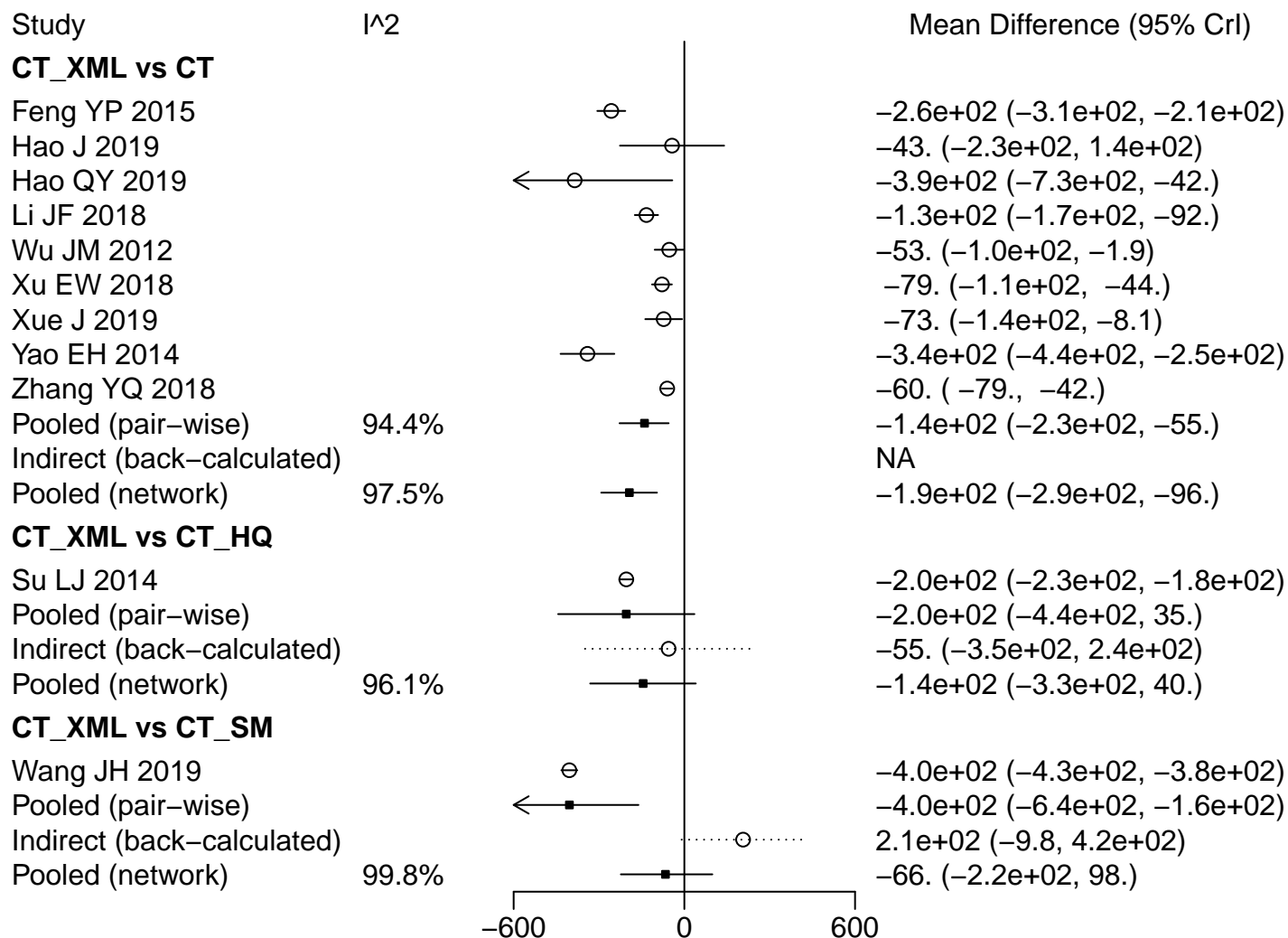

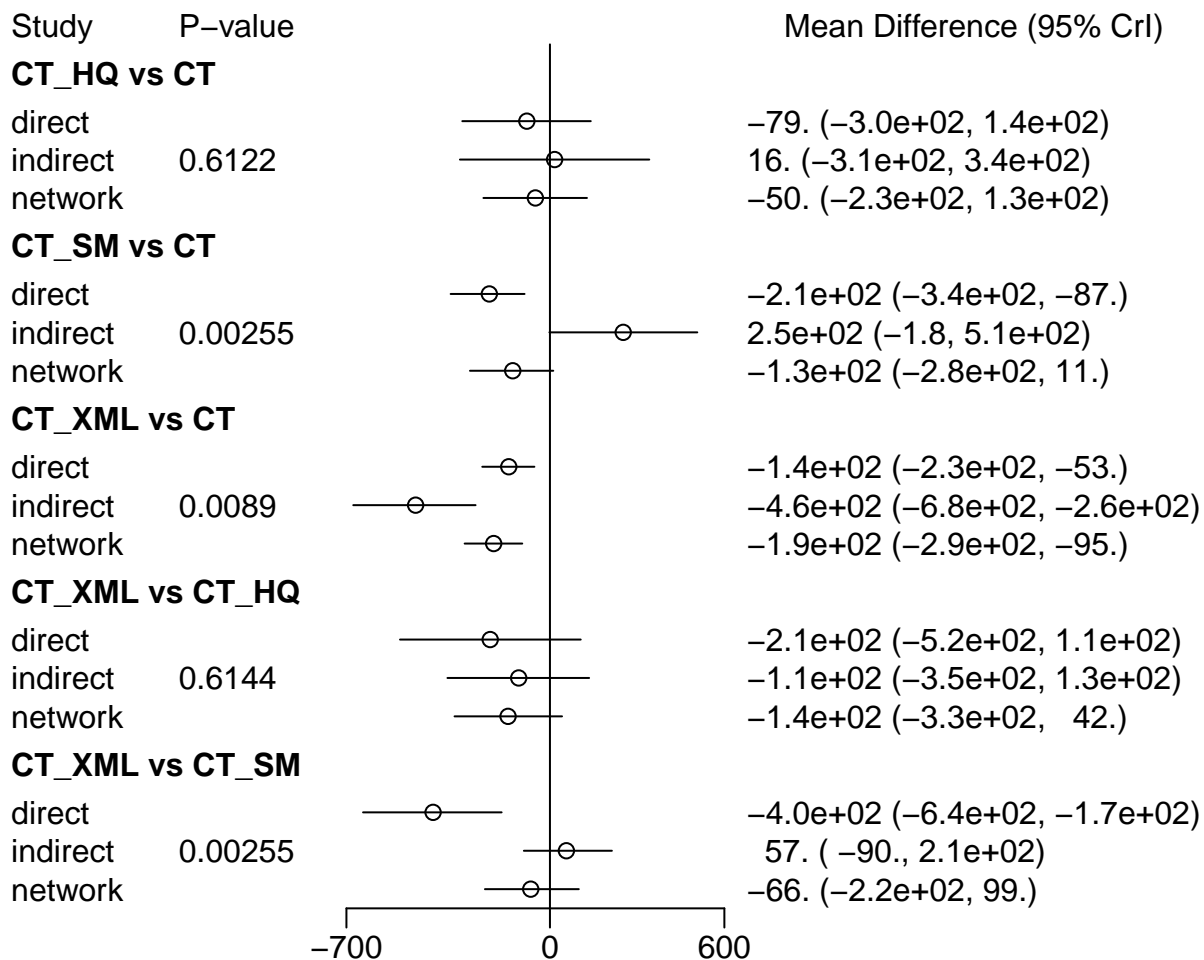

Supplement: Supplementary file 5 [file DataSheet9.PDF]

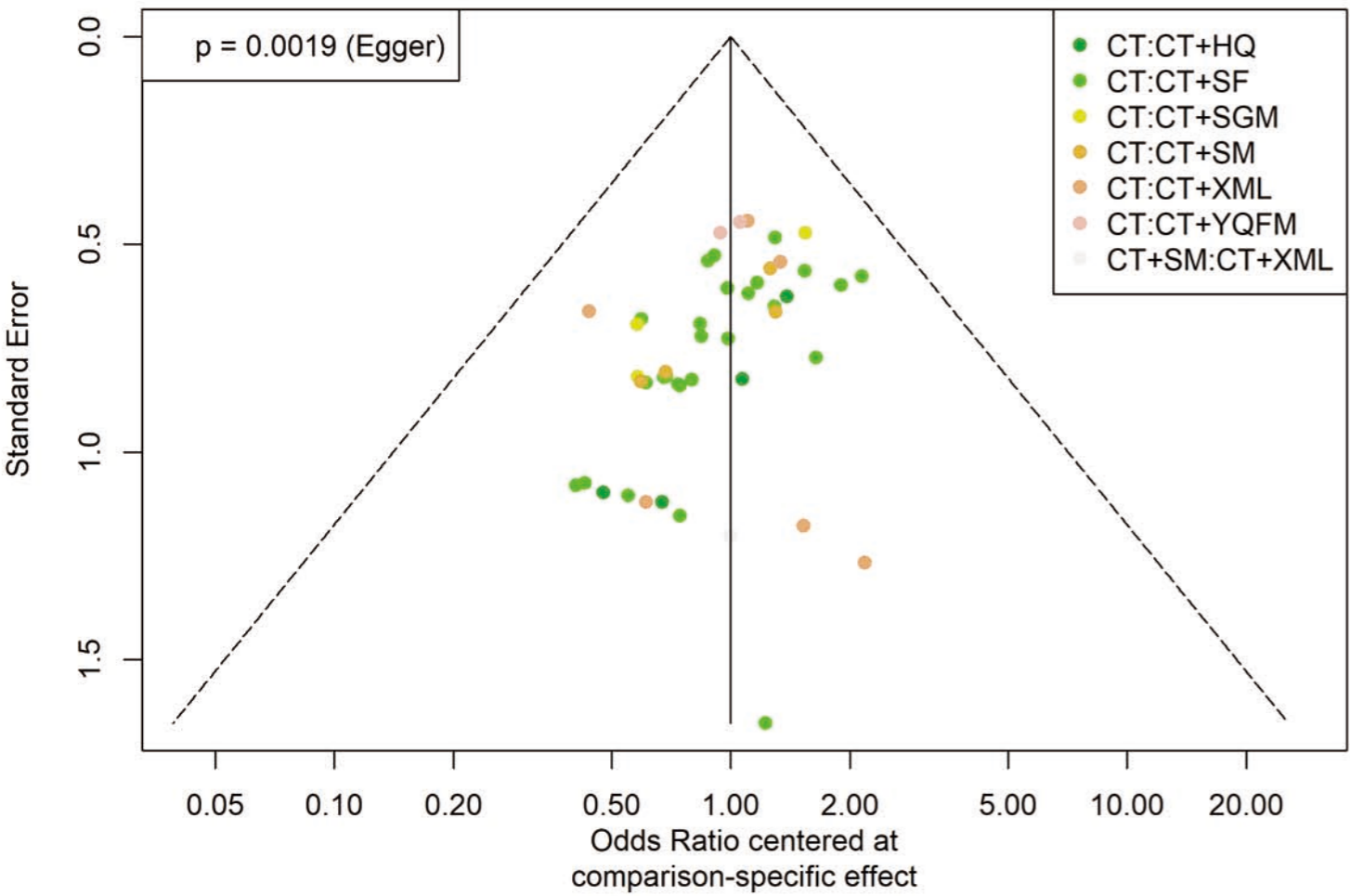

NYHA

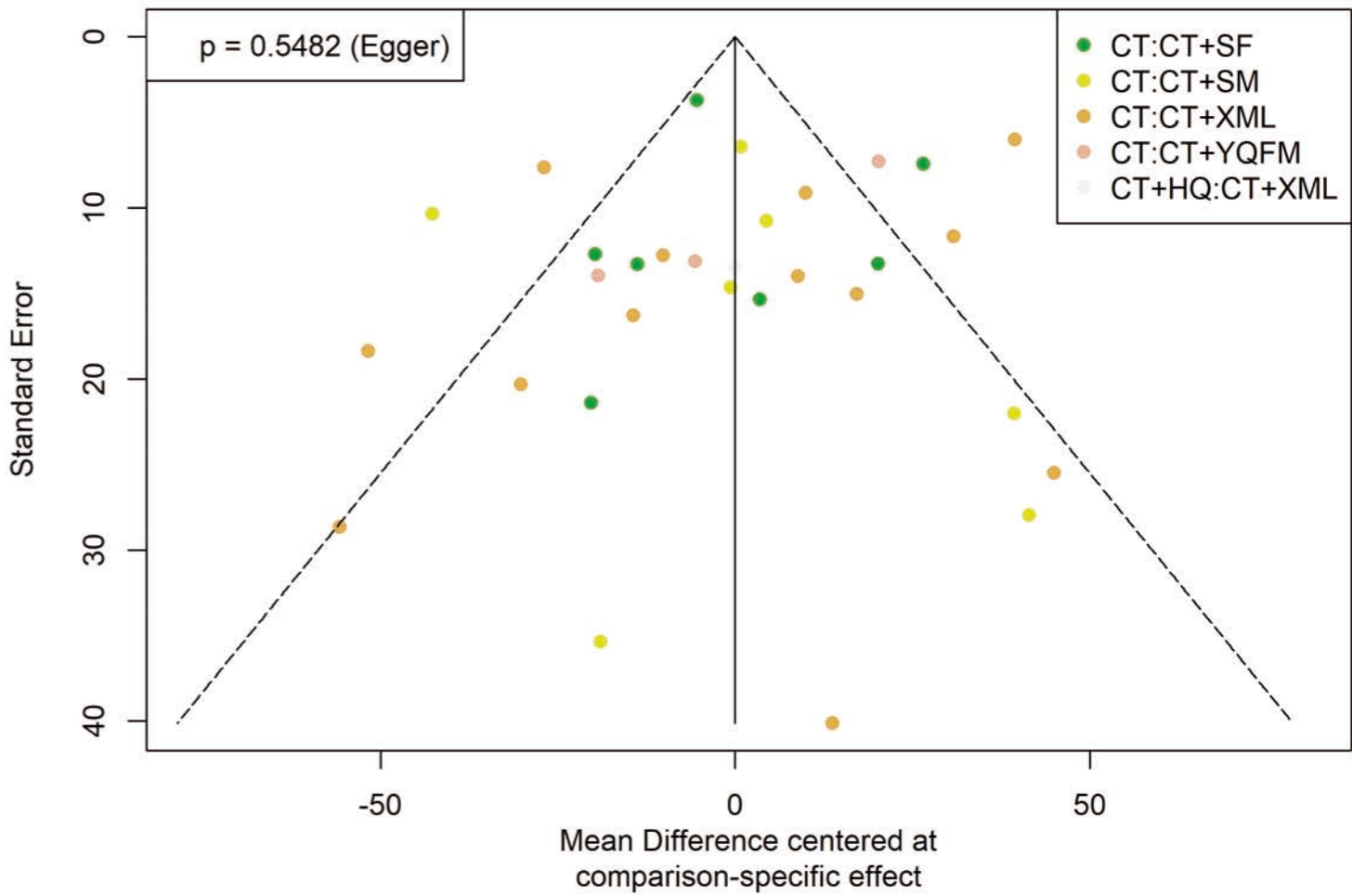

6WMT

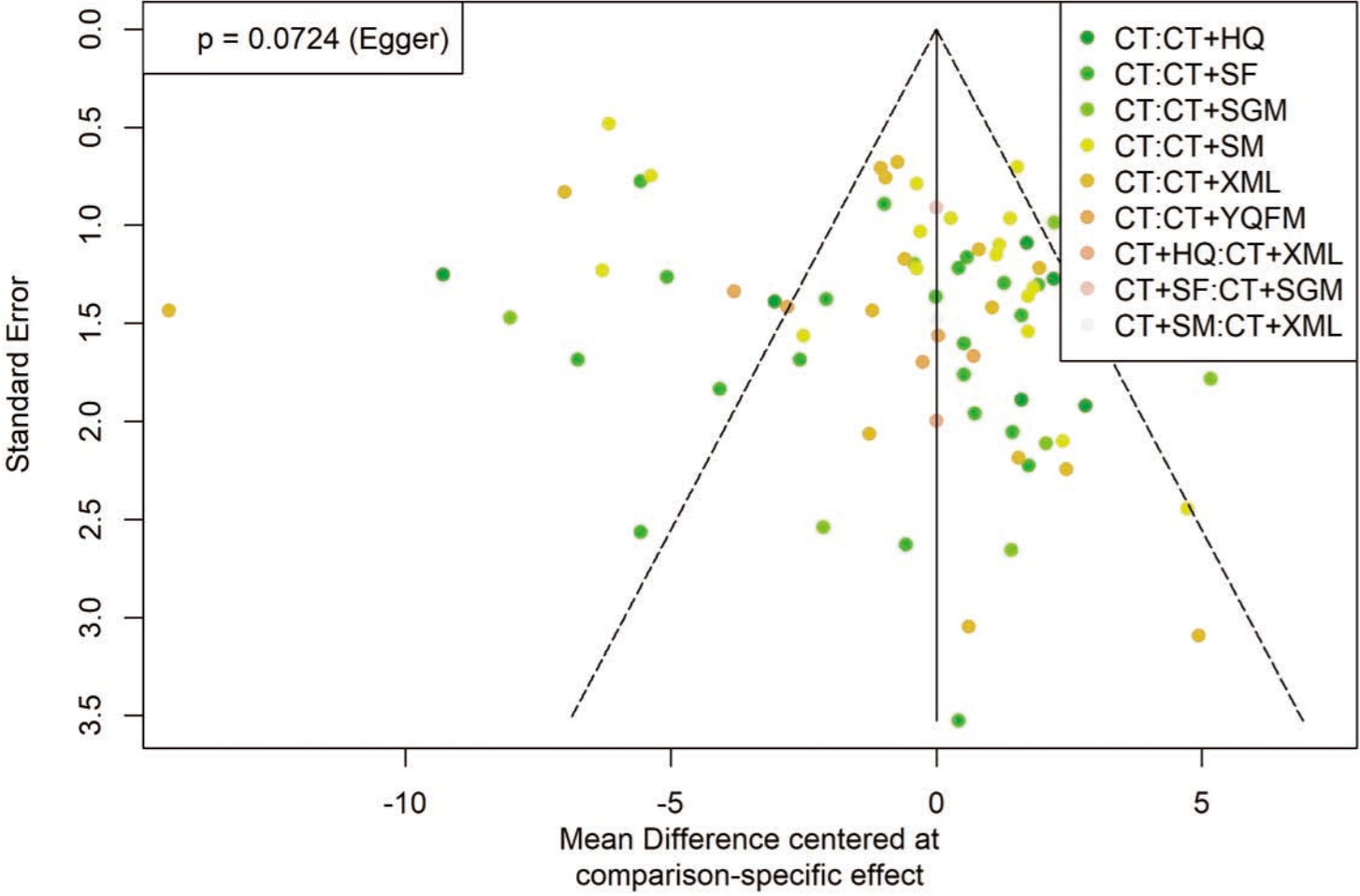

LVEF

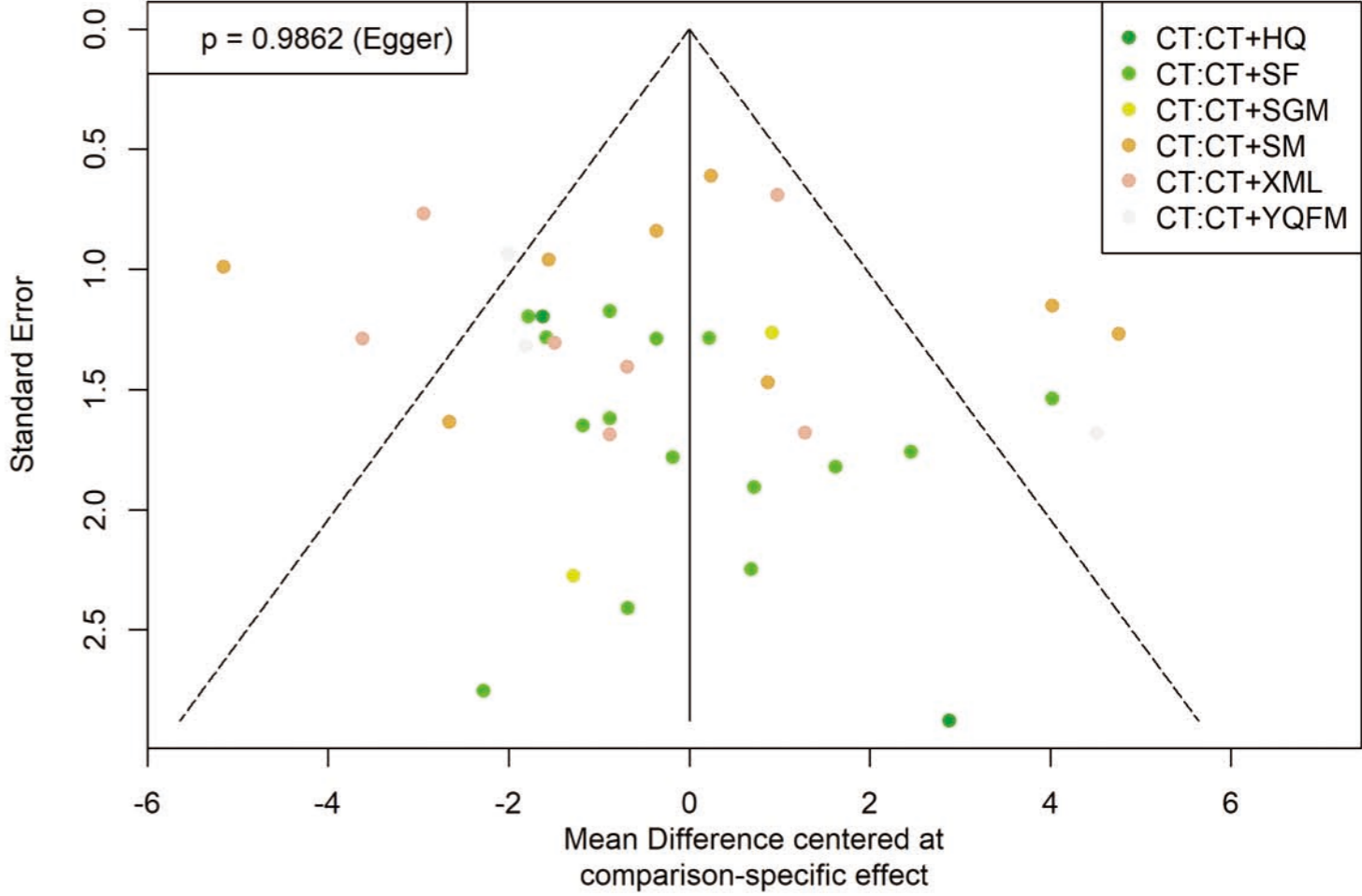

LVEDD

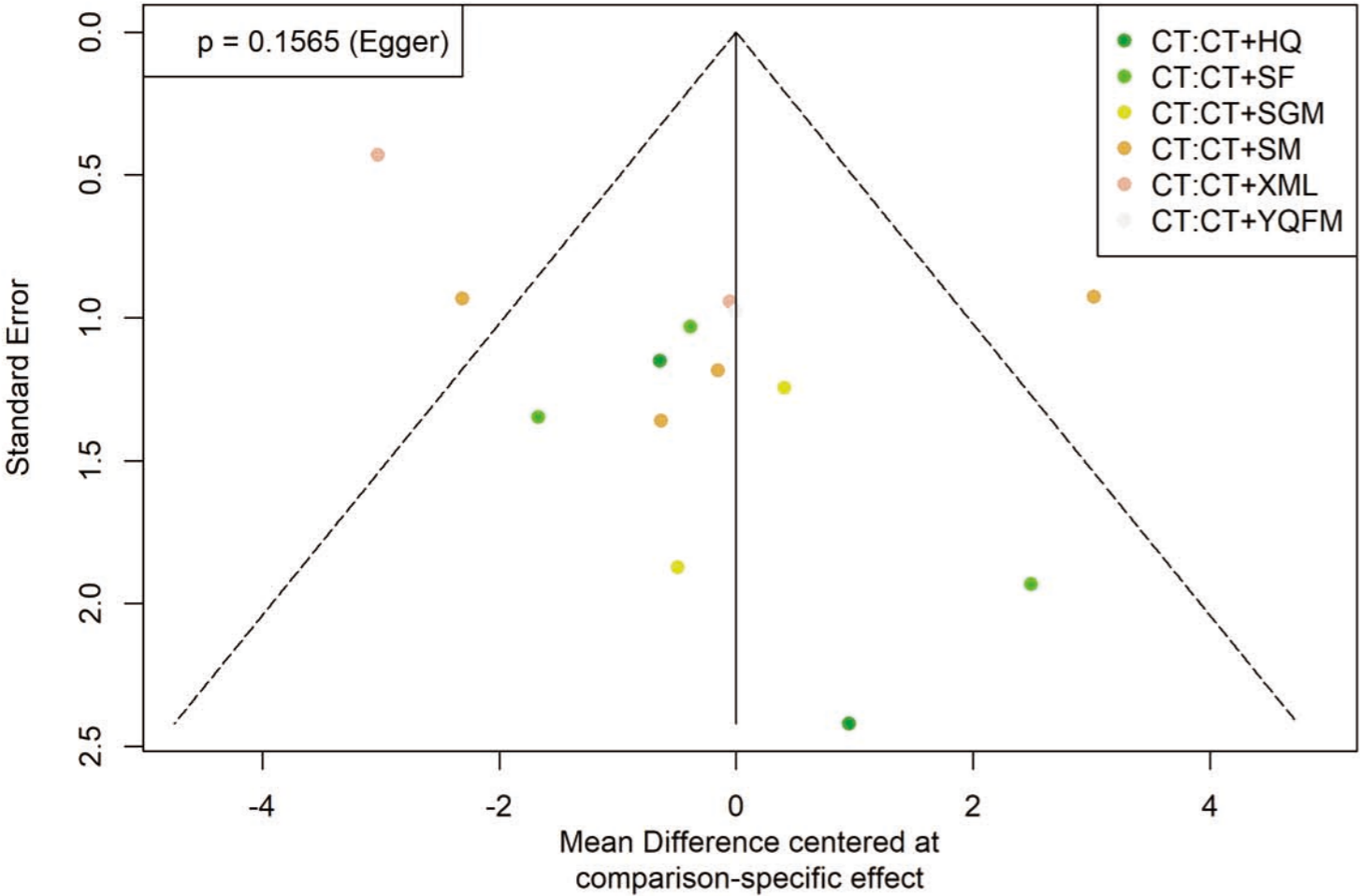

LVESD

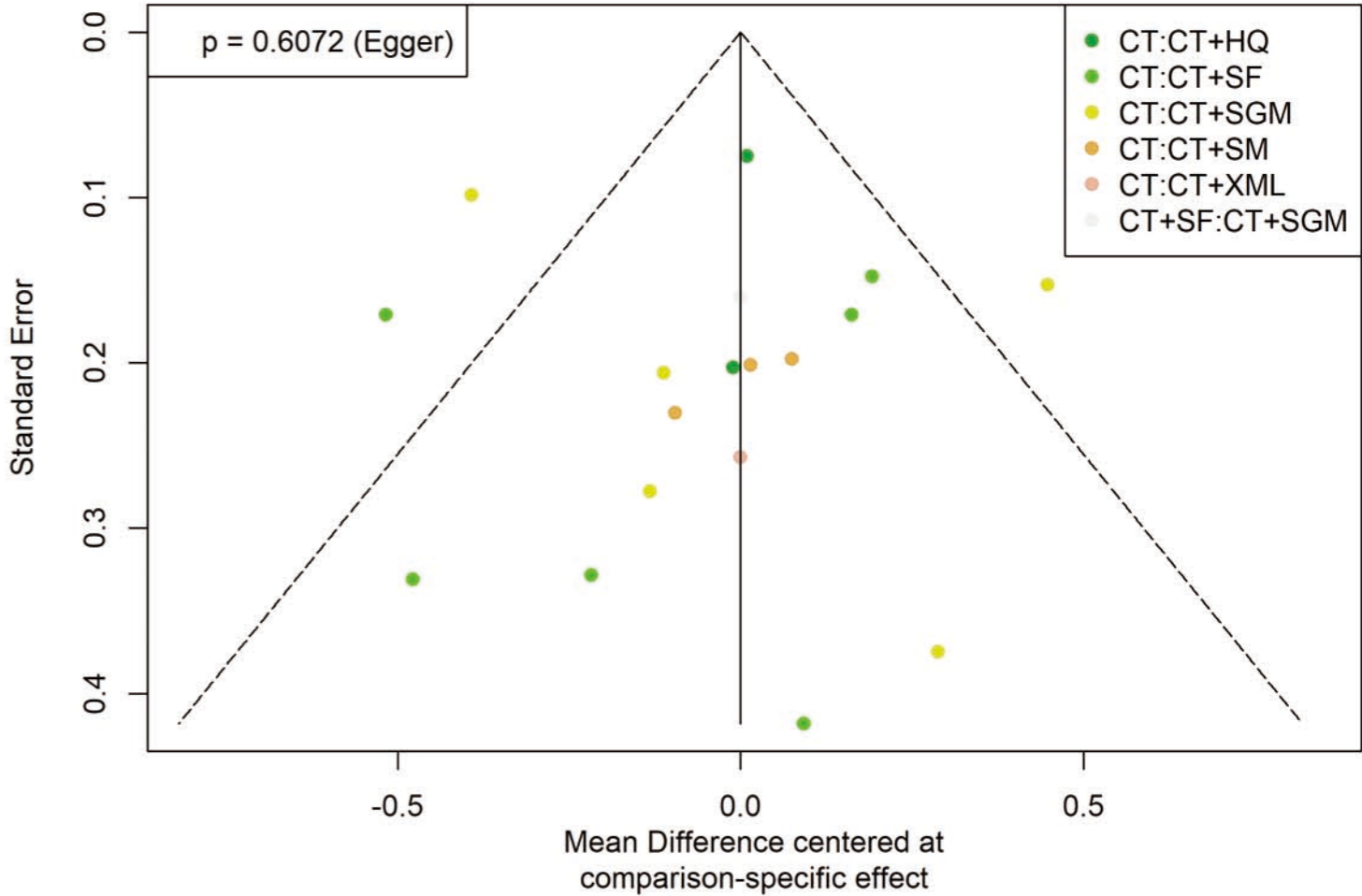

CO

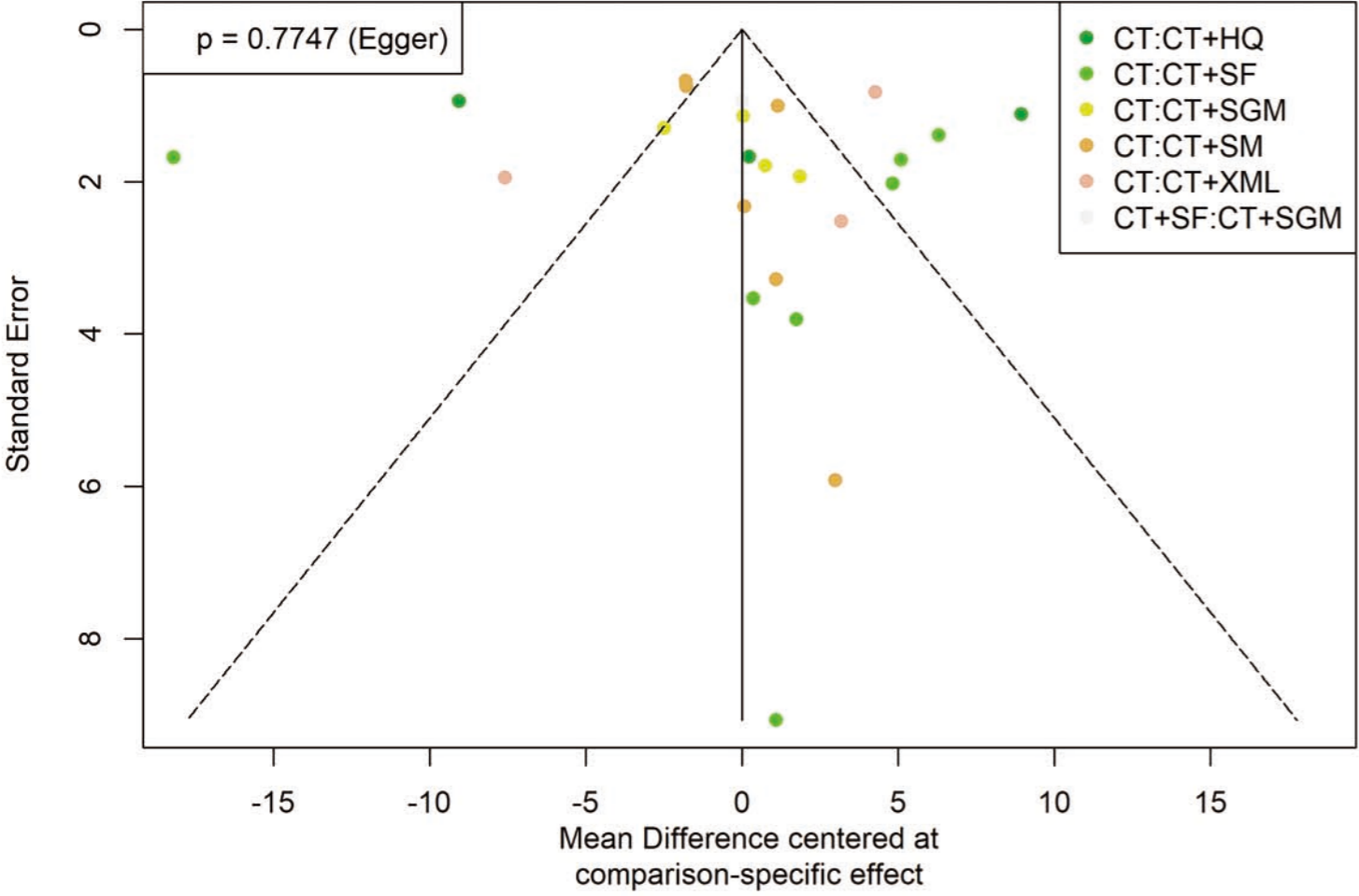

SV

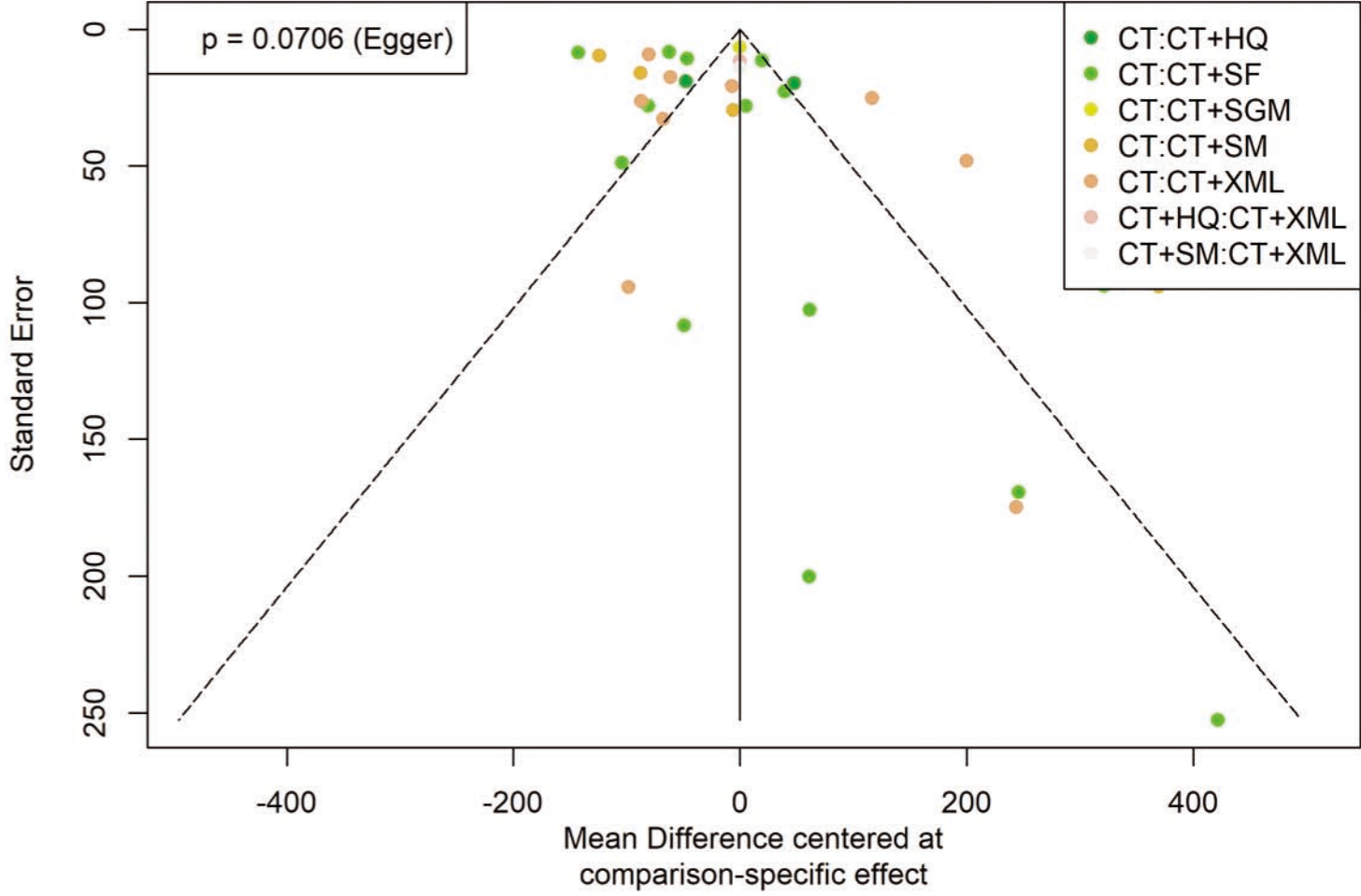

BNP

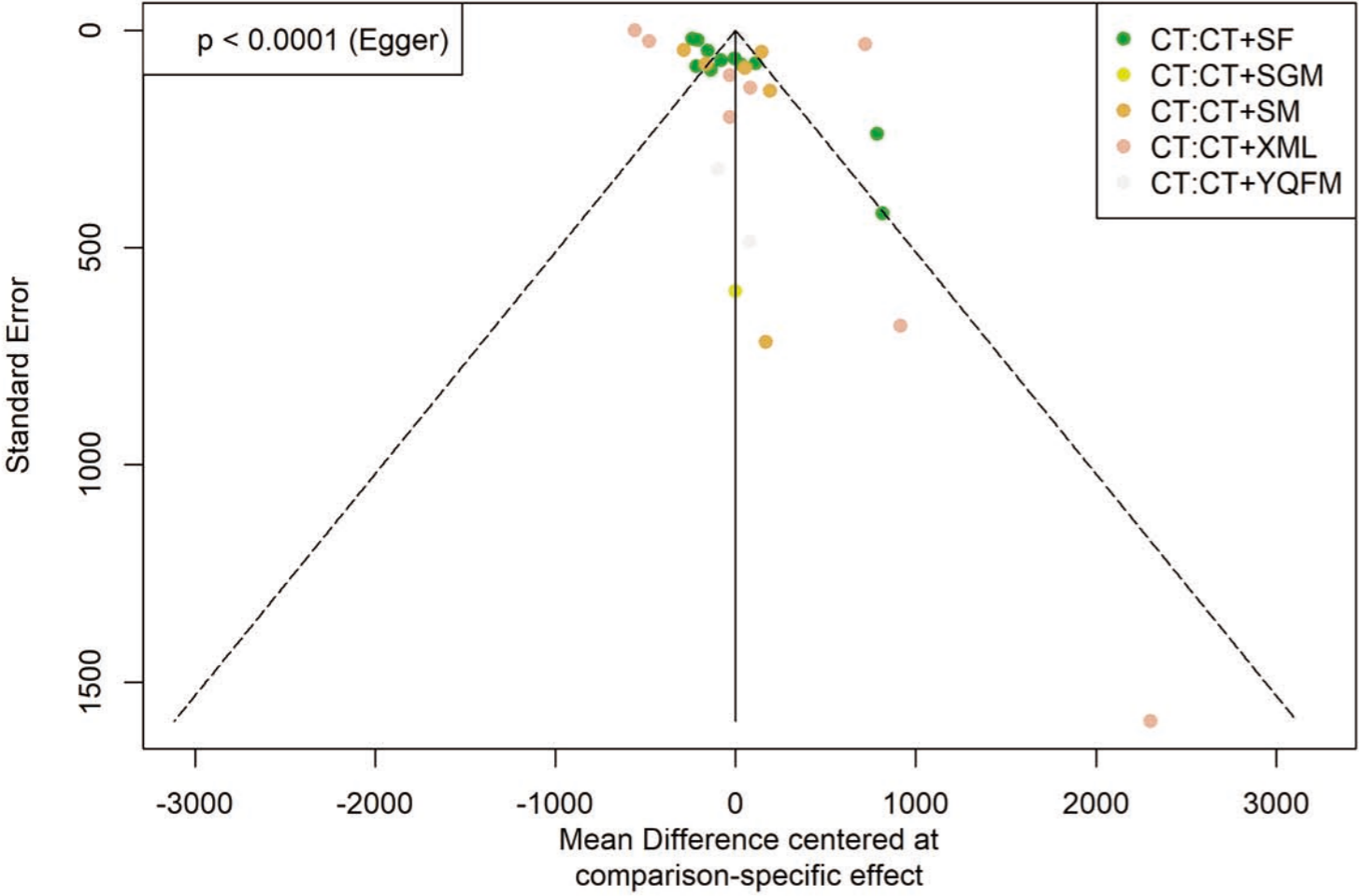

NT-proBNP

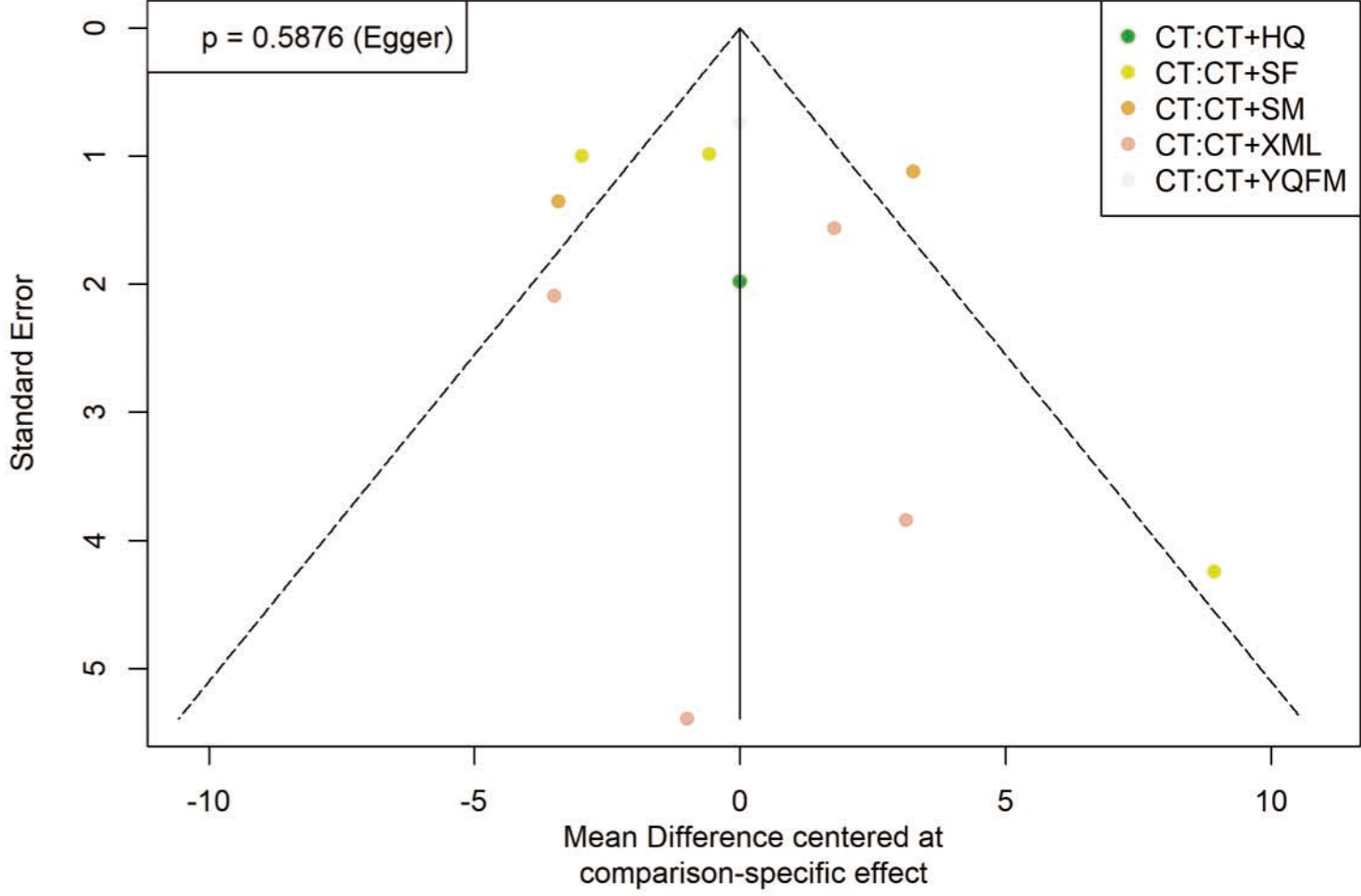

MLHFQ

Supplement: Supplementary file 6 [file DataSheet3.PDF]

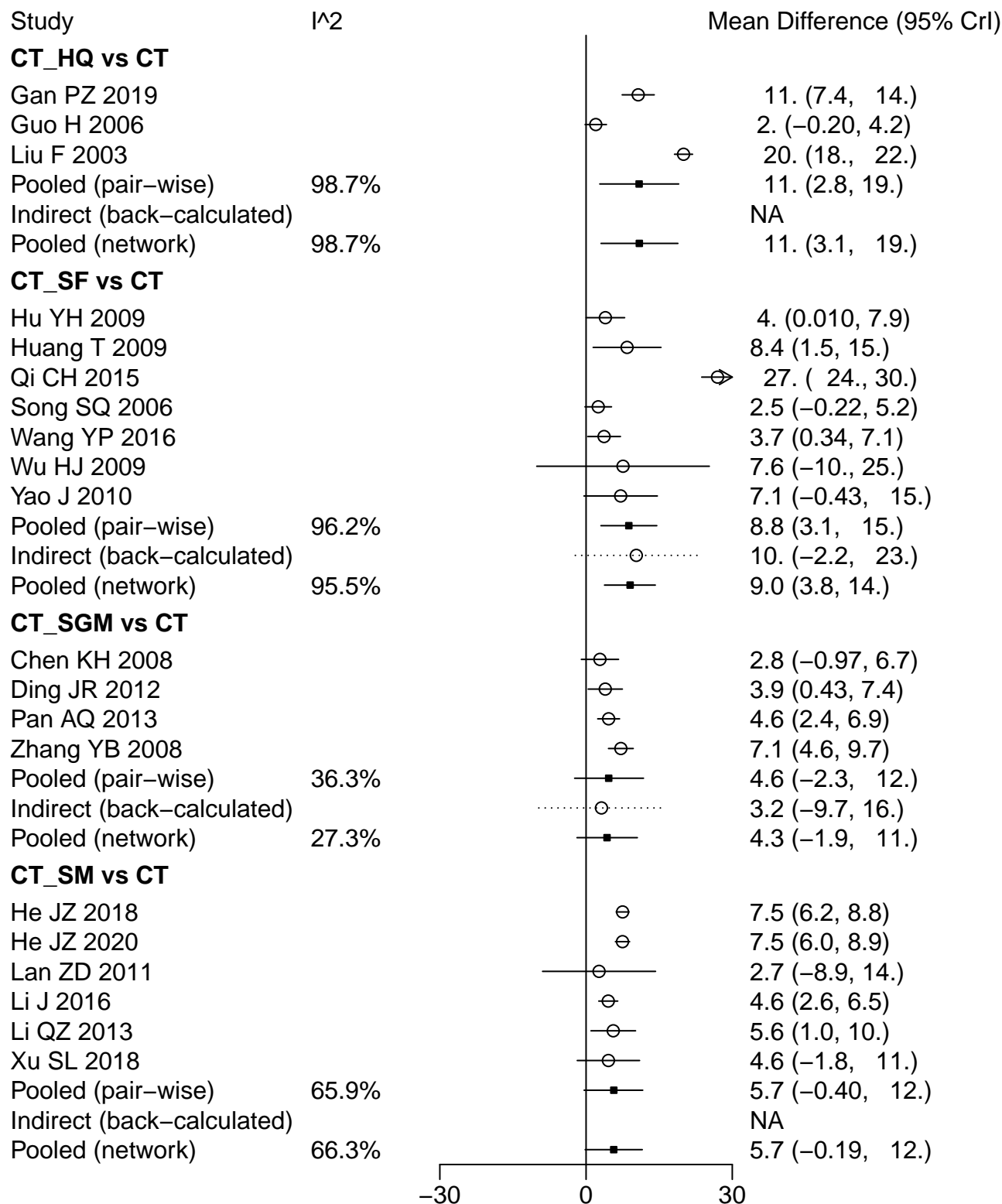

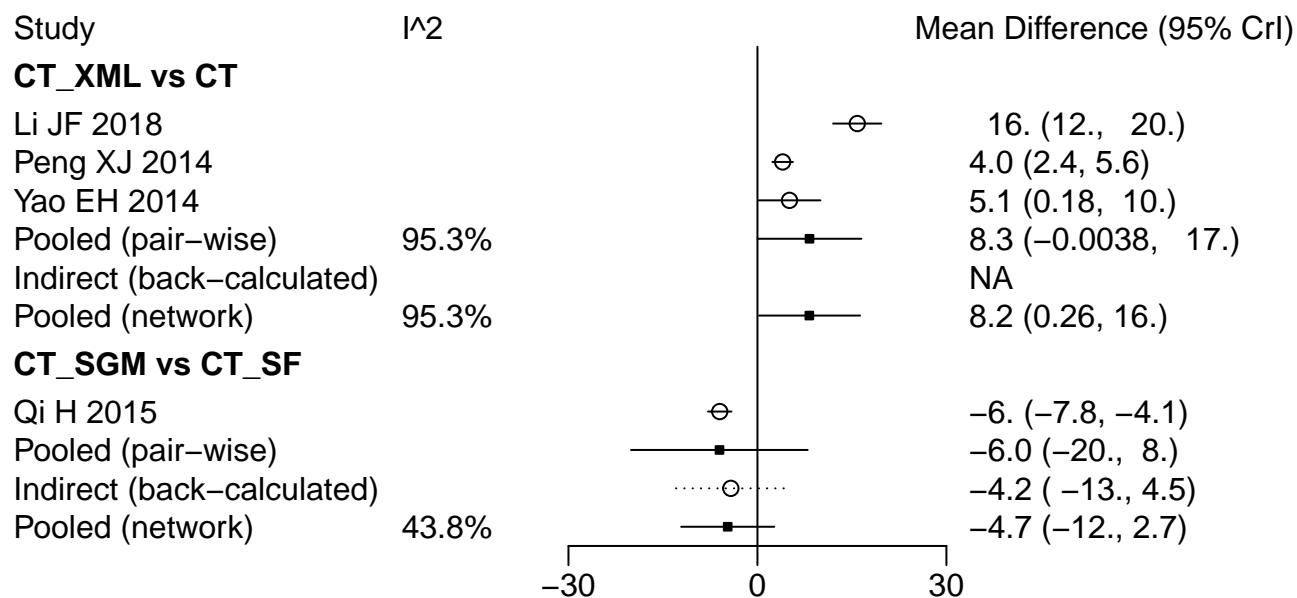

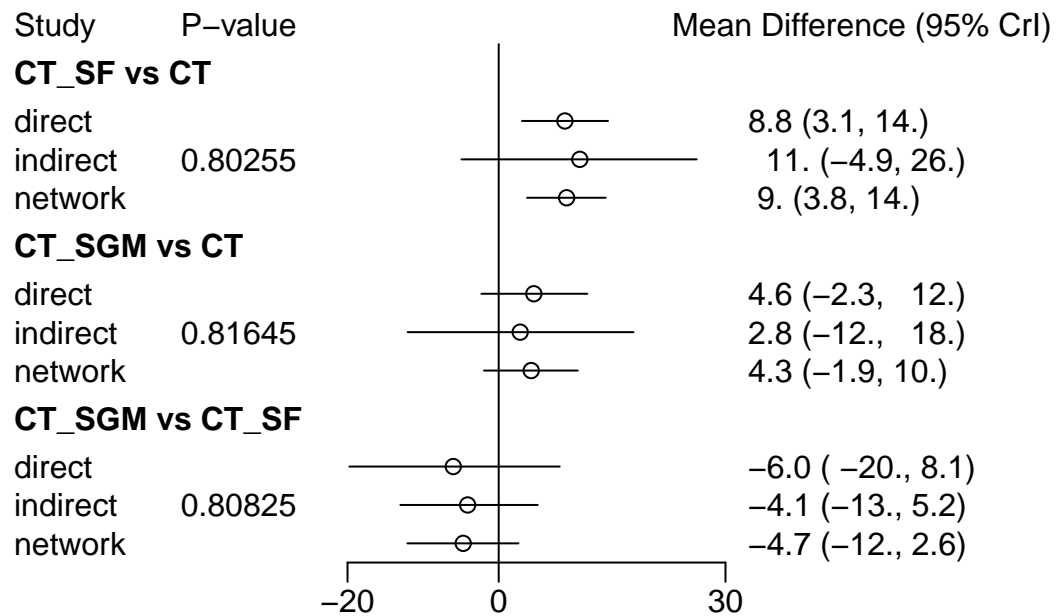

Supplement: Supplementary file 9 [file DataSheet8.PDF]
