# Supplementary material for: Efficacy and Safety of Traditional Chinese Medicine Injections for Heart Failure With Reduced Ejection Fraction: A Bayesian Network Meta-Analysis of Randomized Controlled Trials
Source: Front Pharmacol. 2021 Nov 30;12:659707. doi: 10.3389/fphar.2021.659707 (PMC8669995; doi:10.3389/fphar.2021.659707)
Supplement: Supplementary file 2 [file DataSheet2.PDF]

Supplementary File 2 Search Strategy in PubMed

Search	Query	Items found
#1	heart failure[MeSH Terms] OR heart failure[Title/Abstract] OR cardiac failure[MeSH Terms] OR cardiac failure[Title/Abstract] OR systolic heart failure[MeSH Terms] OR systolic heart failure[Title/Abstract] OR heart failure, systolic[MeSH Terms] OR heart failure, systolic[Title/Abstract] OR heart decompensation[MeSH Terms] OR heart decompensation[Title/Abstract] OR cardiac decompensation[MeSH Terms] OR cardiac decompensation[Title/Abstract] OR heart dysfunction[Title/Abstract] OR cardiac dysfunction[Title/Abstract] OR myocardial failure[MeSH Terms] OR myocardial failure[Title/Abstract] OR myocardiac dysfunction[Title/Abstract] OR cardiomyopathy[MeSH Terms] OR cardiomyopathy[Title/Abstract] OR ventricular dysfunction[MeSH Terms] OR ventricular dysfunction[Title/Abstract] OR heart dificiency[Title/Abstract] OR cardiac dificiency[Title/Abstract] OR heart insufficiency[MeSH Terms] OR heart insufficiency[Title/Abstract] OR cardiac insufficiency[MeSH Terms] OR cardiac insufficiency[Title/Abstract]	368,749
#2	Shenfu[All Fields] OR Shenmai[All Fields] OR Xinmailong[All Fields] OR Shengmai[All Fields] OR Huangqi[All Fields] OR Astragalus[All Fields] OR Qiyifumai[All Fields] OR Qiyi Fumai[All Fields]	11,216
#3	injection[MeSH Terms] OR injection$[Title/Abstract] OR injectable$[Title/Abstract]	708,382
#4	randomized controlled trial[Publication Type] OR controlled clinical trial[Publication Type] OR clinical trial[Publication Type] OR equivalence trial[Publication Type] OR randomized controlled trials as topic[Mesh Terms] OR randomized controlled trial[Mesh Terms] OR randomized controlled trial[Title/Abstract] OR clinical trials, randomized[Mesh Terms] OR clinical trials, randomized[Title/Abstract] OR controlled clinical trials, randomized[Mesh Tems] OR controlled clinical trials, randomized[Title/Abstract] OR random allocation[Mesh Terms]OR random allocation[Title/Abstract] OR double-blind method[Mesh Terms] OR double-blind method[Title/Abstract] OR single-blind method[Mesh Terms] OR single-blind method[Title/Abstract] OR placebos[Mesh Terms] OR placebo$[Title/Abstract] OR random*[Title/Abstract] OR trial$[Title/Abstract]	2,088,406
#5	#1 AND #2 AND #3 AND #4	78
