# Supplementary material for: Efficacy and Safety of Traditional Chinese Medicine Injections for Heart Failure With Reduced Ejection Fraction: A Bayesian Network Meta-Analysis of Randomized Controlled Trials
Source: Front Pharmacol. 2021 Nov 30;12:659707. doi: 10.3389/fphar.2021.659707 (PMC8669995; doi:10.3389/fphar.2021.659707)
Supplement: Supplementary file 7 [file DataSheet1.PDF]

PRISMA 2009 Checklist

Section/topic	#	Checklist item	Reported on page #
**TITLE**			
Title	1	Identify the report as a systematic review, meta-analysis, or both.	Page 1
**ABSTRACT**			
Structured summary	2	Provide a structured summary including, as applicable: background; objectives; data sources; study eligibility criteria, participants, and interventions; study appraisal and synthesis methods; results; limitations; conclusions and implications of key findings; systematic review registration number.	Page 2: Abstract
**INTRODUCTION**			
Rationale	3	Describe the rationale for the review in the context of what is already known.	Page 3: Introduction
Objectives	4	Provide an explicit statement of questions being addressed with reference to participants, interventions, comparisons, outcomes, and study design (PICOS).	Page 3: Introduction, paragraph 3
**METHODS**			
Protocol and registration	5	Indicate if a review protocol exists, if and where it can be accessed (e.g., Web address), and, if available, provide registration information including registration number.	Page 3: Information and Methods, paragraph 1
Eligibility criteria	6	Specify study characteristics (e.g., PICOS, length of follow-up) and report characteristics (e.g., years considered, language, publication status) used as criteria for eligibility, giving rationale.	Page 3: Inclusion and Exclusion Criteria
Information sources	7	Describe all information sources (e.g., databases with dates of coverage, contact with study authors to identify additional studies) in the search and date last searched.	Page 4: Search Strategy
Search	8	Present full electronic search strategy for at least one database, including any limits used, such that it could be repeated.	Additional file 2
Study selection	9	State the process for selecting studies (i.e., screening, eligibility, included in systematic review, and, if applicable, included in the meta-analysis).	Page 4: Literature Screening, Data Extraction, and Risk of Bias Assessment
Data collection process	10	Describe method of data extraction from reports (e.g., piloted forms, independently, in duplicate) and any processes for obtaining and confirming data from investigators.	Page 4: Literature Screening, Data Extraction, and Risk of Bias Assessment
Data items	11	List and define all variables for which data were sought (e.g., PICOS, funding sources) and any assumptions and simplifications made.	Page 4: Literature Screening, Data Extraction, and Risk of Bias Assessment
Risk of bias in individual studies	12	Describe methods used for assessing risk of bias of individual studies (including specification of whether this was done at the study or outcome level), and how this information is to be used in any data synthesis.	Page 4: Literature Screening, Data Extraction, and Risk of Bias Assessment
Summary measures	13	State the principal summary measures (e.g., risk ratio, difference in means).	Page 5: Statistical Analysis

Synthesis of results	14	Describe the methods of handling data and combining results of studies, if done, including measures of consistency (e.g., I^2) for each meta-analysis.	Page 5: Statistical Analysis
Risk of bias across studies	15	Specify any assessment of risk of bias that may affect the cumulative evidence (e.g., publication bias, selective reporting within studies).	Page 5: Statistical Analysis
Additional analyses	16	Describe methods of additional analyses (e.g., sensitivity or subgroup analyses, meta-regression), if done, indicating which were pre-specified.	Page 5: Statistical Analysis
**RESULTS**			
Study selection	17	Give numbers of studies screened, assessed for eligibility, and included in the review, with reasons for exclusions at each stage, ideally with a flow diagram.	Page 6: Figure 1
Study characteristics	18	For each study, present characteristics for which data were extracted (e.g., study size, PICOS, follow-up period) and provide the citations.	Page 8: Table 1
Risk of bias within studies	19	Present data on risk of bias of each study and, if available, any outcome level assessment (see item 12).	Page 14: Figure 3
Results of individual studies	20	For all outcomes considered (benefits or harms), present, for each study: (a) simple summary data for each intervention group (b) effect estimates and confidence intervals, ideally with a forest plot.	Page 12-22: Figure 2, 4, 5; Table 2, 3, 4
Synthesis of results	21	Present results of each meta-analysis done, including confidence intervals and measures of consistency.	Page 5-23: Result
Risk of bias across studies	22	Present results of any assessment of risk of bias across studies (see Item 15).	Page 14: Figure 3
Additional analysis	23	Give results of additional analyses, if done (e.g., sensitivity or subgroup analyses, meta-regression [see Item 16]).	Page 5-23: Result
**DISCUSSION**			
Summary of evidence	24	Summarize the main findings including the strength of evidence for each main outcome; consider their relevance to key groups (e.g., healthcare providers, users, and policy makers).	Page 24:Summary of Main Findings
Limitations	25	Discuss limitations at study and outcome level (e.g., risk of bias), and at review-level (e.g., incomplete retrieval of identified research, reporting bias).	Page 25: Innovations and Limitations of This Study
Conclusions	26	Provide a general interpretation of the results in the context of other evidence, and implications for future research.	Page 25: Conclusion
**FUNDING**			
Funding	27	Describe sources of funding for the systematic review and other support (e.g., supply of data); role of funders for the systematic review.	Page 26: Funding

From: Moher D, Liberati A, Tetzlaff J, Altman DG, The PRISMA Group (2009). Preferred Reporting Items for Systematic Reviews and Meta-Analyses: The PRISMA Statement. PLoS Med 6(7): e1000097. doi:10.1371/journal.pmed1000097

For more information, visit: **www.prisma-statement.org**.

Supplementary File 2 Search Strategy in PubMed

Search	Query	Items found
#1	heart failure[MeSH Terms] OR heart failure[Title/Abstract] OR cardiac failure[MeSH Terms] OR cardiac failure[Title/Abstract] OR systolic heart failure[MeSH Terms] OR systolic heart failure[Title/Abstract] OR heart failure, systolic[MeSH Terms] OR heart failure, systolic[Title/Abstract] OR heart decompensation[MeSH Terms] OR heart decompensation[Title/Abstract] OR cardiac decompensation[MeSH Terms] OR cardiac decompensation[Title/Abstract] OR heart dysfunction[Title/Abstract] OR cardiac dysfunction[Title/Abstract] OR myocardial failure[MeSH Terms] OR myocardial failure[Title/Abstract] OR myocardiac dysfunction[Title/Abstract] OR cardiomyopathy[MeSH Terms] OR cardiomyopathy[Title/Abstract] OR ventricular dysfunction[MeSH Terms] OR ventricular dysfunction[Title/Abstract] OR heart dificiency[Title/Abstract] OR cardiac dificiency[Title/Abstract] OR heart insufficiency[MeSH Terms] OR heart insufficiency[Title/Abstract] OR cardiac insufficiency[MeSH Terms] OR cardiac insufficiency[Title/Abstract]	368,749
#2	Shenfu[All Fields] OR Shenmai[All Fields] OR Xinmailong[All Fields] OR Shengmai[All Fields] OR Huangqi[All Fields] OR Astragalus[All Fields] OR Qiyifumai[All Fields] OR Qiyi Fumai[All Fields]	11,216
#3	injection[MeSH Terms] OR injection$[Title/Abstract] OR injectable$[Title/Abstract]	708,382
#4	randomized controlled trial[Publication Type] OR controlled clinical trial[Publication Type] OR clinical trial[Publication Type] OR equivalence trial[Publication Type] OR randomized controlled trials as topic[Mesh Terms] OR randomized controlled trial[Mesh Terms] OR randomized controlled trial[Title/Abstract] OR clinical trials, randomized[Mesh Terms] OR clinical trials, randomized[Title/Abstract] OR controlled clinical trials, randomized[Mesh Tems] OR controlled clinical trials, randomized[Title/Abstract] OR random allocation[Mesh Terms]OR random allocation[Title/Abstract] OR double-blind method[Mesh Terms] OR double-blind method[Title/Abstract] OR single-blind method[Mesh Terms] OR single-blind method[Title/Abstract] OR placebos[Mesh Terms] OR placebo$[Title/Abstract] OR random*[Title/Abstract] OR trial$[Title/Abstract]	2,088,406
#5	#1 AND #2 AND #3 AND #4	78

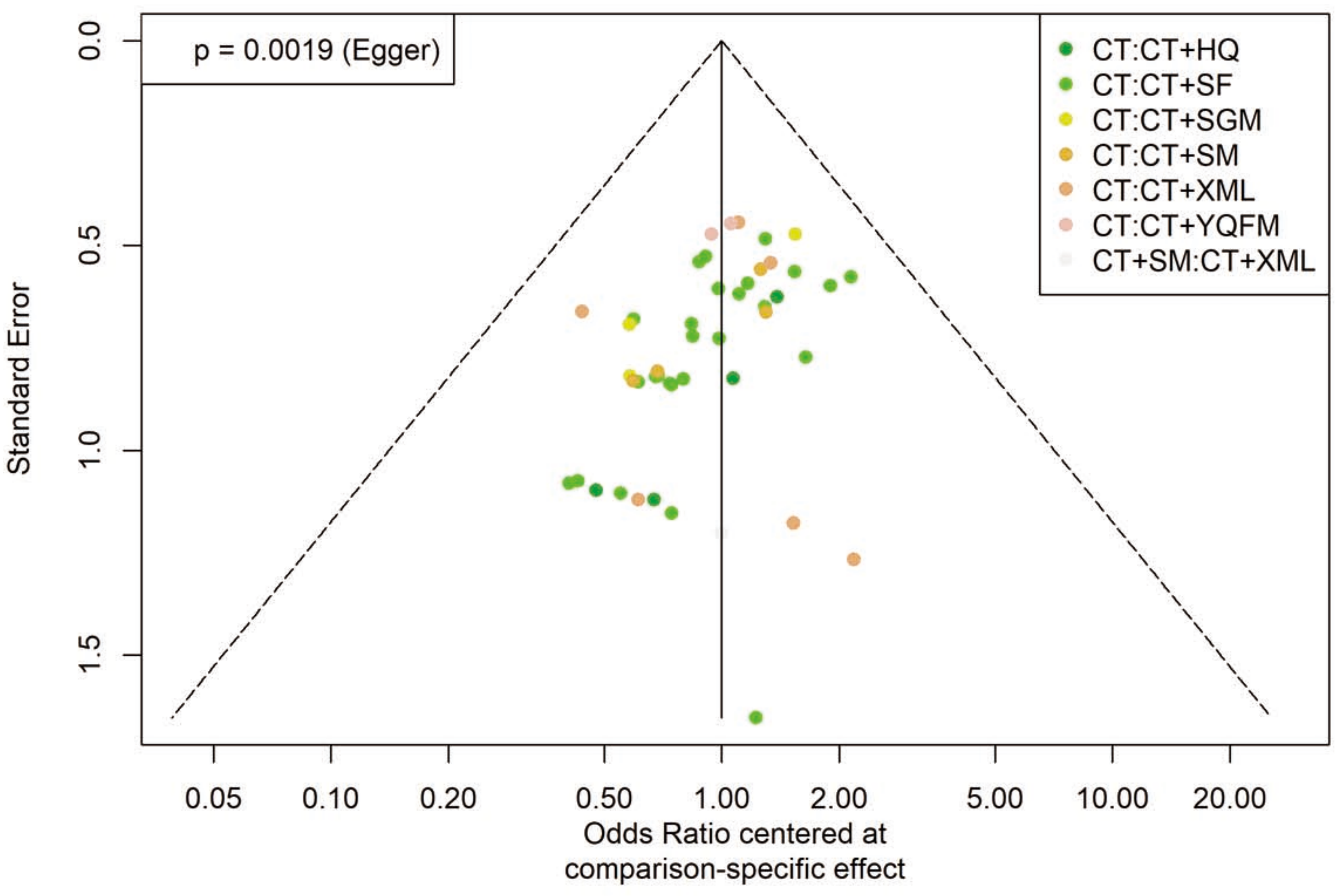

NYHA

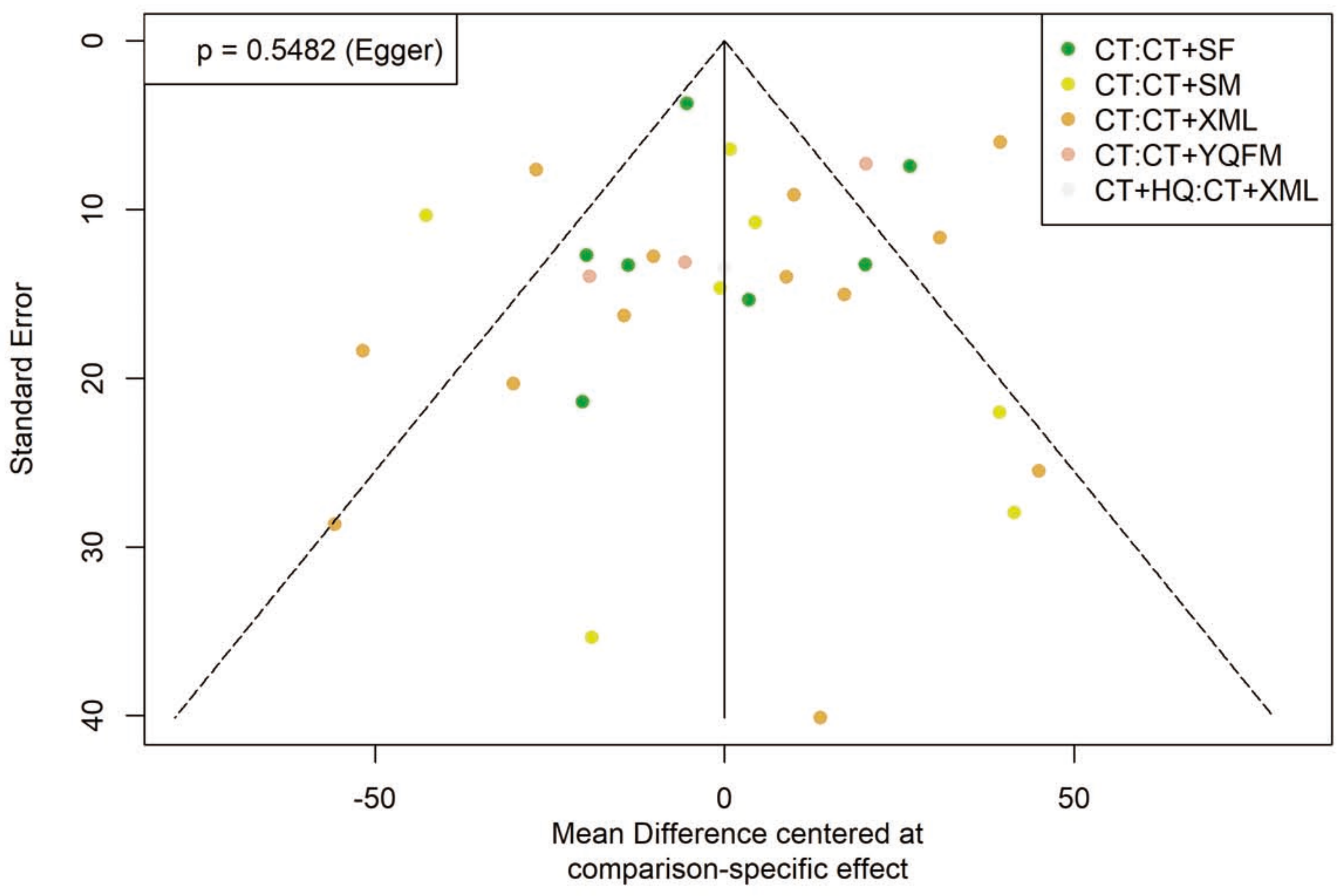

6WMT

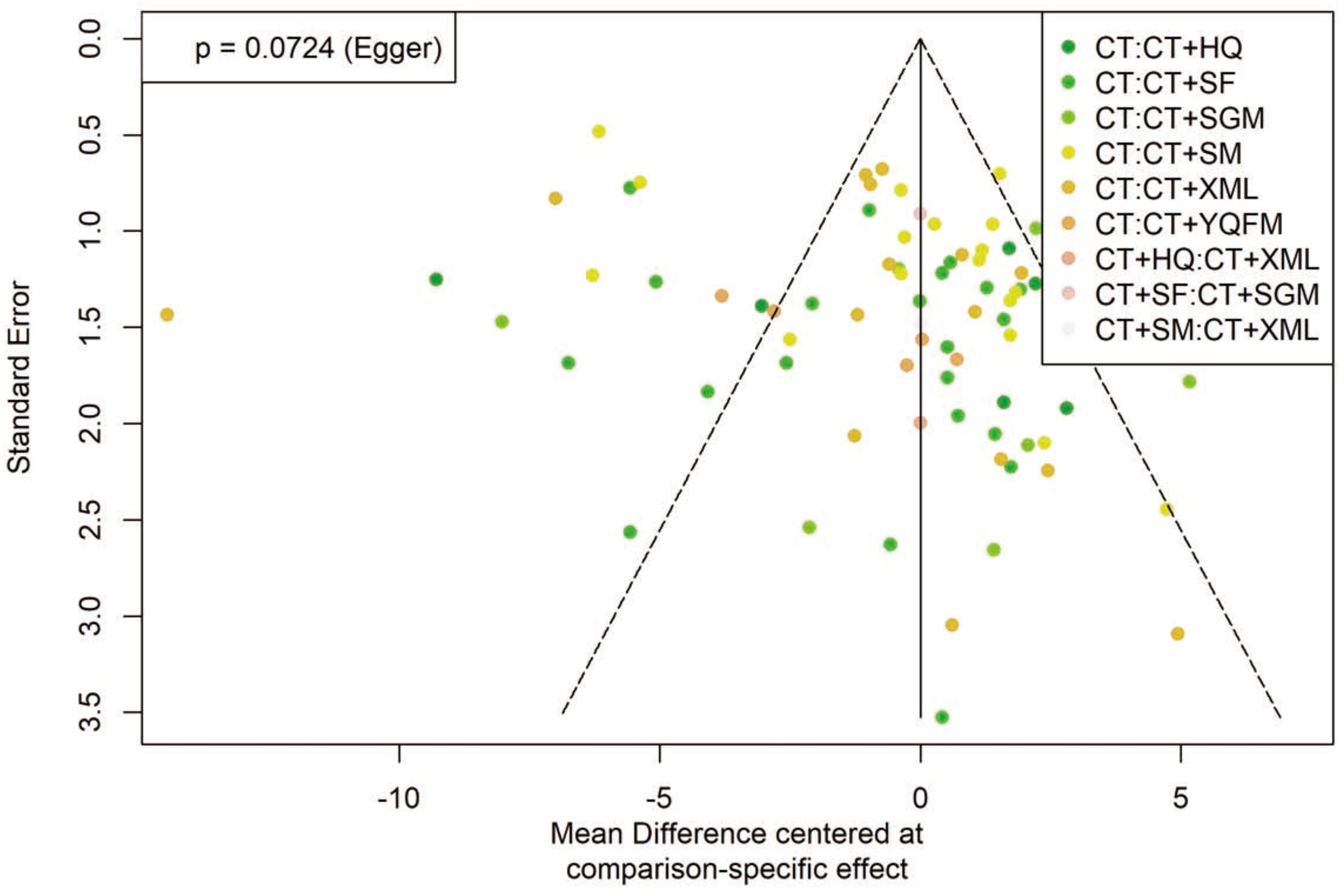

LVEF

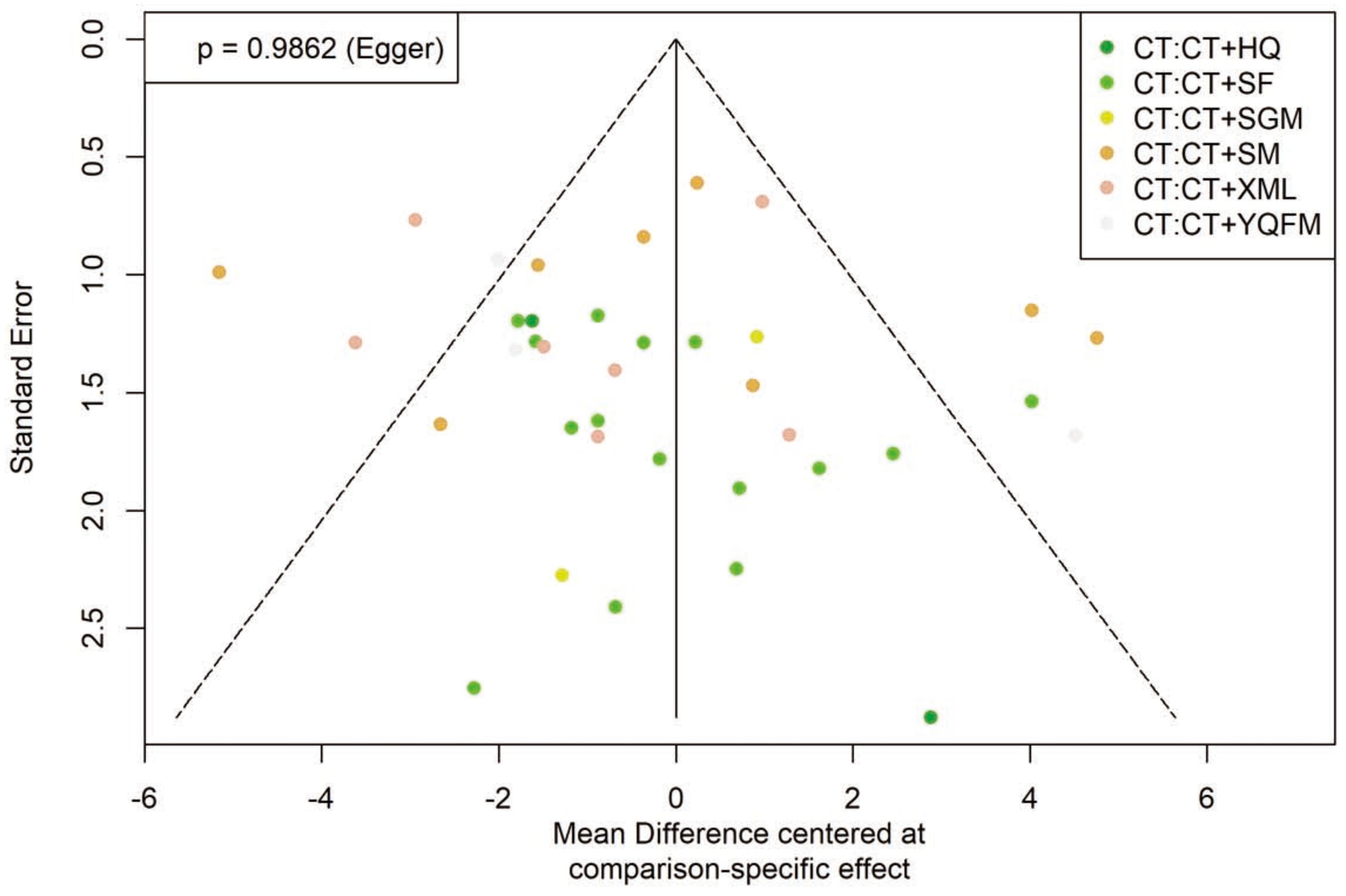

LVEDD

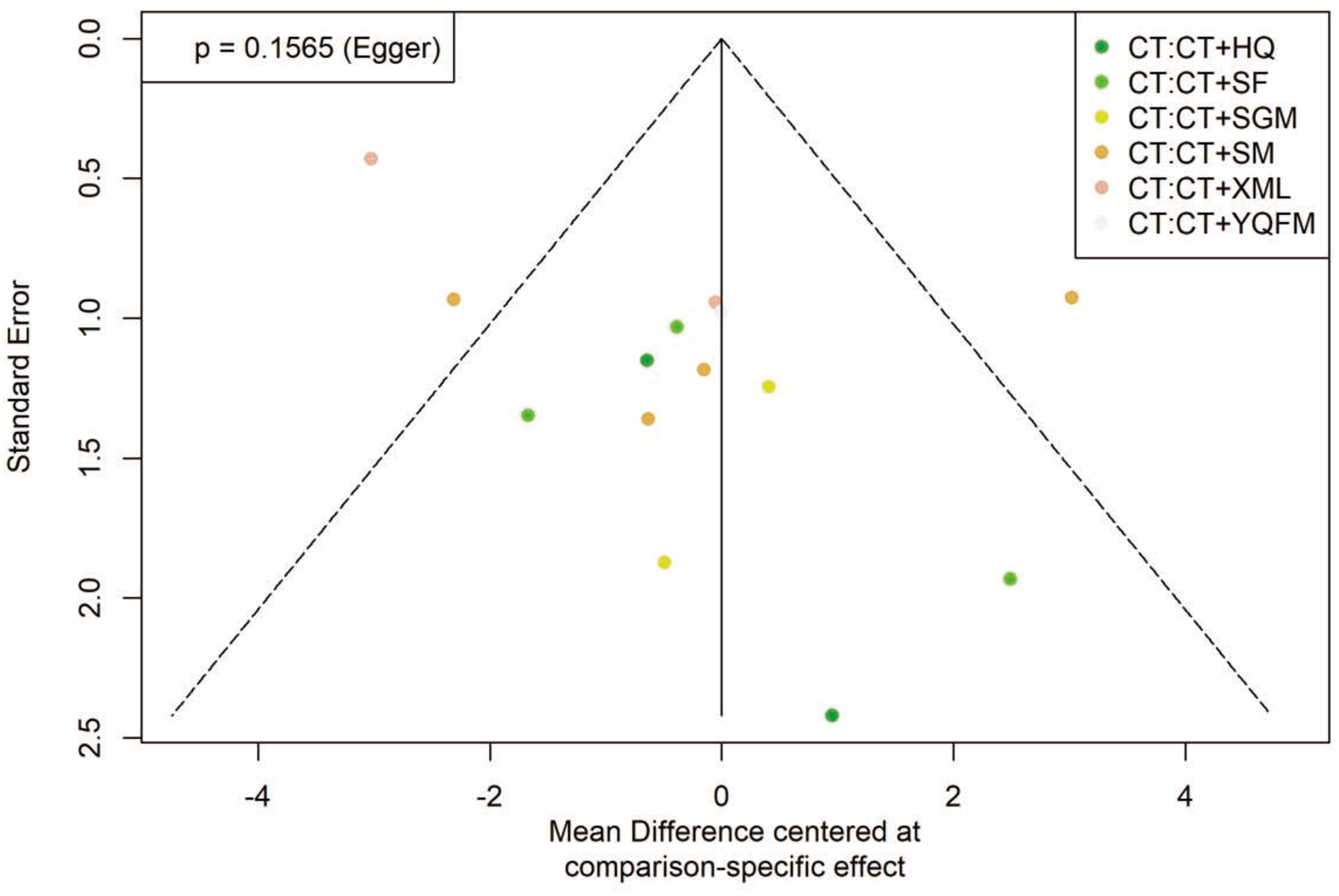

LVESD

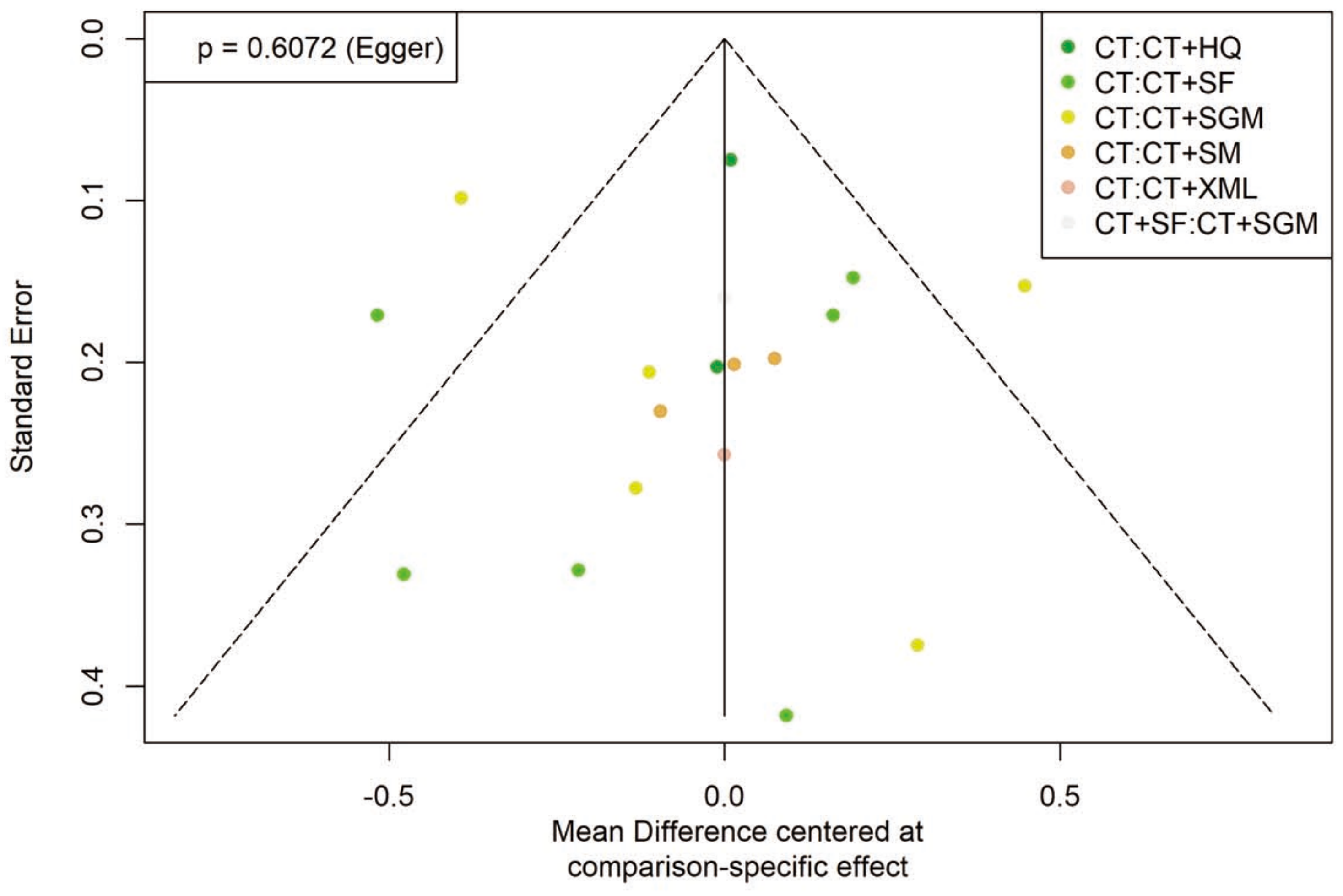

CO

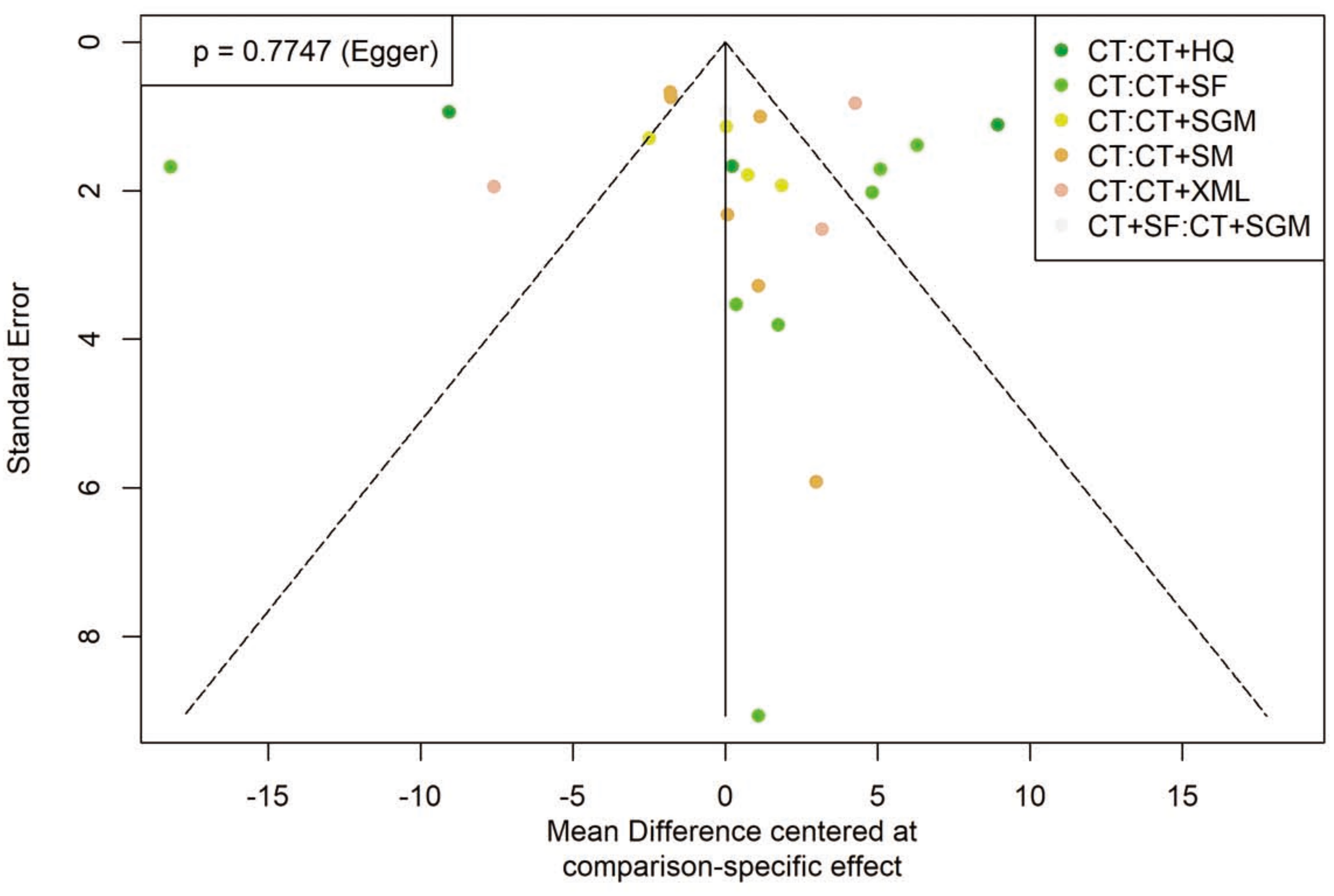

SV

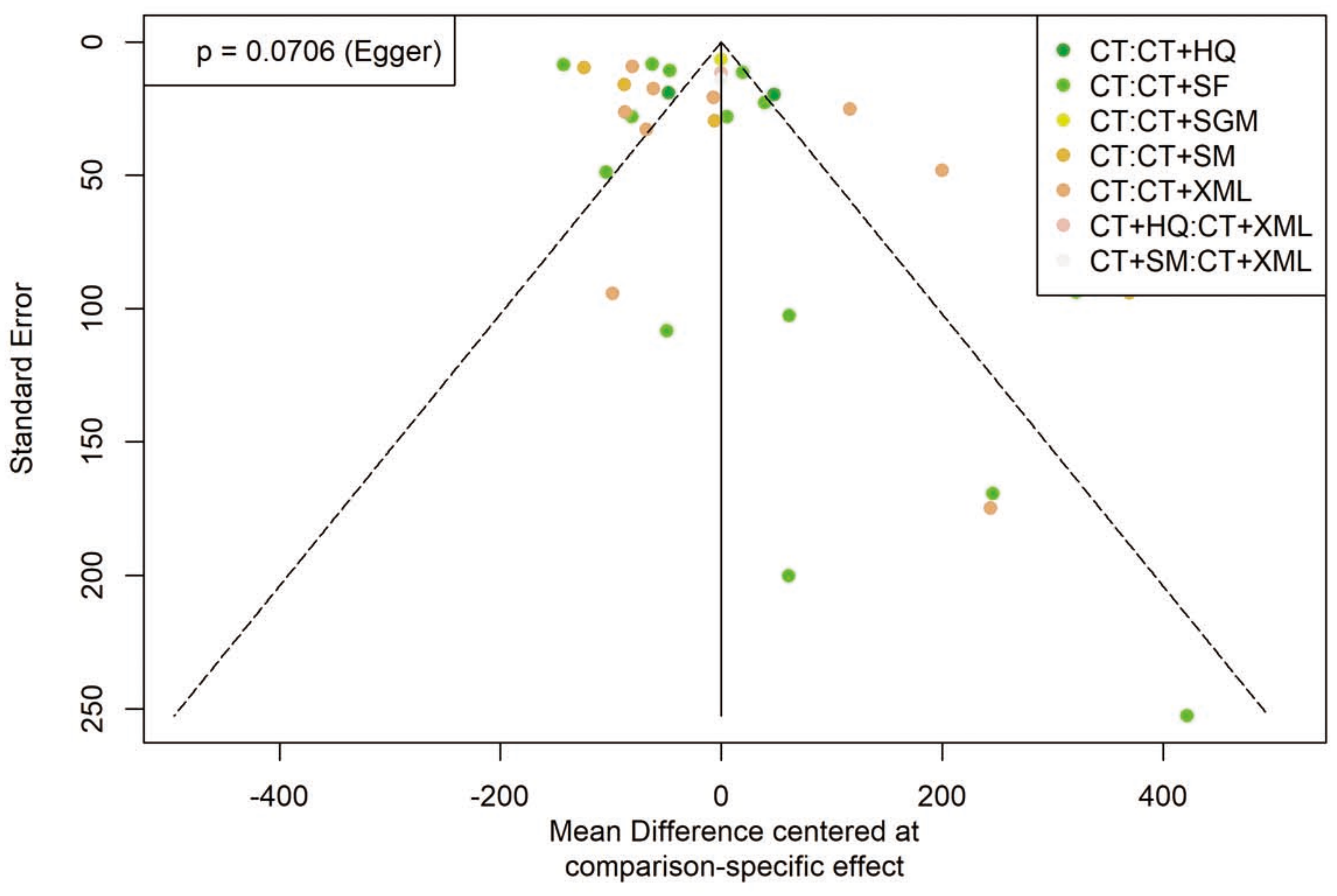

BNP

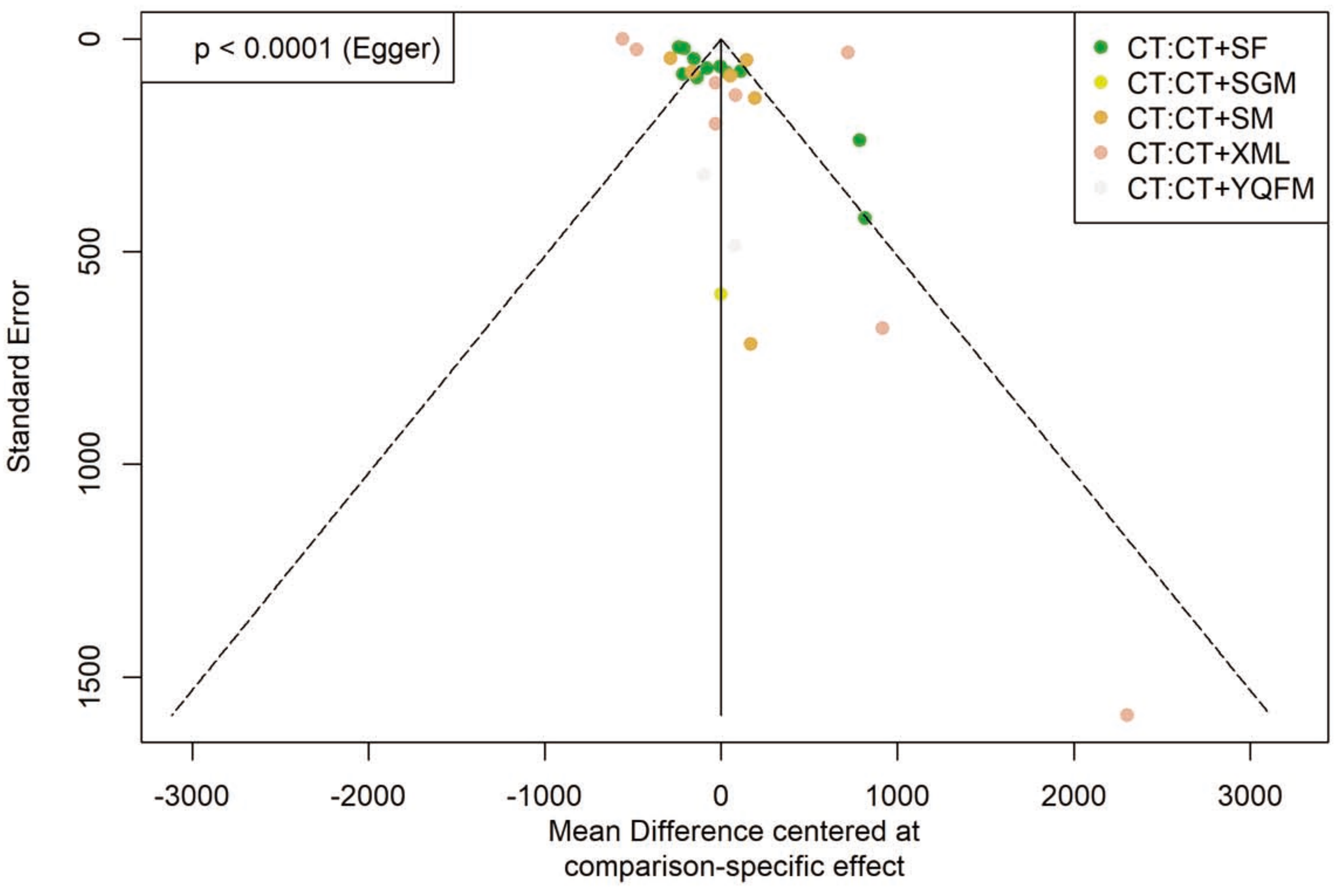

NT-proBNP

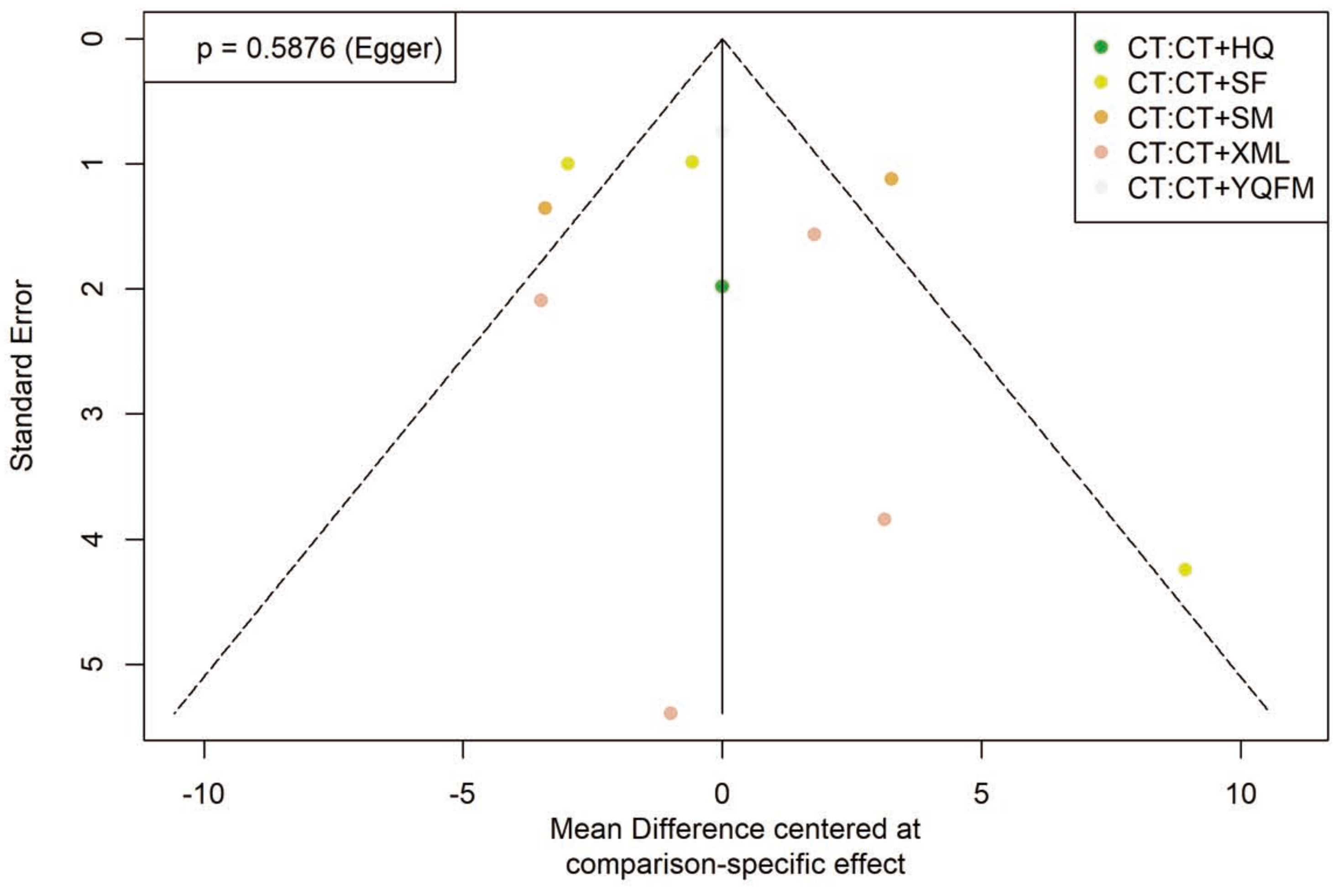

MLHFQ

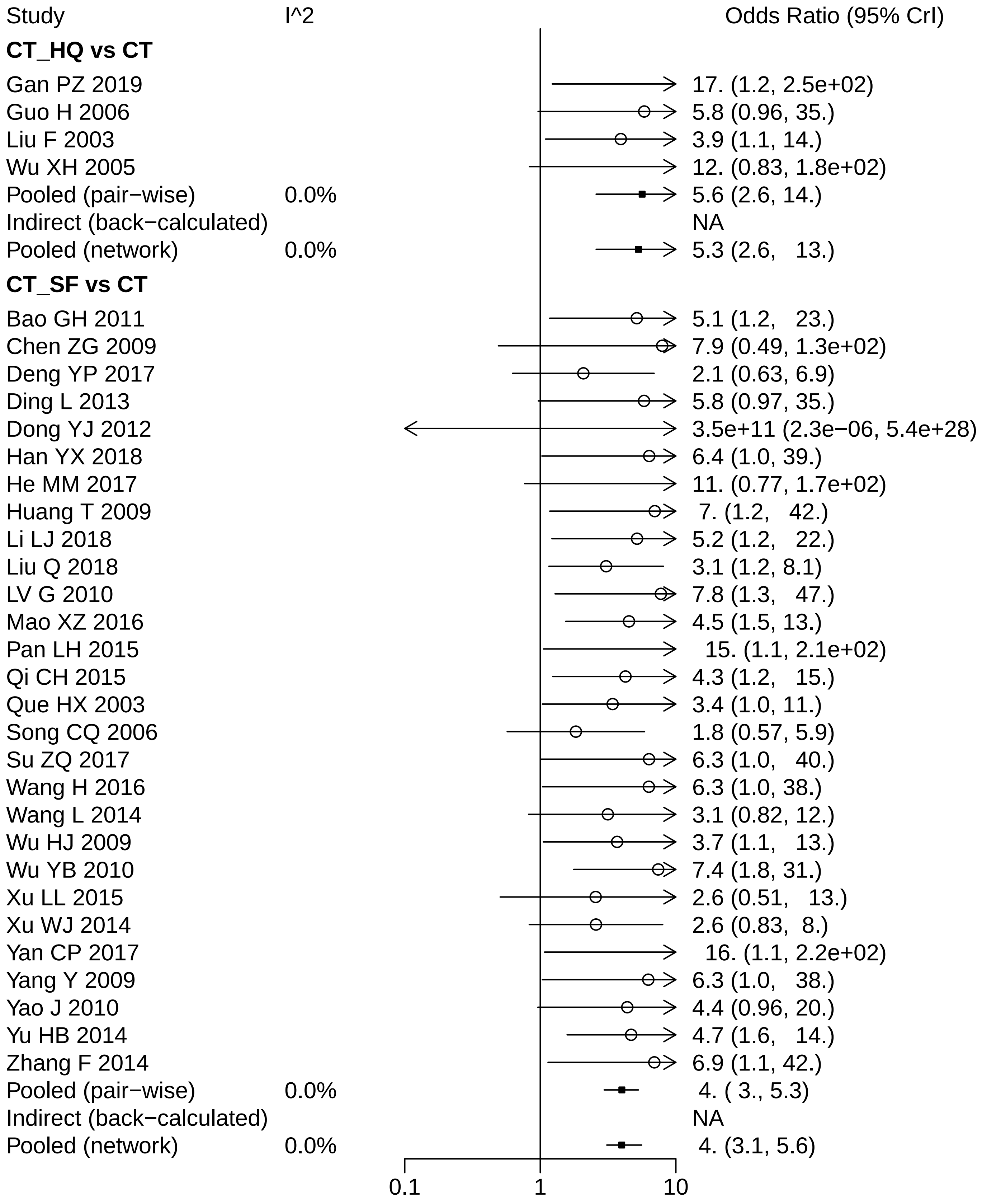

Study
I^2
Odds Ratio (95% CrI)
CT_HQ vs CT
Gan PZ 2019 17. (1.2, 2.5e+02)
Guo H 2006 5.8 (0.96, 35.)
Liu F 2003 3.9 (1.1, 14.)
Wu XH 2005 12. (0.83, 1.8e+02)
Pooled (pair−wise) 0.0% 5.6 (2.6, 14.)
Indirect (back−calculated) NA
Pooled (network) 0.0% 5.3 (2.6, 13.)
CT_SF vs CT
Bao GH 2011 5.1 (1.2, 23.)
Chen ZG 2009 7.9 (0.49, 1.3e+02)
Deng YP 2017 2.1 (0.63, 6.9)
Ding L 2013 5.8 (0.97, 35.)
Dong YJ 2012 3.5e+11 (2.3e−06, 5.4e+28)
Han YX 2018 6.4 (1.0, 39.)
He MM 2017 11. (0.77, 1.7e+02)
Huang T 2009 7. (1.2, 42.)
Li LJ 2018 5.2 (1.2, 22.)
Liu Q 2018 3.1 (1.2, 8.1)
LV G 2010 7.8 (1.3, 47.)
Mao XZ 2016 4.5 (1.5, 13.)
Pan LH 2015 15. (1.1, 2.1e+02)
Qi CH 2015 4.3 (1.2, 15.)
Que HX 2003 3.4 (1.0, 11.)
Song CQ 2006 1.8 (0.57, 5.9)
Su ZQ 2017 6.3 (1.0, 40.)
Wang H 2016 6.3 (1.0, 38.)
Wang L 2014 3.1 (0.82, 12.)
Wu HJ 2009 3.7 (1.1, 13.)
Wu YB 2010 7.4 (1.8, 31.)
Xu LL 2015 2.6 (0.51, 13.)
Xu WJ 2014 2.6 (0.83, 8.)
Yan CP 2017 16. (1.1, 2.2e+02)
Yang Y 2009 6.3 (1.0, 38.)
Yao J 2010 4.4 (0.96, 20.)
Yu HB 2014 4.7 (1.6, 14.)
Zhang F 2014 6.9 (1.1, 42.)
Pooled (pair−wise) 0.0% 4. (3., 5.3)
Indirect (back−calculated) NA
Pooled (network) 0.0% 4. (3.1, 5.6)
0.1
1
10

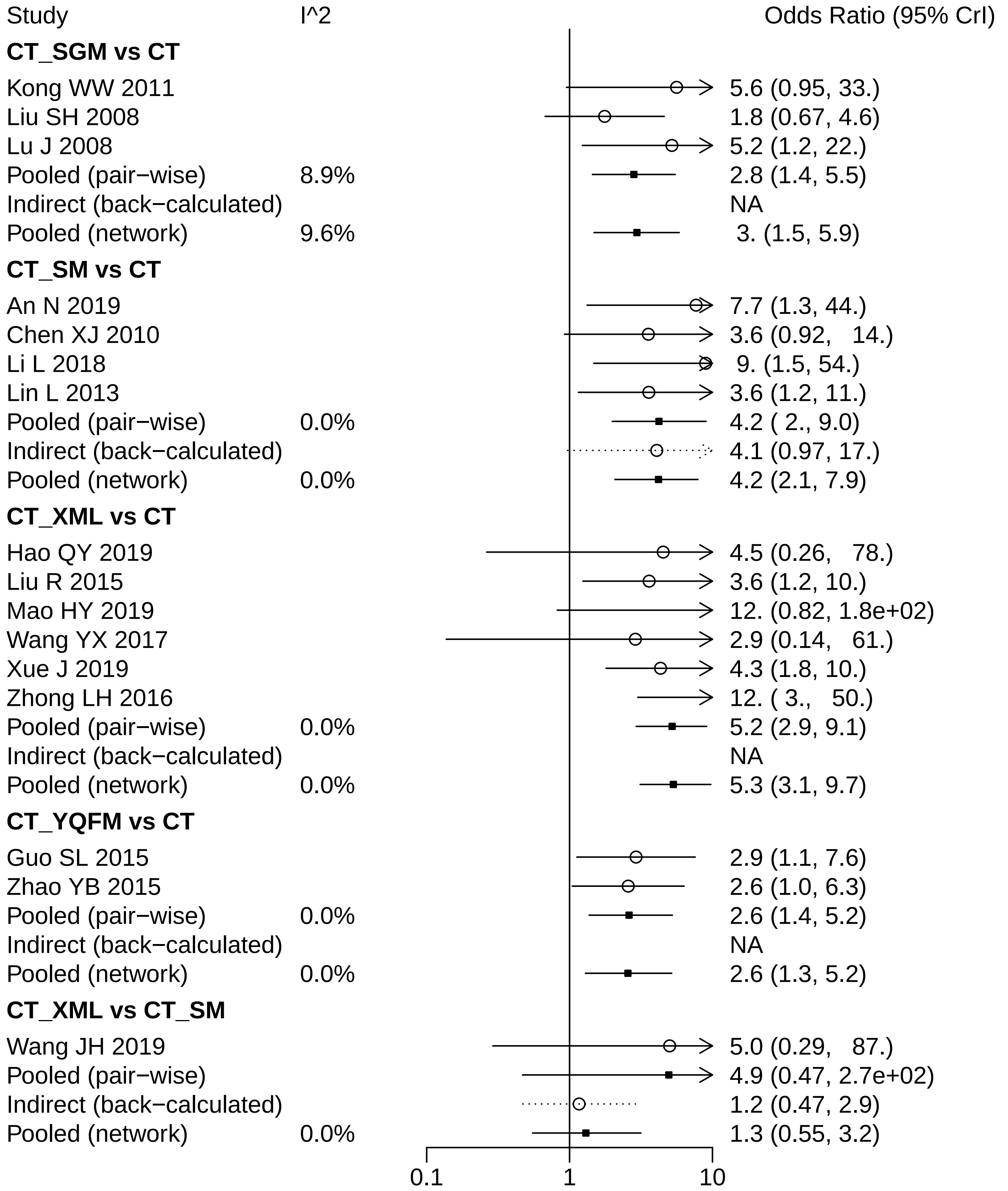

Study
I^2
Odds Ratio (95% CrI)
CT_SGM vs CT
Kong WW 2011 5.6 (0.95, 33.)
Liu SH 2008 1.8 (0.67, 4.6)
Lu J 2008 5.2 (1.2, 22.)
Pooled (pair−wise) 8.9% 2.8 (1.4, 5.5)
Indirect (back−calculated) NA
Pooled (network) 9.6% 3. (1.5, 5.9)
CT_SM vs CT
An N 2019 7.7 (1.3, 44.)
Chen XJ 2010 3.6 (0.92, 14.)
Li L 2018 9. (1.5, 54.)
Lin L 2013 3.6 (1.2, 11.)
Pooled (pair−wise) 0.0% 4.2 (2., 9.0)
Indirect (back−calculated) 4.1 (0.97, 17.)
Pooled (network) 0.0% 4.2 (2.1, 7.9)
CT_XML vs CT
Hao QY 2019 4.5 (0.26, 78.)
Liu R 2015 3.6 (1.2, 10.)
Mao HY 2019 12. (0.82, 1.8e+02)
Wang YX 2017 2.9 (0.14, 61.)
Xue J 2019 4.3 (1.8, 10.)
Zhong LH 2016 12. (3., 50.)
Pooled (pair−wise) 0.0% 5.2 (2.9, 9.1)
Indirect (back−calculated) NA
Pooled (network) 0.0% 5.3 (3.1, 9.7)
CT_YQFM vs CT
Guo SL 2015 2.9 (1.1, 7.6)
Zhao YB 2015 2.6 (1.0, 6.3)
Pooled (pair−wise) 0.0% 2.6 (1.4, 5.2)
Indirect (back−calculated) NA
Pooled (network) 0.0% 2.6 (1.3, 5.2)
CT_XML vs CT_SM
Wang JH 2019 5.0 (0.29, 87.)
Pooled (pair−wise) 4.9 (0.47, 2.7e+02)
Indirect (back−calculated) 1.2 (0.47, 2.9)
Pooled (network) 0.0% 1.3 (0.55, 3.2)
0.1
1
10

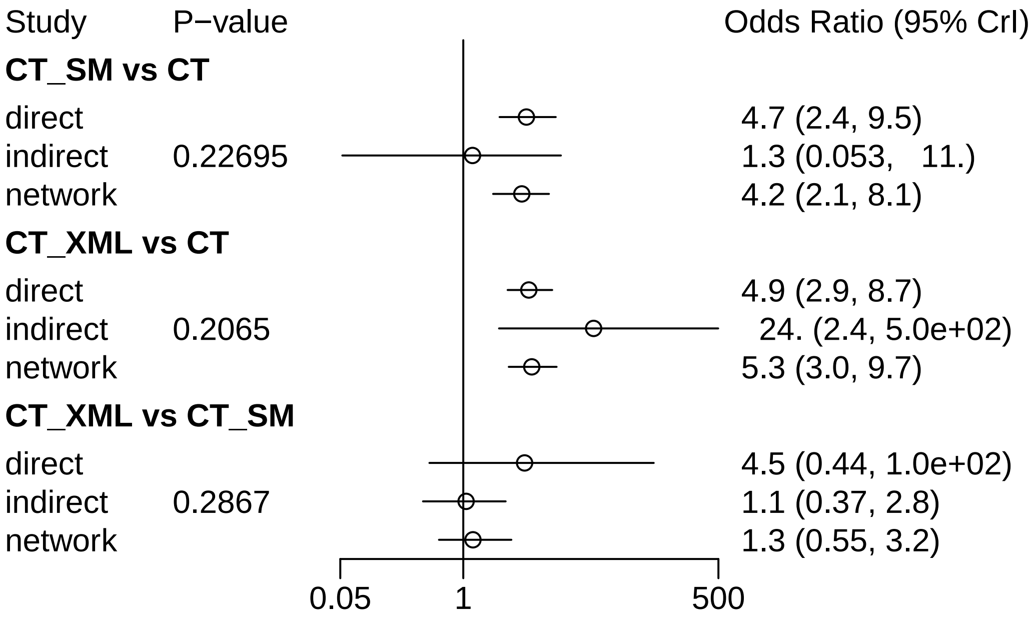
Study
P−value
Odds Ratio (95% CrI)
CT_SM vs CT
direct
4.7 (2.4, 9.5)
indirect
0.22695
1.3 (0.053, 11.)
network
4.2 (2.1, 8.1)
CT_XML vs CT
direct
4.9 (2.9, 8.7)
indirect
0.2065
24. (2.4, 5.0e+02)
network
5.3 (3.0, 9.7)
CT_XML vs CT_SM
direct
4.5 (0.44, 1.0e+02)
indirect
0.2867
1.1 (0.37, 2.8)
network
1.3 (0.55, 3.2)
0.05
1
500
